# Optimized distributed systems achieve significant performance improvement on sorted merging of massive VCF files

**DOI:** 10.1093/gigascience/giy052

**Published:** 2018-05-10

**Authors:** Xiaobo Sun, Jingjing Gao, Peng Jin, Celeste Eng, Esteban G Burchard, Terri H Beaty, Ingo Ruczinski, Rasika A Mathias, Kathleen Barnes, Fusheng Wang, Zhaohui S Qin, CAAPA consortium

**Affiliations:** 1 Department of Computer Sciences, Emory University, Atlanta, GA 30322, USA; 2 Department of Medical Informatics, Emory University School of Medicine, Atlanta, GA 30322, USA; 3 Department of Human Genetics, Emory University School of Medicine, Atlanta, GA 30322, USA; 4 Department of Medicine, University of California, San Francisco, San Francisco, CA 94143 USA; 5 Department of Epidemiology, Bloomberg School of Public Health, JHU, Baltimore, MD 21205 USA; 6 Department of Biostatistics, Bloomberg School of Public Health, JHU, Baltimore, MD 21205 USA; 7 Department of Medicine, Johns Hopkins University, Baltimore, MD 21224 USA; 8 Department of Medicine, University of Colorado, Denver, Aurora, CO, 80045 USA; 9 Department of Biomedical Informatics, Stony Brook University, Stony Brook, NY 11794, USA; 10 Department of Biostatistics, Emory University, Atlanta, GA 30322, USA; 11 See end of the paper for a complete list of CAAPA members

**Keywords:** *sorted merging*, *whole-genome sequencing*, *MapReduce*, *Hadoop*, *HBase*, *Spark*

## Abstract

**Background:**

Sorted merging of genomic data is a common data operation necessary in many sequencing-based studies. It involves sorting and merging genomic data from different subjects by their genomic locations. In particular, merging a large number of variant call format (VCF) files is frequently required in large-scale whole-genome sequencing or whole-exome sequencing projects. Traditional single-machine based methods become increasingly inefficient when processing large numbers of files due to the excessive computation time and Input/Output bottleneck. Distributed systems and more recent cloud-based systems offer an attractive solution. However, carefully designed and optimized workflow patterns and execution plans (schemas) are required to take full advantage of the increased computing power while overcoming bottlenecks to achieve high performance.

**Findings:**

In this study, we custom-design optimized schemas for three Apache big data platforms, Hadoop (MapReduce), HBase, and Spark, to perform sorted merging of a large number of VCF files. These schemas all adopt the divide-and-conquer strategy to split the merging job into sequential phases/stages consisting of subtasks that are conquered in an ordered, parallel, and bottleneck-free way. In two illustrating examples, we test the performance of our schemas on merging multiple VCF files into either a single TPED or a single VCF file, which are benchmarked with the traditional single/parallel multiway-merge methods, message passing interface (MPI)–based high-performance computing (HPC) implementation, and the popular VCFTools.

**Conclusions:**

Our experiments suggest all three schemas either deliver a significant improvement in efficiency or render much better strong and weak scalabilities over traditional methods. Our findings provide generalized scalable schemas for performing sorted merging on genetics and genomics data using these Apache distributed systems.

## Introduction

With the rapid development of high-throughput biotechnologies, genetic studies have entered the Big Data era. Studies like genome-wide association studies (GWASs), whole-genome sequencing (WGS), and whole-exome sequencing studies have produced massive amounts of data. The ability to efficiently manage and process such massive amounts of data becomes increasingly important for successful large-scale genetics studies [1–3]. Single-machine based methods are inefficient when processing such large amounts of data due to the prohibitive computation time, Input/Output bottleneck, as well as central processing unit (CPU) and memory limitations. Traditional high-performance computing (HPC) techniques based on message passing interface (MPI)/OpenMP also suffer from limitations such as not allowing addition of computing nodes at runtime, shortage of a fault-tolerant and high available file system, and inflexibility of customizing the computing environment without administrator permission of a cluster [3, 4]. It becomes increasingly attractive for investigators to take advantage of more powerful distributed computing resources or the cloud to perform data processing and analyses [3, 5]. The Apache Foundation has been a leading force in this endeavor and has developed multiple platforms and systems including Hadoop [6, 7], HBase [8], and Spark [9]. All these three Apache platforms have gained substantial popularity in recent years and have been endorsed and supported by major vendors such as Amazon Web Services (AWS).

In bioinformatics, researchers have already started to embrace Apache distributed systems to manage and process large amounts of high throughput “-omics” data. For example, the Cancer Genome Atlas project makes use of the Hadoop framework to split genome data into chunks distributed over the cluster for parallel processing [3, 10]. The CloudBurst [11], Seal [12], Hadoop-BAM [13], and Crossbow software [14] take advantage of the Hadoop framework to accelerate sequencing read mapping, aligning, and manipulations as well as single-nucleotide polymorphism (SNP) calling. The Collaborative Genomic Data Model [15] adopts HBase to boost the querying speed for the main classes of queries on genomic databases. MetaSpark [16] utilizes Spark's distributed dataset to recruit large scales of metagenomics reads to reference genomes and achieves better scalability and sensitivity than single-machine based programs [17]. Industry cloud computing vendors such as Amazon [18] and Google [19] are also beginning to provide specialized environments to ease genomics data processing in the cloud.

Although numerous Apache cluster-based applications have already been developed for processing and analyzing large-scale genomics data including ADAM [1], VariantSpark [20], SparkSeq [21], Halvade [22], and SeqHBase [23], among others, we believe there are still many opportunities in biomedical data analyses to take advantage of distributed systems as the scale and scope of data become larger and more complex. A particular example is sorted merging, which is a ubiquitous operation in processing genetics and genomics data. As an example, in WGS, variants identified from individuals are often called and stored in separate variant call format (VCF) files. Eventually these VCF files need to be merged (into a VCF or TPED file) as required by downstream analysis tools such as PLINK [24] and BlueSNP [25, 26]. Either a VCF or TPED file requires the data to be sorted by their genomic locations, thus these tasks are equivalent to the well-known sorted full-outer-joining problem [27]. Currently, they are handled by software such as VCFTools [28] and PLINK, which become considerably inefficient even in the face of a moderate number of VCF files. The main reason is that these tools adopt the multiway-merge-like method [29] with a priority queue as the underlying data structure to ensure the correct output order. Although such a method only requires one round of read through of the input files, a key deficiency is that it can only have one consumer access items from the data queue, which makes it sequential upon writing. This problem cannot be eliminated even if the multiway-merging is implemented as parallel processes due to I/O saturation, workload imbalance among computing units, and memory limits. Therefore, these single-machine based tools are inefficient and time-consuming when handling large datasets.

In this study, we use the case of sorted-merging multiple VCF files to demonstrate the benefits of using Apache distributed platforms. However, simply running sorted merging on such distributed systems runs into problems of bottlenecks, hot spots, and unordered results commonly seen in parallel computations. Rather, we believe working schemas custom designed for each specific distributed platform are required to unleash their full potential. To overcome the limitations of single-machine, traditional parallel/distributed, and simple Apache distributed system based methods, we propose and implement three schemas running on Hadoop, Spark, and HBase, respectively. We chose these three platforms because they represent cloud distributed systems providing data partitioning based parallelism with distributed storage, data partitioning based parallelism with in-memory based processing, and high dimensional tables like distributed storage, respectively. Hadoop [6] is the open source implementation of MapReduce [7] based on the parallel key-value processing technique and has the advantage of transparency and simplicity. HBase [8] is a data warehousing platform that adopts Google's BigTable data storing structure [30] to achieve high efficiency in storing and reading/writing large scales of sparse data. Spark [9] introduces the concept of resilient distributed dataset (RDD) and directed acyclic graph execution to parallel key-value processing, thus enabling fast, robust, and repetitive in-memory data manipulations. Specifically, our schemas involve dividing the job into multiple phases corresponding to tasks of loading, mapping, filtering, sampling, partitioning, shuffling, merging, and outputting. Within each phase, data and tasks are evenly distributed across the cluster, enabling processing large amounts of data in a parallel and scalable manner, which in turn improves both speed and scalability.

## Methods

### Overview

The benefits of using these three Apache distributed platforms to perform sorted merging are four-fold when compared to using the multiway-merge method [29], a relational database based approach, or an HPC framework. First, with genomic locations as keys and genotypes as values, it is readily transformed into the key-value model in which all three platforms offer a rich set of parallel operations. Second, data in VCF files are semi-structured. This type of data is an ideal fit for the three platforms that allow defining the schema during data loading, avoiding the preprocessing of raw data into a rigid schema as in a relational database. Third, all of these platforms provide built-in efficient task coordination, high fault tolerance, data availability, and locality, which are absent in the traditional HPC framework. Fourth, the merged results are directly saved onto a distributed file system such as Hadoop distributed file systems (HDFS) or Amazon S3, which can be directly used for subsequent cluster-based GWAS or WGS analytical tools such as BlueSNP.

Despite these advantages, simply performing sorted merging on these Apache distributed systems will not deliver the expected results for the following reasons. First, it can lead to globally unsorted results. Hash-based shuffling of input data is the default mechanism for distributing data to parallel working units in the system. However, shuffling will lead to globally unsorted results. Second, bottlenecks and hot spots can happen during the processing in the cluster. Bypassing the hashing based shuffling can lead to unbalanced workloads across the cluster and result in straggling computing units that become bottlenecks for response time. In addition, for parallel loading of presorted data into HBase, data being loaded from all the loading tasks access the same node simultaneously, while other nodes may be idling, creating an I/O hot spot. Third, sampling costs could become prohibitive. Although Hadoop provides a built-in utility named *total-order-merging* [27] to achieve both workload balance and global order, it involves transferring to and sampling all the data on a single node. The communication costs over the network and disk I/O can be prohibitive when data size becomes very large. In the following sections, we will illustrate how our custom designed schemas are able to overcome these limitations in detail.

### Data formats and operations

**Figure 1:**
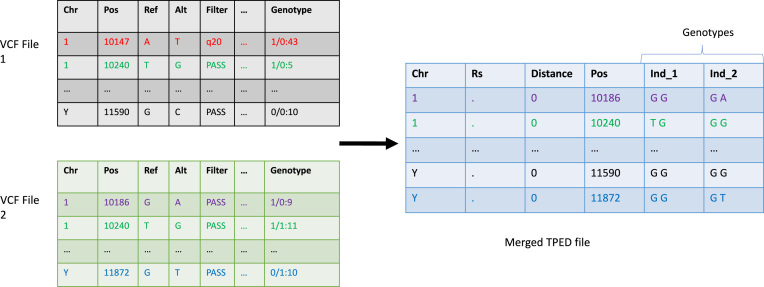
Merging multiple VCF files into a single TPED file. Left tables represent input VCF files. The table on the right represents the merged TPED file. Records are filtered out if their Filter value is not equal to “PASS” (Pos 10 147). Individual genotypes from multiple VCF files with the same genomic location are aggregated together in one row. The resulting TPED file thus has an inclusive set of sorted genomic locations of all variants found in the input VCF files.

In a typical WGS experiment, data analysis often starts from individual genotype files in the VCF format [31]. A VCF file contains data arranged into a table consisting of eight mandatory fields including chromosome (CHROM), the genomic coordinate of the start of the variant (POS), the reference allele (REF), and a comma separated list of alternate alleles (ALT), among others. In our experiments, we use a dataset consisting of the VCF files of 186 individuals [32] generated from Illumina's BaseSpace software (left tables in Fig. 1). Each VCF file has around 4–5 million rows, each row contains information on one of the individual's genomic variants. Each VCF file is about 300 MB in size. In an attempt to protect privacy of study subjects, we apply the following strategy to conceal their real genetic variant information contained in the VCF files: we first transform each original genomic location by multiplying it with an undisclosed constant real number, taking the floor integer of the result, and then add another undisclosed constant integer number.

It is common that multiple VCF files need to be merged into a single TPED file for analysis tools such as PLINK. A TPED file resembles a big table, aggregating genotypes of all individuals under investigation by genomic locations (right table in Fig. 1). The merging follows several rules. First, each record is associated with a data quality value in the FILTER column, which records the status of this genomic position passing all filters. Usually only qualified records with a “PASS” filter value are retained. Second, genotypes in VCF files are stored in the form of allele values, where 0 stands for the reference allele, 1 stands for the first mutant allele, 2 stands for the second mutant allele, and so on. Allele values must be translated into corresponding types of nucleotides in the TPED file. Third, all individuals need to have a genotype for genomic locations appearing in at least one VCF file. The default genotype for a missing value is a pair of homozygous reference alleles. The merging of multiple VCF files into a single VCF file follows the rules as follows: first, the ALT and INFO columns of a genomic location in the merged file are set as the concatenated values of the corresponding columns on that location from all input files with duplicated values removed. Second, the QUAL column of a genomic location in the merged file is set as a weight-averaged quality value of all individuals on that location. Third, a genomic location is kept only when it appears in at least one input file and has a FILTER column value of “PASS.” Fourth, if an individual does not have allele values on a genomic location in the input file, their missing allele values are designated as “.” in the merged file.

For our Apache cluster-based schemas, the merging of multiple VCF files into a single TPED file and the merging of multiple VCF files into a single VCF file differ only in the value contents of the key-value pairs, so they should have the same scalability property. Although we implement the applications of both merging types using our Apache cluster-based schemas, which are available on our project website, we focused our experiments on the merging of multiple VCF files into a single TPED file and only evaluate the execution speed of the merging of multiple VCF files into a single VCF file with VCFTools as the benchmark.

### MapReduce (Hadoop) schema

**Figure 2:**
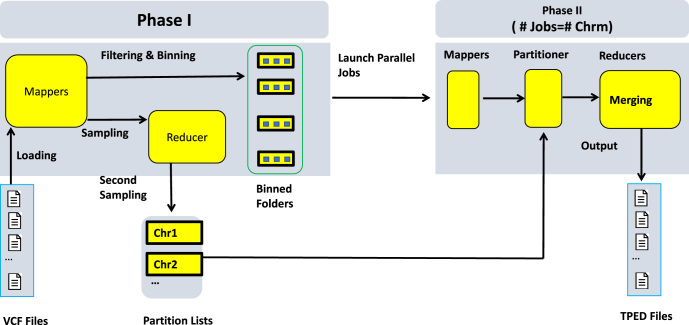
The workflow chart of the MapReduce schema. The workflow is divided into two phases. In the first phase, variants are filtered, grouped by chromosomes into bins, and mapped into key-value records. Two sampling steps are implemented to generate partition lists of all chromosomes. In the second phase, parallel jobs of specified chromosomes are launched. Within each job, records from corresponding bins are loaded, partitioned, sorted, and merged by genomic locations before being saved into a TPED file.

This schema is built on Hadoop's underlying model MapReduce and running on Hadoop clusters. MapReduce [7] is a parallel computing model based on a *split-apply-combine* strategy for data analysis, in which data are mapped to key-values for splitting (mapping), shuffling, and combining (reducing) for final results. We use Apache Hadoop-2.7 as the system for our implementation. Our optimized schema consists of two MapReduce phases, as shown in Fig. 2 (the pseudocodes are shown in Supplementary Fig. S1).

#### First MapReduce phase

Raw data are loaded from HDFS into parallel mappers to perform the following tasks. First, unqualified data are filtered out and qualified data are mapped to key-value pairs. The mapper output key is the genomic location and the output value is the genotype and individual ID. Second, key-value pairs are grouped together by chromosomes and temporarily saved as compressed Hadoop sequence files [33] for faster I/O in the second MapReduce phase. With this grouping, if SNPs of interest are located in a few selected chromosomes only, we can choose to just merge records from these selected chromosomes rather than from all chromosomes. Meanwhile, these records are sampled to explore their distribution profile of keys along chromosomes to determine boundaries. The boundaries are determined so there is an approximately equal number of records within each segment. Because all records falling in the same segment will be assigned to the same reducer in a later phase, boundaries calculated in this way ensure the workload of each reducer is balanced. There are two rounds of samplings. The first one happens in each mapper with a pre-specified sampling rate, which in our case is set to be 0.0001. Sampled records are then separated and distributed to different reducers in this phase by chromosomes, where they are sampled again with a rate equal to the reciprocal of the number of input files. This second sampling effectively limits the number of final sampled records even in the face of a very large number of input files. Because the number of reducers instantiated in the second phase equals the number of boundaries, which in turn is decided by the number of sampled records, we can therefore avoid launching unnecessary reducers, thus minimizing task overheads.

#### Second MapReduce phase

In this phase, multiple parallel MapReduce jobs are created, one for each chromosome, to handle all the records in sequence files generated from the first phase. Within each job, a partitioner redirects records to the appropriate reducer by referring to the splitting boundaries from the previous phase, so records falling in between the same pair of boundaries are aggregated together. Finally, each reducer sorts and merges aggregated records by genomic locations before saving them to a TPED file. In this way, globally sorted merging can be fulfilled.

### HBase schema

**Figure 3:**
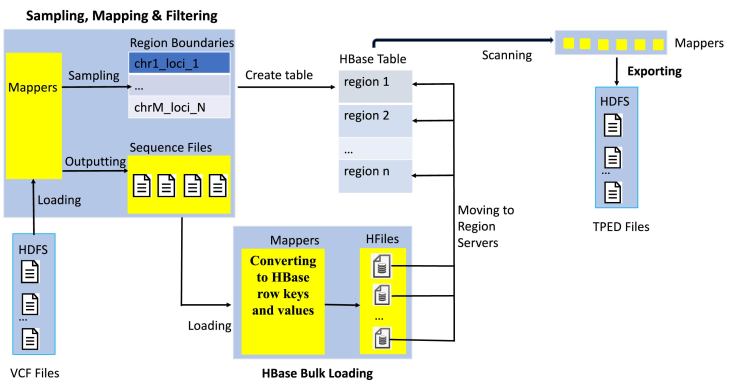
The workflow chart of the HBase schema. The workflow is divided into three phases. The first is a sampling, filtering, and mapping phase. A MapReduce job samples out variants whose genomic positions are used as region boundaries when creating the HBase table. Only qualified records are mapped as key-values and saved as Hadoop sequence files. The second is the HBase bulk loading phase in which a MapReduce job loads and writes records generated from the previous phase, aggregating them into corresponding regional HFiles in the form of HBase's row key and column families. Finished HFiles are moved into HBase data storage folders on region servers. In the third phase, parallel scans were launched over regions of the whole table to retrieve desired records that are subsequently merged and exported to the TPED file.

HBase [8] is a column-oriented database where data are grouped into column families and split horizontally into regions spreading across the cluster. This data storing structure supports efficient sequential reading and writing of large-scale data as well as fast random data accessing. Also, HBase is storage efficient because it can remember null values without saving them on disk. These features make HBase an ideal platform for managing large, sparse data with relatively low latency, which naturally fits the sorted merging case. We use HBase-1.3 as the system for our implementation. As shown in Fig. 3, our optimized HBase schema is divided into three phases as discussed next (refer to Supplementary Fig. S2 for pseudocodes).

#### Sampling phase

The main challenge of HBase is to avoid computational hot spots in the cluster, which can happen when it starts loading a table from a single region hosted by a single node. Therefore, we need to presplit the table into regions of approximately equal size before loading. The sampling phase is introduced to determine reasonable presplitting regional boundaries. The total number of regions is set to half of the number of input files so the size of each region is approximately 1 GB. Meanwhile, mappers of this phase also save qualified records as compressed Hadoop sequence files on HDFS that are used as inputs in the next phase. In addition, filtering and key-value mapping also take place in this phase.

#### Bulk loading phase

Even when the table has been presplit evenly, the hot spot problem of loading sorted inputs can still emerge because sorted records are loaded sequentially and, at any instant, they still access the same region and server. During the bulk loading, the key and value of each record produced from the previous phase is converted into HBase's binary row-key and column-value, respectively, and saved into an HFile, HBase's native storage format. The row-key here is in the form of chromosome-genomic location, and column-value refers to reference allele, individual ID, and genotype. The bulk loading populates each HFile with records falling in the same pair of presplit regional boundaries. Because HFiles are written simultaneously by parallel mappers/reducers, all working nodes are actively involved, and the regional hotspot is thus circumvented. Upon finishing writing, the HBase can readily load HFiles in parallel into the table by simply moving them into local HBase storage folders. This procedure is therefore at least an order of magnitude faster than the normal loading in which data are loaded sequentially via HBase servers’ I/O routines. The order of records in the table is guaranteed because they are internally sorted by writing reducers and HBase's Log-Structured Merge-tree [34]. It is worth mentioning that VCF records are always sparse, thus HBase is very storage efficient.

#### Exporting phase

A scan of a specified genomic window is performed on the table. It involves launching parallel mappers each receiving records from a single HBase region, filling in missing genotypes, concatenating records with the same row-key, and outputting final results into TPED files.

### Spark schema

**Figure 4:**
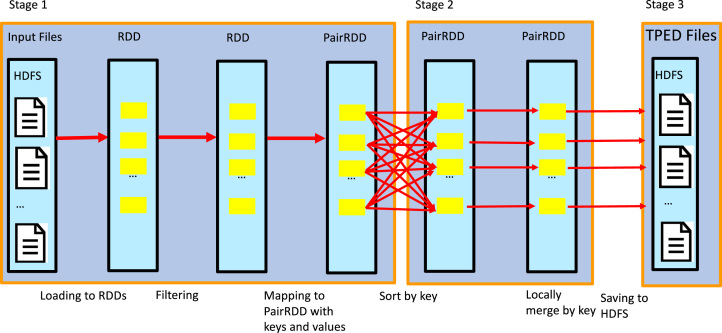
The workflow chart of the Spark schema. The workflow is divided into three stages. In the first stage, VCF records are loaded, filtered, and mapped to pairRDDs with keys of genomic position and values of genotype. The sort-by-key shuffling spans across the first two stages, sorting and grouping together records by keys. Then, grouped records with the same key are locally merged into one record in TPED format. Finally, merged records are exported to the TPED file.

Spark [9] is a distributed engine built upon the ideas of MapReduce and RDD. It can save intermediate results in the form of RDD in memory and perform computations on them. Also, its computations are lazily evaluated, which means the execution plan can be optimized to include as many computational steps as possible. As a result, it is ideal for iterative computations such as sorted merging. We implement our optimized Spark schema on Spark-2.1. It has three stages, which we describe below and present in Fig. 4 (refer to Supplementary Fig. S3 for pseudocodes).

#### RDD preprocessing stage

This stage involves loading raw data as RDDs, filtering, and mapping RDDs to paired-RDDs with keys (chromosome and genomic position) and values (reference allele, sample ID, and genotype). This stage ends with a sorting-by-key action that extends to the next stage.

#### Sorting and merging stage

The sort-by-key shuffling operation repartitions and sorts PairRDD records so records with the same key are aggregated together, which are then merged into the TPED format and converted back to RDD records for outputting. However, Spark's native family of group-by-key functions for merging should not be used here because their default partitioner is hash based and different from the range-based partitioner used by previous sort-by-key function. Consequently, the merged results would be reshuffled into an unsorted status. We therefore optimize the merging to bypass these functions so merging can be performed locally without data reshuffling to ensure both order and high speed.

#### Exporting stage

In this stage, merged RDD records are saved as TPED files on HDFS.

Execution parallelism has an important impact on the performance. To maximize performance, the number of parallel tasks is set to be the number of input files. In this way, data locality is maximized, and each task is assigned a proper amount of work. In addition, unlike using MapReduce or HBase, when performing sorting by keys, no explicit sampling is needed because Spark keeps track of the number of records before determining repartition boundaries.

### Parallel multiway-merge and MPI-based HPC implementations

For most bioinformatics researchers, their daily working environment is still traditional in-house HPC clusters or stand-alone powerful servers (with cores ≥16 and memory ≥200 GB) rather than heterogeneous cloud-based clusters. Therefore, we also implement a parallel multiway-merge program running on a single machine and an MPI-based (mpi4py v3.0) “single program, multiple data” program running on an HPC cluster as benchmarks. The source codes are available at our GitHub website [35] (CloudMerge; RRID:SCR_016051). We chose to implement multiway-merge because many existing bioinformatics tools, including VCFTools and PLINK, adopt it as the underlying algorithm for sorted merging. Multiway-merge is highly efficient on a single machine as it requires only one scan of sorted input files, so it can theoretically run at the speed of disk I/O.

Generally, there are two types of parallelism—data parallelism and task parallelism. The former splits data horizontally into blocks of roughly equal sizes (the size of genomic intervals in our case) before assigning them to all available processes; the latter assigns a roughly equal number of input files to each process. For parallel multiway-merge, we chose data parallelism because the implementation of task parallelism would be the same as the HPC-based implementation running on a single node. Perhaps the most difficult part of data parallelism is uncertainty about the data distribution across all input files, which usually leads to the problem of workload imbalance among processes. If we pre-sample all the input files to estimate the record distribution, then a full scan of the input files is required that will almost certainly take more time than the single-process multiway-merge method. As a compromise, we assume the distributions of SNP locations in all VCF files are uniform and the input files can be split into regions of approximately equal sizes. The total number of regions is set to be the number of concurrent processes, so that each region is specifically handled by a process. To avoid seeking of a process's file reader to its starting offset from the beginning of the file, we take advantage of the Tabix indexer [36], which builds indices on data blocks of the input file and place the reader's pointer directly onto the desired offset. One important aspect of the Tabix indexer is that it requires the input file to be compressed in bgzip format, which is not supported by Hadoop, HBase, or Spark. The compression and decompression of a file in bgzip format can be much faster than in the bz2 format used in our cluster-based schemas, single multiway-merge, and HPC-based implementations, so parallel multiway-merge can run much faster than other methods/schemas when input data size is small.

**Figure 5:**
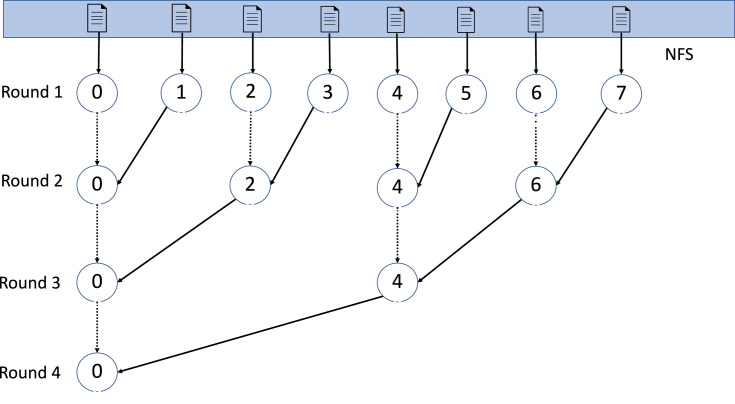
The execution plan of the HPC-based implementation. The execution plan resembles a branched tree. In the first round, each process is assigned an approximately equal number of files to merge locally. In the second round, the even-numbered process retrieves the merged file of its right adjacent process to merge with its local merged file. In the third round, processes with ID numbers that can be fully divided by 4 retrieve the merged file of its right adjacent process in the second round and do the merging. This process continues recursively until all files are merged into a single TPED file (round four).

For the HPC-based implementation, we adopt the task parallelism (Fig. 5) to avoid sampling and workload imbalance. Otherwise, the workflow of HPC-based implementation is the same as that of the MapReduce-based schema with the same operations and the same order: sampling in parallel, dividing the dataset into splits of equal sizes, and assigning the splits to processes to perform the merging. However, this implementation is without data locality offered by HDFS and task coordination offered by YARN and thus has a performance no better than the MapReduce-based schema. Specifically, input files are shared across all nodes in the cluster via a network file system (NFS). In the first round, each core/process fetches roughly the same number of files from the NFS and performs multiway-merging locally. In the following rounds, we adopted a tree-structured execution strategy. In the second round, processes with even ID numbers (process ID starts from 0) retrieve the merged file from its adjacent process to the right, which is then merged with its local merged file. Processes with odd ID numbers are terminated. In the third round, processes with ID numbers divisible by 4 retrieve the merged file from its adjacent process to the right in the second round to merge with its local merged file. This process continues until all the files are merged into a single file for a total of log(*n*) rounds, where *n* is the number of the input files.

### Strong and weak scalabilities

In this study, we quantify scalability by measuring computing efficiency in tests of strong and weak scalabilities. We define efficiency as the average time cost of processing a file per core:

\begin{equation*}
{\rm{Efficiency}} = \left( {{{\rm{T}}_{\rm{b}}}{\rm{*}}{{\rm{C}}_{\rm{b}}}{\rm{/}}{{\rm{N}}_{\rm{b}}}} \right)/\left( {{{\rm{T}}_{\rm{i}}}{\rm{*}}{{\rm{C}}_{\rm{i}}}{\rm{/}}{{\rm{N}}_{\rm{i}}}} \right)
\end{equation*}

where T_b_ is the baseline running time, C_b_ is the baseline number of cores, N_b_ is the baseline number of input files, T_i_ is the current running time, C_i_ is the current number of cores, N_i_ is the current number of input files. We also incorporated the parallel multiway-merge and MPI-based HPC implementations as benchmarks in the tests.

For the strong scalability test, we fix the number of input files at 93 and increase the computing resources up to 16-fold from the baseline. The baseline is a single node (four cores) for all methods/schemas except for the parallel multiway-merge in which only a single core is used because it can only run on a single machine. For the weak scalability test, we increase both computing resources and input data size at the same pace. The ratio is 10 file/core for parallel multiway-merge and 10 file/node for all others.

## Results

We conducted experiments of Apache cluster-based schemas using Amazon's Elastic MapReduce service and experiments of the HPC-based implementation using MIT's StarCluster^TM^ toolkit, which launches an AWS openMP virtual private cluster. Within both infrastructures, we choose EC2 working nodes of m3.xlarge type, which has four high-frequency Intel Xeon E5-2670 v2 (Ivy Bridge) processors and 15 GB ofmemory. We conducted experiments of parallel multiway-merge on a single EC2 r4.8xlarge instance with 32 high-frequency Intel Xeon E5–2686 v4 (Broadwell) processors and 244 GB memory. We used a dataset consisting of 186 VCF files [32] generated from Illumina's BaseSpace software.

### Overall performance analysis of cluster-based schemas

Our primary goal is to explore the scalabilities of the three schemas on input data size and available computing resources, namely, CPUs. To achieve this, in this experiment we adjusted the number of input files from 10 to 186, with an approximate total uncompressed size from 2.5 GB to 40 GB, and used a varying number of working nodes from 3 to 18, namely, 12 to 72 cores.

**Figure 6:**
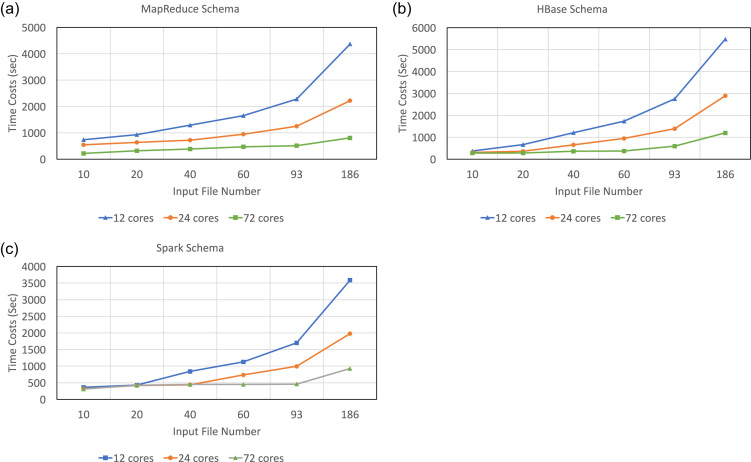
The scalability of Apache cluster-based schemas on input data size. As the number of input files increases from 10 to 186, the time costs of all three schemas with 12, 24, or 72 cores increase at a slower pace than that of the input data size, especially when the number of cores is relatively large. The HBase schema with 12 cores has the largest increase (from 375 to 5479 seconds, ∼14.6 fold).

As Fig. 6 shows, for all three schemas and given a fixed number of cores, the execution time increases at a slower pace than that of the input data size. On the one hand, the increase of execution time is more obvious with fewer cores because each core is fully utilized. As the number of input files increases, so does the number of parallel tasks assigned to each core. For example, given 12 cores, as the number of input files increases from 10 to 186 (18.6 fold), the execution time increases from 739 to 4366 seconds (∼5.9 fold) for the MapReduce schema, from 375 to 5479 seconds (∼14.6 fold) for the HBase schema, and from 361 to 1699 seconds (∼4.7 fold) for the Spark schema. On the other hand, with relatively more cores such as 72, this linear increasing trend is less pronounced because there are more cores than tasks so that all cores are assigned at most one task. We also notice when input data size is small or moderate, the Spark schema does not always show a consistent improvement in terms of execution time with more cores. This is reflected, e.g., in the intersection of curves that occurred between 24 and 72 cores in Fig. 6c. This phenomenon is attributed to the limitation of Spark's internal task assignment policy, which gives rise to the possibility that some nodes are assigned more than one tasks while others remain idle.

### Comparing strong and weak scalabilities between Apache cluster-based schemas and traditional parallel methods

**Figure 7:**
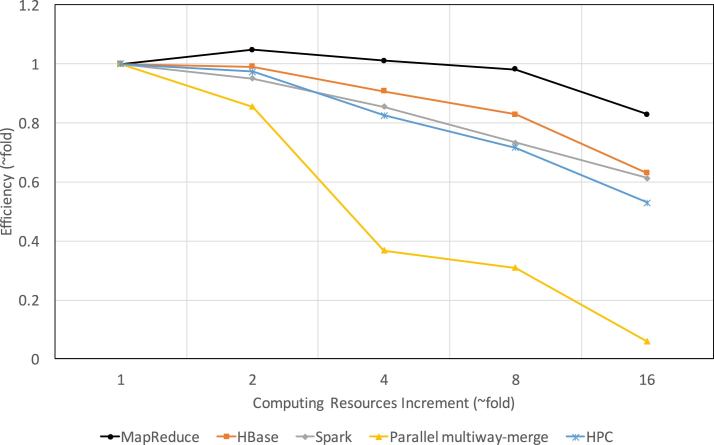
Comparing the strong scalability between traditional parallel/distributed methods and Apache cluster-based schemas. We fix the number of files at 93 and increase the number of nodes/cores. The baseline for the parallel multiway-merge is one single core, while for the others it is one single node (four cores). All methods/schemas show a degraded efficiency as computing resources increase 16 fold from the baseline. Specifically, the efficiency of MapReduce-, HBase-, and Spark-based schemas drops to 0.83, 0.63, and 0.61, respectively, while the efficiency of parallel multiway-merge and HPC-based implementations drops to 0.06 and 0.53, respectively.

Figure 7 shows the results of the strong scalability. In accordance with Amdahl's law [37], all schemas/methods show degraded efficiency with increasing computing nodes/cores. Parallel multiway-merge has the steepest degradation because the more parallel processes, the higher likelihood of workload imbalances among them. In addition, disk I/O reaches saturation as more processes write simultaneously. Furthermore, to achieve data parallelism and improve execution speed, we used Tabix indexer to index data blocks of input files. While reading, each process needs to maintain a full copy of file descriptors, indices, and uncompressed current data blocks of all input files in memory. When both the number of processes and input files are large, great pressure is placed on memory management. For instance, a test with 93 files and 16 processes requires more than 100 GB of memory, which results in a very long memory swap and garbage collection time. In contrast, the MapReduce-based schema has the best efficiency. Surprisingly, its efficiency even improves when the number of cores doubles from the baseline. This is because it has many parallel tasks in its second MapReduce phase; when the core allowance is low, the overheads of repetitive task launching and terminating on a single core become non-negligible. Consequently, as the number of cores starts to increase, the actual proportion of overheads in the total running time decreases, leading to an improved efficiency. Nonetheless, as the number of cores further increases, the unparalleled parts of the schema gradually dominate the total running time, leading to a reduced efficiency eventually.

**Figure 8:**
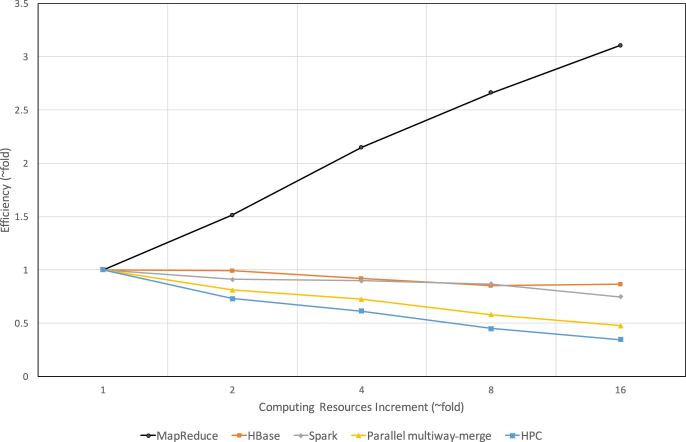
Comparing the weak scalability between traditional parallel/distributed methods and Apache cluster-based schemas. We simultaneously increase the number of cores and input data sizes while fixing the ratio of file/core (parallel multiway-merge) or file/node (all others) at 10. The baseline is the same as in the test of strong scalability. All but the MapReduce-based schema have degraded efficiency, among which the HPC-based implementation has the steepest degradation. Specifically, when computing resource increases 16 fold from the baseline, the efficiency of MapReduce-, HBase-, and Spark-based schemas changes to 3.1, 0.87, and 0.75, respectively, and for parallel multiway-merge and HPC-based implementations, the efficiency reduces to 0.42 and 0.35, respectively.

For the weak scalability test (Fig. 8), following Gustafson's law [38], all methods/schemas show a much better efficiency than in the strong scalability test. Meanwhile, for the same reasons as the strong scalability, the MapReduce-based schema enjoys the best efficiency while the HPC-based implementation has the worst. This is because for the HPC-based implementation, as the number of input files increases, the total number of merging rounds also increases, leading to a significantly reduced efficiency. Finally, all three Apache cluster-based schemas demonstrate significantly better weak scalability than the two traditional parallel methods.

### Anatomic performance analysis of Apache cluster-based schemas

**Figure 9:**
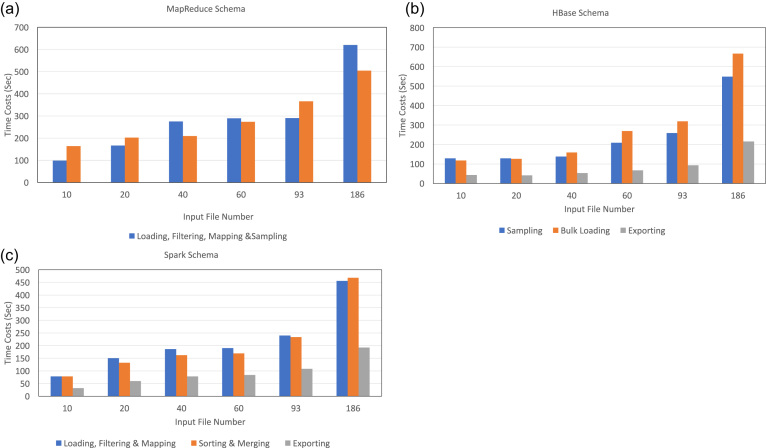
The performance anatomy of cluster-based schemas on increasing input data size. The number of cores in these experiments is fixed at 48. Time costs of all phases of the three schemas have a linear or sublinear correlation with the input data size. (a) MapReduce schema: The two MapReduce phases have a comparable time cost, increasing 6.3- and 3.1-fold, respectively, as the number of input files increases from 10 to 186. (b) HBase schema: The time spent in each phase increases 4.2-, 5.6-,and 5.0-fold, respectively, as the number of input files increases from 10 to 186. The bulk loading and exporting phases together take up more than 80% of total time expense. (c) Spark schema: The time cost increases 5.8-, 6.0-,and 6.0-fold, respectively, for the three stages as the number of input files increases from 10 to 186 files. Like the HBase schema, the first two stages of the Spark schema together account for more than 80% of the total time cost.

Another important goal of our study is to identify potential performance bottlenecks, so we evaluate the execution time of each phase/stage of all three schemas. Figure 9 shows the trends of the anatomic computing time spent on merging an increasing number of VCF files (from 10 to 186) using 48 cores. For the MapReduce schema (Fig. 9a), its two phases account for a comparable proportion of total time and both show a linear or sublinear scalability. The reason that the time cost of the first phase between 40 and 93 input files remains flat is because both runs use two rounds of mappers. As the number of files doubles to 186, four rounds of mappers are required, which results in about a 2-fold increase in the time cost as expected. For the three phases of the HBase schema (Fig. 9b), they are scalable with input data size. Meanwhile, the second phase becomes more dominant with more input files owing to the larger amount of shuffled data during the writing of HFiles. However, we do not consider it as a bottleneck since all tasks of this phase are parallelized with no workload or computational hot spot. Also, we do not observe a super-linear (relative to input data size) increment pattern from the figure. Finally, Fig. 9c shows the time costs of the three stages of the Spark schema. They show a uniform increasing trend with the number of input files. Among them, the second stage takes up a considerable proportion of the total execution time as it has a relatively expensive sort-by-key shuffling operation. Although no data are shuffled in the first stage, its time lapse is close to the second stage. This is because at the end of the first stage, data are sampled to determine the boundaries used by sort-by-key's range partitioner. This operation demands a considerable execution time because it scans all the data and balances them if necessary.

Given that no super-linear increasing trend is observed in running time for all phases/stages of the three schemas, and they generally scale well with the input data size, we conclude that although the performances of these schemas might degrade to some extent when dealing with even larger input data due to overheads such as data transmission over network, we would not expect any significant bottleneck.

### Comparing execution speed between Apache cluster-based schemas and traditional methods

Another intriguing question is: how does the speed of the Apache cluster-based schemas compare to single-machine based and traditional parallel/distributed methods/applications on merging multiple VCF files into a single VCF or TPED file? To answer this question, we choose the widely used VCFTools (v4.2) and a single-process multiway-merge implementation as single-process benchmarks and parallel multiway-merge and HPC-based implementations as parallel/distributed benchmarks, which are the same ones used in the experiments of strong and weak scalabilities described above.

**Figure 10:**
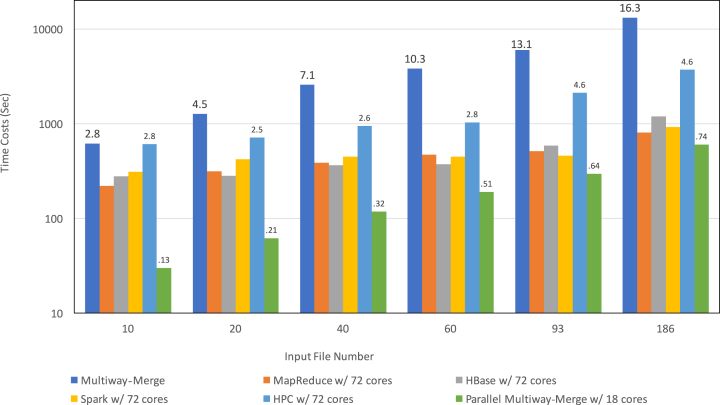
Execution speed comparison among Apache cluster-based schemas and traditional methods. First, we compare the speeds of the three Apache schemas with that of three traditional methods, which are single-process multiway-merge, parallel multiway-merge, and HPC-based implementations. As the number of input files increases from 10 to 186, the speeds of Apache cluster-based schemas improve much more significantly than that of traditional methods. The numbers in the figures indicate the ratio of the time cost of each traditional method to that of the fastest Apache cluster-based schema. Second, we compare the processing speed among the three Apache cluster-based schemas, which are comparable to each other regardless of the input data size. The MapReduce schema performs the best in merging 10 and 186 files; the HBase schema performs the best in merging 20, 40, and 60 files; and the Spark schema performs the best in merging 93 files.

In the first experiment, we merged 40 VCF files into 1 VCF file using VCFTools as the benchmark. As shown in Table 1, VCFTools takes 30,189 seconds, while the fastest Apache cluster-based schema among the three, MapReduce based, takes only 484 seconds using 72 cores, being about 62-fold faster. In the second experiment (Fig. 10), we tested the time costs of merging of multiple VCF files into a single TPED file using single/parallel multiway-merge and HPC-based implementations as benchmarks. The single multiway-merger is run on a node with the hardware configuration (four cores and 15 GB ofmemory) identical to the nodes on which the Apache cluster-based schemas are run. The parallel multiway merger is run on a node with a maximum of 18 simultaneously running processes. The HPC-based implementation is run on an 18-node cluster with the same hardware configuration as the cluster where the Apache cluster-based schemas are run. Initially, with 10 input files, the parallel multiway-merge (∼30 seconds) is much faster than all the other methods; it is about 7.3-fold faster than the fastest Apache cluster-based schema (MapReduce, 221 seconds). On the other hand, the slowest method is the single-process multiway-merger, which takes 620 seconds to finish (about 2.8-fold slower than the MapReduce-based schema). It is worth mentioning that in this test, the parallel multiway-merge is essentially the same as the single-process multiway-merge and that the speed difference (∼378 seconds) between them is the result of a different compression format (bz2 vs bgzip) of the input files as explained above. As we gradually increase the number of input files to 186, the difference in speed between the fastest overall method (parallel multiway merger, 602 seconds) and the fastest Apache cluster-based schema (MapReduce, 809 seconds) decreases to about 1.3-fold, while the difference between the slowest overall method (single multiway-merger, 13,219 seconds) and the MapReduce-based schema increases to 16.3-fold. In addition, all three Apache schemas significantly outperform the HPC-based implementation. As explained in the strong and weak scalabilities section above, we expect that the larger the input data size, the faster the Apache cluster-based schemas would run compared to the other traditional methods.

We also compare the time cost among the three schemas (Fig. 10). They have a comparable speed. More specifically, the MapReduce schema performs best if enough cores are available and the input data size is large; the HBase schema performs best with moderate input data size; the Spark schema performs best if only a limited number of cores are available and the input data size is large. The rationale behind this observation is that when the number of cores is sufficient, the MapReduce-based schema can make the most use of the available computing resources because it runs a constant 25 parallel jobs (one for each of chromosomes 1–22, X, Y, and M [mitochondria]) in its second phase. In contrast, the Spark-based schema has fewer tasks whose numbers equal the number of input files to achieve maximum data-task locality. When the input data size is moderate, the HBase-schema triumphs because of its internal sorting and relatively compact storage format of intermediate data. When the input data size is large and computing resources are relatively limited, the Spark-based schema outperforms the other two owing to its small number of data shuffling (only one), execution plan optimization, and ability to cache intermediate results in memory. We caution, however, that the computing time may fluctuate depending on the distribution of genomic locations in the input files as well as the data loading balance of the HDFS.

## Discussion

In this report, we describe three cluster-based schemas running on the Apache Hadoop (MapReduce), HBase, and Spark platforms for performing sorted merging of variants identified from WGS. We show that all three schemas are scalable on both input data size and computing resources, suggesting large-scale “-omics” data can be merged efficiently given the computing resources readily available in the cloud. Furthermore, the three schemas show better strong and weak scalabilities than traditional single machine-based parallel multiway-merge and cluster-based HPC methods owing to the absence of I/O bottleneck, better workload balance among nodes, and less pressure on memory, as well as data locality and efficient task coordination mechanisms provided by HDFS and YARN. We also show that even with a moderate-sized cluster and input data, all three schemas significantly outperform the broadly used, single-machine based VCFTools, single-process multiway-merge, and HPC-based implementations. Although initially the parallel multiway-merge implementation is much faster than the Apache schemas owing to its advantage of local I/O and light compression of input files, its poor scalability diminishes its initial advantage as the number of concurrent processes and input files increases. Consequently, we expect the Apache cluster-based schemas to eventually outperform the parallel multiway-merge when merging a much larger scale of data using a larger number of cores.

Unlike normal merging, efficient sorted merging of many large tables has always been a difficult problem in the field of data management. Multiway-merge is the most efficient single-machine based method for sorted merging, but its performance is limited by the disk I/O [39]. Sorted merging also places challenges to distributed system-based solutions because neither the efficient hash-based merging nor caching the intermediate table in shared memory is feasible [40]. Although a utility named *total-order-joining* is provided by the Hadoop for addressing this problem, it suffers from both network communication and local disk I/O bottlenecks, and thus is not scalable [27, 41]. In contrast, our schemas divide this problem into different phases/stages of tasks, each conquered in parallel to bypass these bottlenecks and achieve maximum parallelism. Furthermore, in addition to merging sequencing variant data, these schemas can be generalized for other key-based, sorted merging problems that are frequently encountered in genetics and genomics data processing. As an example, they can be slightly modified to merge multiple BED format files such as ChIP-seq peak lists [42] and other genomic regions of interest. Other potentially useful features include the following: unlike traditional sorted merging algorithms that usually require presorted inputs for a better performance, our schemas are free of such a requirement; and our implementations automatically take care of multi-allelic positions, which are frequent in large-scale VCF flies, by retaining the information of all alleles until the merging actually occurs.

Finally, in light of these different features and specialties of these three platforms, each of the three schemas we developed has its own advantages and disadvantages under different application scenarios, as summarized in Table 2. For example, the MapReduce schema is good for a static one-time, nonincremental merging on large-size data provided sufficient cores are available since it has the most parallel jobs, the least overheads, and the most transparent workflow. The HBase schema, supported by data warehousing technologies, fits for an incremental merging since it does not need to re-merge existing results with new ones from scratch only if the incremental merging is performed on the same chromosomes. Also, it provides highly efficient storage and on-line analytical processing on merged results. The Spark schema is ideal for merging large-scale data with relatively limited computing resources because it has the least data shuffling and keeps intermediate results in memory. A bonus brought by Spark is that subsequent statistical analyses can be carried out directly on the merged results using its rich set of parallel statistical utilities.

**Table 1:** Performance comparisons between VCTools and Apache cluster-based schemas

	VCFTools	MapReduce	HBase	Spark
Time cost (seconds)	30,189	484	577	596
Fold (faster)	-	62.4	52.3	50.7

**Table 2:** Pros and cons of MapReduce, HBase, and Spark schemas

Schema	*Pros*	*Cons*
**MapReduce**	• Good for large input data size and sufficient computing resources	• Merging is not incremental
	• Simple architecture and least overheads given sufficient computing resources	• Large overhead when computing resources are limited
	• Best parallelism	
	• Good for one-time merging	
	• Performance is stable	
**HBase**	• Good for intermediate input data size (≥ 20 and ≤ 100 VCF files)	• Users must determine region number in advance
	• Supports incremental merging	• Has most local I/O
	• Supports on-line analytical processing	• Complex performance tuning
	• Best storage efficiency	
**Spark**	• Good for large input data size (>100 VCF files) and relative limited computing resources	• Possibly weakened data locality during loading
	• Keeps intermediate results in memory and least local I/O	• Slight unstable performance when computing resources exceeds needs of input data size
	• Good for subsequent statistical analysis on merged results	• Actual execution plan is not transparent
		• Complex performance tuning

## Availability and requirements

Project name: CloudMerge

Project home page: https://github.com/xsun28/CloudMerge

Operating system(s): Linux

Programming language: Java, Python

Other requirements: Java 1.7 or higher, Python 2.7 or 3.6, Hadoop-2.7, HBase-1.3, Spark-2.1, StarCluster 0.95, MPI for Python 3.0.0

License: Apache License 2.0

## Availability of supporting data

The source codes for the project are available in GitHub. The 186 individual VCF files used in our experiments are modified from the original VCF files obtained from WGS conducted by the Consortium on Asthma among the African-Ancestry Population in the Americas (CAAPA) [32]. To conceal the potential individual identifiable genotype information from the public, we encrypt the authentic genomic location of the original 93 VCF files to generate a new batch of encrypted VCF files for test purposes. Please refer to the Data formats and operations section for details. These supporting data and a snapshot of project codes are available at the *GigaScience* database, GigaDB [43]. Via GigaDB, we also provide sample results of merging 93VCF files into either one VCF or one TPED file using our Apache cluster-based schemas.

## Additional file

**Figure S1**. Pseudocodes of the MapReduce schema.

**Figure S2**. Pseudocodes of the HBase schema.

**Figure S3**. Pseudocodes of the Spark schema.

## Abbreviations

AWS: Amazon Web Service; CAAPA: Consortium on Asthma among African-ancestry Population in the Americas; CPU: central processing unit; GWAS: genome-wide association studies; HDFS: Hadoop distributed Systems; HPC: high-performance computing; I/O: Input/Output; MPI: message passing interface; NFS: network file system; NGS: next generation sequencing; RDD: resilient distributed dataset; SNP: single-nucleotide polymorphism; VCF: variant call format; WGS: whole-genome sequencing.

## Ethics approval and consent to participate

Ethics approval for the CAAPA program was provided by the Johns Hopkins University Institutional Review Board (IRB) following commencement of the study in 2011 (IRB00045892, CAAPA ) and included study team members from each CAAPA site, including Emory University (site PI, Zhaohui Qin). Access to the raw data as CAAPA team members is granted according to the guideline of the IRB-approved study. Informed consent has been obtained from all study participants of CAAPA.

## Competing interests

The authors declare that they have no competing interests.

## Funding

This study was supported by grants from the National Heart, Lung, and Blood Institute (R01HL104608, R01HL117004, R01HL128439, R01HL135156, X01HL134589); National Institute of Environmental Health Sciences (R01ES015794, R21ES24844); National Institute on Minority Health and Health Disparities (P60MD006902, R01MD010443, RL5GM118984); National Institute of Neurological Disorders and Stroke (R01NS051630, P01NS097206, U54NS091859); National Science Foundation (ACI 1443054, IIS 1350885); Tobacco-Related Disease Research Program (24RT-0025). The Genes-Environments and Admixture in Latino Americans (GALA II) Study; the Study of African Americans, Asthma, Genes and Environments (SAGE) Study; and E.G.B. are supported by the Sandler Family Foundation, the American Asthma Foundation, the RWJF Amos Medical Faculty Development Program, and the Harry Wm. and Diana V. Hind Distinguished Professor in Pharmaceutical Sciences II.

## Authors’ Contributions

J.G. introduced the problem. X.S. and F.W. initiated the project. X.S. designed and implemented the CloudMerge project. X.S. drafted the manuscript. X.S., J.P., F.W., and Z.Q. revised the manuscript.

K.C.B. conceived the initial consortium design, acquired biospecimens for Next Generation Sequencing (NGS), and facilitated generation of NGS data. K.C.B., R.A.M., I.R., and T.H.B. conceived initial experiments and interpreted NGS data. E.G.B. and C.E. acquired biospecimens for NGS and facilitated generation of NGS data.

## CAAPA Consortium Members and Their Affiliations

^1,2^Kathleen C. Barnes, PhD, Terri H. Beaty, PhD^2^, Meher Preethi Boorgula MS^1^, Monica Campbell, BS^1^, Sameer Chavan, MS^1^, Jean G. Ford, MD^2,3^, Cassandra Foster, CCRP^1^, Li Gao, MD, PhD^1^, Nadia N. Hansel, MD, MPH^1^, Edward Horowitz, BA^1^, Lili Huang, MPH^1^, Rasika Ann Mathias, ScD^1,2^, Romina Ortiz, MA^1^, Joseph Potee, MS^1^, Nicholas Rafaels, MS^1^, Ingo Ruczin-ski, PhD^4^, Alan F. Scott, PhD^1^, Margaret A. Taub, PhD^4^, Candelaria Ver-gara, PhD^1^, Jingjing Gao, PhD^5^, Yijuan Hu, PhD^6^, Henry Richard Johnston, PhD^6^, Zhaohui S. Qin, PhD^6^, Albert M. Levin, PhD^7^, Badri Padhukasahas-ram, PhD^8^, L. Keoki Williams, MD, MPH^8,9^, Georgia M. Dunston, PhD^10,11^, Mezbah U. Faruque, MD, PhD^11^, Eimear E. Kenny, PhD^12,13^, Kimberly Gi-etzen, PhD^14^, Mark Hansen, PhD^14^, Rob Genuario, PhD^14^, Dave Bullis, MBA^14^, Cindy Lawley, PhD^14^, Aniket Deshpande, MS^15^, Wendy E. Grus, PhD^15^, Devin P. Locke, PhD^15^, Marilyn G. Foreman, MD^16^, Pedro C. Avila, MD^17^, Leslie Grammer, MD^17^, Kwang-Youn A. Kim, PhD^18^, Rajesh Kumar, MD^19,20^, Robert Schleimer, PhD^21^, Carlos Bustamante, PhD^12^, Francisco M. De La Vega, DS^12^, Chris R. Gignoux, MS^12^, Suyash S. Shringarpure, PhD^12^, Shaila Musharo, MS^12^, Genevieve Wojcik, PhD^12^, Esteban G. Burchard, MD, MPH^22,23^, Celeste Eng, BS^23^, Pierre-Antoine Gourraud, PhD^24^, Ryan D. Hernandez, PhD^22,25,26^, Antoine Lizee, PhD^24^, Maria Pino-Yanes, PhD^23,27^, Dara G. Torgerson, PhD^23^, Zachary A. Szpiech, PhD^22^, Raul Torres, BS^28^, Dan L. Nicolae, PhD^29,30^, Carole Ober, PhD^31^, Christopher O Olopade, MD, MPH^32^, Olufunmilayo Olopade, MD^29^, Oluwafemi Oluwole, MSc^29^, Ganiyu Arinola, PhD^33^, Timothy D. O'Connor, PhD^34,35,36^, Wei Song, PhD^34,35,36^, Goncalo Abecasis, DPhil^37^, Adolfo Correa, MD, MPH, PhD^38^, Solomon Musani, PhD^38^, James G. Wilson, MD^39^, Leslie A. Lange, PhD^40^, Joshua Akey, PhD^41^, Michael Bamshad, MD^42^, Jessica Chong, PhD^42^, Wenqing Fu, PhD^41^, Deborah Nickerson, PhD^41^, Alexander Reiner, MD, MSc^43^, Tina Hartert, MD, MPH^44^, Lorraine B. Ware, MD^44,45^, Eugene Bleecker, MD^46^, Deborah Meyers, PhD^46^, Victor E. Ortega, MD^46^, Pissamai Maul, BSc, RN^47^, Trevor Maul, RN^47^, Harold Watson, MD^48,49^, Maria Ilma Araujo, MD, PhD^50^, Ricardo Riccio Oliveira, PhD^51^, Luis Caraballo, MD, PhD^52^, Javier Marrugo, MD^53^, Beatriz Martinez, MSc^52^, Catherine Meza, LB^52^, Gerardo Ayestas^54^, Edwin Francisco Herrera-Paz, MD, MSc^55,56,57^, Pamela Landaverde-Torres^55^, Said Omar Leiva Erazo^55^, Rosella Martinez, BSc^55^, varo Mayorga, MD^56^, Luis F. Mayorga, MD^55^, Delmy-Aracely Mejia-Mejia, MD^56,57^, Hector Ramos^55^, Allan Saenz^54^, Gloria Varela^54^, Olga Marina Vasquez^57^, Trevor Ferguson, MBBS, DM, MSc^58^, Jennifer Knight-Madden, MBBS, PhD^58^, Maureen Samms-Vaughan, MBBS, DM, Ph^59^, Rainford J. Wilks, MBBS, DM, MSc^58^, Akim Adegnika, MD, PhD^60,61,62^, Ulysse Ateba-Ngoa, MD^60,61,62^, Maria Yazdanbakhsh, PhD^62^

^1^Department of Medicine, Johns Hopkins University, Baltimore, MD,

^2^Department of Epidemiology, Bloomberg School of Public Health, JHU, Baltimore, MD,

^3^Department of Medicine, The Brooklyn Hospital Center, Brooklyn, NY,

^4^Department of Biostatistics, Bloomberg School of Public Health, JHU, Baltimore, MD,

^5^Data and Statistical Sciences, AbbVie, North Chicago, IL,

^6^Department of Biostatistics and Bioinformatics, Emory University, Atlanta, GA,

^7^Department of Public Health Sciences, Henry Ford Health System, Detroit, MI,

^8^Center for Health Policy & Health Services Research, Henry Ford Health System, Detroit, MI,

^9^Department of Internal Medicine, Henry Ford Health System, Detroit, MI,^10^Department of Microbiology, Howard University College of Medicine, Washington, DC,

^11^National Human Genome Center, Howard University College of Medicine, Washington, DC,

^12^Department of Genetics, Stanford University School of Medicine, Stanford, CA,

^13^Department of Genetics and Genomics, Icahn School of Medicine at Mount Sinai, New York,

^14^Illumina, Inc., San Diego, CA,

^15^Knome Inc., Cambridge, MA,

^16^Pulmonary and Critical Care Medicine, Morehouse School of Medicine, Atlanta, GA,

^17^Department of Medicine, Northwestern University, Chicago, IL,

^18^Department of Preventive Medicine, Northwestern University, Chicago, IL,

^19^Department of Pediatrics, Northwestern University, Chicago, IL,

^20^The Ann & Robert H. Lurie Children's Hospital of Chicago, Chicago, IL,

^21^Department of Medicine, Northwestern Feinberg School of Medicine, Chicago, IL,

^22^Department of Bioengineering and Therapeutic Sciences, University of California, San Francisco, San Francisco, CA,

^23^Department of Medicine, University of California, San Francisco, San Francisco, CA,

^24^Department of Neurology, University of California, San Francisco, San Francisco, CA,

^25^Institute for Human Genetics, Institute for Human Genetics, University of California, San Francisco, San Francisco, CA,

^26^California Institute for Quantitative Biosciences, University of California, San Francisco, San Francisco, CA,

^27^CIBER de Enfermedades Respiratorias, Instituto de Salud Carlos III, Madrid,

^28^Biomedical Sciences Graduate Program, University of California, San Francisco, San Francisco, CA,

^29^Department of Medicine, University of Chicago, Chicago, IL,

^30^Department of Statistics, University of Chicago, Chicago, IL,

^31^Department of Human Genetics, University of Chicago, Chicago, IL,

^32^Department of Medicine and Center for Global Health, University of Chicago, Chicago, IL,

^33^Department of Chemical Pathology, University of Ibadan, Ibadan, Nigeria,

^34^Institute for Genome Sciences, University of Maryland School of Medicine, Baltimore, MD,

^35^Program in Personalized and Genomic Medicine, University of Maryland School of Medicine, Baltimore, MD,

^36^Department of Medicine, University of Maryland School of Medicine, Baltimore, MD,

^37^Department of Biostatistics, SPH II, University of Michigan, Ann Arbor, MI,

^38^Department of Medicine, University of Mississippi Medical Center, Jackson, MS,

^39^Department of Physiology and Biophysics, University of Mississippi Medical Center, Jackson, MS,

^40^Department of Genetics, University of North Carolina, Chapel Hill, NC,

^41^Department of Genomic Sciences, University of Washington, Seattle, WA,

^42^Department of Pediatrics, University of Washington, Seattle, WA,

^43^University of Washington, Seattle, WA,

^44^Department of Medicine, Vanderbilt University, Nashville, TN,

^45^Department of Pathology, Microbiology and Immunology, Vanderbilt University, Nashville, TN,

^46^Center for Human Genomics and Personalized Medicine, Wake Forest School of Medicine, Winston-Salem, NC,

^47^Genetics and Epidemiology of Asthma in Barbados, The University of the West Indies,

^48^Faculty of Medical Sciences Cave Hill Campus, The University of the West Indies,

^49^Queen Elizabeth Hospital, Queen Elizabeth Hospital, The University of the West Indies,

^50^Immunology Service, Universidade Federal da Bahia, Salvador, BA,

^51^Laboratrio de Patologia Experimental, Centro de Pesquisas Gonalo Moniz, Salvador, BA,

^52^Institute for Immunological Research, Universidad de Cartagena, Cartagena,

^53^Instituto de Investigaciones Immunologicas, Universidad de Cartagena, Cartagena,

^54^Faculty of Medicine, Universidad Nacional Autonoma de Honduras en el Valle de Sula, San Pedro Sula,

^55^Facultad de Medicina, Universidad Catolica de Honduras, San Pedro Sula,

^56^Centro de Neumologia y Alergias, San Pedro Sula,

^57^Faculty of Medicine, Centro Medico de la Familia, San Pedro Sula,

^58^Tropical Medicine Research Institute, The University of the West Indies,

^59^Department of Child Health, The University of the West Indies,

^60^Centre de Recherches Mdicales de Lambarn,

^61^Institut fr Tropenmedizin, Universitt Tbingen,

^62^Department of Parasitology, Leiden University Medical Center, Netherlands.

## Supplementary Material

GIGA-D-17-00267_Original_Submission.pdf

GIGA-D-17-00267_Revision_1.pdf

GIGA-D-17-00267_Revision_2.pdf

Response_to_Reviewer_Comments_Original_Submission.pdf

Response_to_Reviewer_Comments_Revision_1.pdf

Reviewer_1_Report_(Original_Submission) -- Bernie Pope 11/5/2017 Reviewed

Reviewer_1_Report_(Revision_1) -- Bernie Pope 2/26/2018 Reviewed

Reviewer_2_Report_(Original_Submission) -- Ivan Merelli 11/19/2017 Reviewed

Reviewer_2_Report_(Revision_1) -- Ivan Merelli 2/15/2018 Reviewed

Reviewer_3_Report_(Original_Submission) -- Tomasz Gambin 12/11/2017 Reviewed

Supplemental Files
