# Supplementary material for: Optimized distributed systems achieve significant performance improvement on sorted merging of massive VCF files
Source: Gigascience. 2018 May 10;7(6):giy052. doi: 10.1093/gigascience/giy052 (PMC6007233; doi:10.1093/gigascience/giy052)
Supplement: GIGA-D-17-00267_Original_Submission.pdf [file giy052_giga-d-17-00267_original_submission.pdf]

GigaScience

Optimized Distributed Systems Achieve Significant Performance Improvement on Sorted Merging of Massive Omics Data

--Manuscript Draft--

**Manuscript Number:**	GIGA-D-17-00267
**Full Title:**	Optimized Distributed Systems Achieve Significant Performance Improvement on Sorted Merging of Massive Omics Data
**Article Type:**	Technical Note
**Funding Information:**	
**Abstract:**	Background: Sorted merging of genomic data is a common data operation necessary in whole genome sequencing studies. It involves sorting and merging genomic data from different subjects by genomic locations. With the rapid increase of high throughput experimental data, the computational burden makes traditional methods designed for a single computer no longer feasible to this problem. The newly emerged distributed systems have the potential to offer a much needed boost in performance. However, carefully designed optimization schemas are required to take advantage of the increased computing power while overcoming bottlenecks to achieve maximum performance.<br><br>Findings: In this study, we custom design optimized schemas for three Apache big data platforms, MapReduce, HBase and Spark, to perform sorted merging of massive genome-wide data. These schemas all adopt the divide-and-conquer strategy to split the merging job into sequential phases/stages consisting of subtasks which are conquered in an ordered, parallel and bottleneck-free way. In two illustrating examples, we test the performance of our schemas on merging multiple Variant Call Format (VCF) files into either a TPED or a VCF file, which are benchmarked with the traditional multiway-merge method and the popular VCFTools.<br><br>Conclusions: Our experiments suggest that all three schemas deliver a significant performance improvement over existing methods. More importantly, they all show good scalability on input size and computing resources. Therefore our findings provide generalized scalable schemas for performing sorted merging on genetics and genomics data using these Apache distributed systems.
**Corresponding Author:**	Zhaohui Qin<br><br>UNITED STATES
**Corresponding Author Secondary Information:**	
**Corresponding Author's Institution:**	
**Corresponding Author's Secondary Institution:**	
**First Author:**	Xiaobo Sun
**First Author Secondary Information:**	
**Order of Authors:**	Xiaobo Sun
	Zhaohui Qin
	Fusheng Wang
	Jingjing Gao
	Peng Jin
**Order of Authors Secondary Information:**	
**Opposed Reviewers:**	

Powered by Editorial Manager® and ProduXion Manager® from Aries Systems Corporation

Additional Information:	
**Question**	**Response**
Are you submitting this manuscript to a special series or article collection?	No
**Experimental design and statistics**<br><br>Full details of the experimental design and statistical methods used should be given in the Methods section, as detailed in our Minimum Standards Reporting Checklist. Information essential to interpreting the data presented should be made available in the figure legends.<br><br>Have you included all the information requested in your manuscript?	Yes
**Resources**<br><br>A description of all resources used, including antibodies, cell lines, animals and software tools, with enough information to allow them to be uniquely identified, should be included in the Methods section. Authors are strongly encouraged to cite Research Resource Identifiers (RRIDs) for antibodies, model organisms and tools, where possible.<br><br>Have you included the information requested as detailed in our Minimum Standards Reporting Checklist?	Yes
**Availability of data and materials**<br><br>All datasets and code on which the conclusions of the paper rely must be either included in your submission or deposited in publicly available repositories (where available and ethically appropriate), referencing such data using a unique identifier in the references and in the "Availability of Data and Materials" section of your manuscript.<br><br>Have you have met the above requirement as detailed in our Minimum Standards Reporting Checklist?	Yes

Powered by Editorial Manager® and ProduXion Manager® from Aries Systems Corporation

Manuscript

Click here to download Manuscript submission manuscript.docx

Optimized Distributed Systems Achieve Significant Performance Improvement on Sorted Merging of Massive Omics Data

Xiaobo Sun[1], Jingjing Gao[2], Peng Jin[3], Fusheng Wang[4*], Zhaohui Qin[2,5*]

[1]Department of Computer Sciences, Emory University, Atlanta, GA 30322, USA.

[2]Department of Medical Informatics, Emory University School of medicine, Atlanta, GA 30322, USA.

[3]Department of Human Genetics, Emory University School of Medicine, Atlanta, GA 30322, USA.

[4]Department of Biomedical Informatics, Stony Brook University, Stony Brook, NY 11794, USA.

[5]Department of Biostatistics, Emory University, Atlanta, GA 30322, USA.

X.S. Email: xsun28@emory.edu

J.G. Email: jingjing.gao@abbvie.com

P.J. Email: peng.jin@emory.edu

F.W. Email: fusheng.wang@stonybrook.edu

Z.Q. Email: zhaohui.qin@emory.edu

*Correspondence: zhaohui.qin@emory.edu, fusheng.wang@stonybrook.edu

Abstract

Background: Sorted merging of genomic data is a common data operation necessary in whole genome sequencing studies. It involves sorting and merging genomic data from different subjects by genomic locations. With the rapid increase of high throughput experimental data, the computational burden makes traditional methods designed for a single computer no longer feasible to this problem. The newly emerged distributed systems have the potential to offer a much needed boost in performance. However, carefully designed optimization schemas are required to take advantage of the increased computing power while overcoming bottlenecks to achieve maximum performance.

Findings: In this study, we custom design optimized schemas for three Apache big data platforms, MapReduce, HBase and Spark, to perform sorted merging of massive genome-wide data. These schemas all adopt the divide-and-conquer strategy to split the merging job into sequential phases/stages consisting of subtasks which are conquered in an ordered, parallel and bottleneck-free way. In two illustrating examples, we test the performance of our schemas on merging multiple Variant Call Format (VCF) files into either a TPED or a VCF file, which are benchmarked with the traditional multiway-merge method and the popular VCFTools.

Conclusions: Our experiments suggest that all three schemas deliver a significant performance improvement over existing methods. More importantly, they all show good scalability on input size and computing resources. Therefore our findings provide generalized scalable schemas for performing sorted merging on genetics and genomics data using these Apache distributed systems.

Keywords: Sorted merging, whole genome sequencing, MapReduce, Hadoop, HBase, Spark.

Findings

Introduction

With rapid development of high-throughput biotechnologies, genetics studies have entered the Big Data era. Studies like Genome Wide Association Studies (GWASs), Whole Genome Sequencing (WGS) and whole exome sequencing (WES) studies have produced a massive amount of data. The ability to efficiently process such massive data becomes increasingly important in a successful large scale genetics study [1, 2]. Traditional single machine based methods are no longer feasible to process such big data due to the prohibitive computation time and I/O bottleneck. It becomes increasingly attractive for investigators to take advantage of the powerful distributed computing resources or the cloud to perform data processing and analyses [3]. Apache

Foundation has been a leading force in this endeavor and has developed multiple platforms and systems including Hadoop [4, 5], HBase [6] and Spark [7]. All these three Apache platforms have gained increasing popularity in recent years, and have been endorsed and supported by major vendors such as Amazon Web Services (AWS).

In bioinformatics, researchers have recently started to embrace distributed systems to process large amount of high throughput omics data. For example, both the CloudBurst [8] and Crossbow software [9] takes advantage of the Hadoop framework to accelerate sequencing read mapping and SNP calling. The Collaborative Genomic Data Model (CGDM) [10] uses HBase to boost the querying speed for the main classes of queries on genomic databases. The ADAM project [1], built on the Spark platform, adapts the Sequence/Binary Alignment/Map (SAM/BAM) formats to distributed computing environments. Industry cloud computing vendors such as Amazon [11] and Google [12] are also beginning to provide specialized environments to ease genomics data processing in the cloud.

Despite their potentials, applications of Apache big data platform in genetics and genomics studies are still relatively limited. We believe there are plenty of opportunities as data becomes larger and more complex. One particular example is sorted merging, which is a ubiquitous operation in processing genetics and genomics data. As an example, in WGS, variants identified from individuals are often called and stored in separate VCF files, subsequently these VCF files need to be merged (into a VCF or TPED file) as required by downstream analyses such as PLINK [13] and BlueSNP [14, 15]. Either a VCF or TPED file requires data to be sorted by genomic location, thus these tasks are equivalent to the well-known sorted full-outer-joining problem [16, 17]. Currently, they are handled by software such as VCFTools [18] and PLINK. These utilities become very cumbersome even in the face of a moderate scale of genomic data. The main reason is that most of these tools adopt the multiway-merge-like method [19] with a priority queue as the underlying data structure to ensure the output order. A key deficiency of such method is that it can only have one consumer to access items from the queue, which literally makes it single-threaded, even if there can be parallel producers that put items into the queue. Therefore, these single-machine based tools are inefficient and time-consuming when handling large datasets.

In this study, we use the case of the sorted-merging of multiple VCF files to a single file to demonstrate the benefits of using distributed platforms. However, simply running sorted merging on a distributed system runs into problems of bottlenecks, hotspots and unordered results commonly seen in parallel computations. Rather, we believe working schemas custom designed for each specific distributed platform are required to unleash the full potential of these distributed systems. We propose and implement three schemas running on Hadoop, Spark and HBase respectively to overcome the limitations of both single-machine and simple distributed system based methods. We choose these three platforms because they are representative cloud distributed systems providing data partitioning based parallelism with distributed storage, data partitioning based parallelism with in-memory based processing, and high dimensional table like distributed storage, respectively. Hadoop [4] is the open source implementation of MapReduce [5] based parallel key-value processing technique, and has the advantage of transparency and simplicity. HBase [6] is a data warehousing platform which adopts Google's BigTable data storing structure [20] to achieve high efficiency in storing and reading/writing large scale of sparse data. Spark [7]

introduces the concept of Resilient Distributed Dataset (RDD) and Directed Acyclic Graph (DAG) execution to parallel key-value processing, thus enabling fast, robust and repetitive in-memory data manipulations. Specifically, our schemas involve dividing the job into multiple phases corresponding to tasks of loading, mapping, filtering, sampling, partitioning, shuffling, merging and outputting. Within each phase, data and tasks are evenly distributed across the cluster, enabling processing large scale of data in a parallel and scalable manner, which in turn significantly boosts performance.

Methods

Overview

Compared to using the multiway-merge method [19] or a relational database based approach, the benefits of using the three Apache distributed platforms to perform sorted merging are three-fold. First, with representation of genomic locations as keys and genotypes as values, it is readily transformed into the key-value model on which all three platforms offer a rich set of parallel operations. Second, data in VCF files are semi-structured. Semi-structured data ideally fit for all

three platforms which allow defining the schema during data loading, avoiding the preprocessing of raw data into a rigid schema as in a relational database. Third, the merged results are outputted onto a distributed file system such as HDFS and Amazon S3 which can be directly used for subsequent cluster-based GWAS or WGS analytical tools such as BlueSNP.

Despite these advantages, simply performing sorted merging on distributed systems will not deliver expected results for the following reasons. First, it can lead to globally unsorted results. Hash-based shuffling of input data is the default mechanism for distributing data to parallel working units in the system. However, shuffling will lead to globally unsorted results. Second, bottleneck and hotspot can happen during the processing in the cluster. Bypassing the hashing based shuffling can lead to unbalanced workload across the cluster, result in straggling computing units which become the bottlenecks for response time. In addition, for parallel loading of presorted data into HBase, data being loaded from all the loading tasks will hit the same node simultaneously while other machines are idling, leading to an I/O hotspot. Third, sampling costs could become prohibitive. Although Hadoop provides a native utility named *total-order-merging* [16] to achieve both workload balance and global order, it involves transferring to and sampling

all the data onto a single node. The communication cost over the network and disk I/O can be prohibitive when data size is very large. In the following sections, we will illustrate how our custom designed schema are able to overcome these limitations in detail.

Data Formats and Operations

In a typical WGS, data analysis often starts from individual genotype files in VCF format [21]. A VCF file contains data arranged into a table consisting of eight mandatory fields including chromosome (CHROM), the genomic coordinate of the start of the variant (POS), the reference allele (REF), a comma separated list of alternate alleles (ALT), among others. In our experiments, we use a dataset consisting of the VCF files of 93 individuals [22] generated from Illumina's BaseSpace software (Left tables in Figure 1). Each file has around 4-5 million rows, each representing one of the individual's genomic variants, with a size of about 300 megabytes. In an attempt to protect the privacy of the study subjects, we apply the following strategy to conceal their real genetic variant information contained in the VCF files: we first transform each original genomic location by multiplying it with an undisclosed constant real number, taking the floor integer of the result, and then add another undisclosed constant integer number.

It is common that multiple VCF files need to be merged into a single TPED file for analysis tools such as PLINK. A TPED file resembles a big table, aggregating genotypes of all individuals under investigation by genomic location (Right table in Figure 1). The merging follows several rules. First, records having an unqualified filter value are discarded. Second, genotypes in VCF files are stored as binary codes where 0 stands for reference allele while 1 stands for mutant allele. Binary codes must be translated into corresponding types of nucleotides in the TPED file. Third, all individuals need to have a genotype for genomic locations that appears in at least one VCF file. The default genotype for missing values is homozygous reference alleles.

MapReduce Schema

MapReduce [5] is a parallel computing model based on a *split-apply-combine* strategy for data analysis, in which data are mapped to key-values for splitting (mapping), shuffling and combining (reducing) for final results. We use Apache Hadoop-2.7 as the system for our implementation. Our optimized schema consists of two MapReduce phases, as shown in Figure 2.

First MapReduce phase. Raw data are loaded from HDFS into parallel mappers to perform the following tasks: First, unqualified data are filtered out and qualified ones are mapped to key-value pairs. The mapper output key is a genomic location and output value is genotype and individual ID. Second, Key-value pairs are grouped together by their chromosome and temporarily saved as compressed Hadoop sequence files [23] for faster I/O in the second MapReduce phase. With this grouping, we can merge records from selected chromosomes of interests rather than from all of them. Meantime, these records are sampled to explore their key distribution profile along the chromosomes for determining boundaries in between each pair of which there is approximately an equal number of records. Specifically, the genomic locations of sampled-out records for each chromosome are used as boundaries to split the chromosome into disjoint segments. Because records falling in the same segment will be assigned to the same reducer in the later phase, boundaries calculated in this way ensure that the workload of each reducer is balanced. There are two rounds of samplings. The first one happens in each mapper with a pre-specified sampling rate, which in our case is set to 0.0001. To separate sampled records by chromosome they are distributed to different reducers in this phase based on their chromosomes, where they are sampled again with a rate equal to the reciprocal of input file number. This second sampling limits the

number of final sampled records even in the face of a large number of input files. Because the number of reducers instantiated in the second phase is decided by the number of sampled records, we can therefore avoid launching unnecessary reducers thus reducing task overhead.

Second MapReduce phase. In this phase, multiple parallel MapReduce jobs are created, and each job specifically handles all records of a single chromosome outputted as sequence files in the first phase. Within each job, a partitioner shuffles records to the appropriate reducer by referring to the boundaries from the previous phase, so that records falling in between the same pair of boundaries are aggregated together. Finally, each reducer sorts and merges aggregated records by genomic location before outputting them to a TPED file. In this way, globally sorted merging can be fulfilled.

HBase Schema

HBase [6] is a column-oriented database where data are grouped into column families and split horizontally into regions spreading across the cluster. With this data storing structure, it supports efficient sequential reading and writing of large-scale data as well as fast random data accessing.

Also, HBase is storage efficient because it can remember null values without saving them on disk. These features make HBase an ideal platform for managing large, sparse data with relatively low latency which naturally fits the sorted merging case. We use the HBase-1.3 as the system for our implementation. As shown in Figure 3, our optimized HBase schema is divided into three phases as discussed next.

1) Sampling phase

The main challenge of HBase lies in that it is not uncommon to find that one server of the cluster becomes a computational hotspot. This can happen when it starts loading a table from a single region hosted by a single node. Therefore, we need to presplit the table into regions of approximately equal size before loading. The sampling phase is introduced to determine reasonable presplitting regional boundaries. The total region number is set to be half of the number of input files so that the size of each region is approximately 1GB. Meanwhile, mappers of this phase also output qualified records as compressed Hadoop sequence files on HDFS which are used as inputs in the next phase. In addition, filtering and key-value mapping also take place in this phase.

2) Bulk loading phase

Even when the table has been presplit evenly, the hotspot problem of loading sorted inputs is not yet fully solved because sorted records are loaded sequentially and all the records being loaded at any instant still hit the same region and server. This necessitates the adding of this phase. During the bulk loading, the key and value of each record outputted from previous phase is converted into HBase’s binary row-key and column-value respectively, and saved as HFile, HBase’s native storage format. The row-key here is in the form of chromosome-genomic location, and column-value refers to reference allele, individual ID and genotype. The bulk loading populates each HFile with records falling in the same pair of presplit regional boundaries. Because HFiles are written simultaneously by parallel mappers/reducers, all working nodes are actively involved and the regional hotspot is thus circumvented. Upon finishing writings, the HBase can readily load HFiles in parallel into the table by simply moving them into local storage folders. This procedure is therefore at least a magnitude faster than the normal loading. The order of records in the table is guaranteed because they are internally sorted by writing reducers and HBase’s Log-Structured Merge-tree [24]. It is noteworthy to mention that VCF records are sparse, thus HBase is very

storage-efficient.

3) Exporting Phase

A scan is performed on the table. It involves launching parallel mappers each receiving records from a HBase region, filling in missing genotypes, concatenating records with the same row-key, and outputting final results into TPED files.

Spark Schema

Spark [7] is a distributed engine that embraces the ideas of MapReduce and Resilient Distributed Dataset (RDD). It can save intermediate results in the form of RDD in memory, and perform computation on them. Also, its computations are lazily evaluated, which means the execution plan can be optimized since it tries to include as many computational steps as possible. As a result, it is ideal for iterative computations such as sorted merging. We implement our optimized Spark schema on Spark-2.1. It has three stages as shown in Figure 4. Stage I involves loading raw data as RDDs, filtering, and mapping RDDs to paired-RDDs with keys (chromosome-genomic position) and values (reference allele, individual ID and genotype). This stage ends with a sort-by-

key shuffling that repartitions and sorts PairRDD records so that records with the same key are aggregated together. In Stage II, aggregated PairRDD records of the same key are merged into TPED format and converted back to RDD records for outputting. However, Spark's native family of group-by-key functions cannot be used here because their default partitioner is hash-based and different from the range-based partitioner used by previous sort-by-key function. Consequently, the merged results would be reshuffled into an unsorted status. We therefore optimize the merging to bypass these functions, being performed locally without data reshuffling to ensure both order and high speed. Finally in Stage III, merged RDD records are saved as TPED files.

Execution parallelism has an important impact on the performance. To maximize performance, the number of parallel tasks is set to the number of input files. In this way, the data locality is maximized and each task is assigned a proper amount of work. In addition, unlike using MapReduce or HBase, when performing sorting by key, no explicit sampling is needed because Spark keeps track of the number of records before determining repartition boundaries.

Results

We conduct all experiments using Amazon's Elastic MapReduce (EMR) service. Within the infrastructure, we choose EC2 working nodes of m3.xlarge type, which has four High Frequency Intel Xeon E5-2670 v2 (Ivy Bridge) Processors and 15GB memory. We use a dataset consisting of the VCF files of 93 individuals [22] generated from Illumina's BaseSpace software.

Overall Performance Analysis of Clustered-based Schemas

Our primary goal is to explore the scalability of the three schemas on input data size and available computing resources, namely CPUs. To achieve this, in this experiment we adjust the number of input files from 10 to 93, with an approximate total uncompressed size from 2.5 G to 20 G, and conduct the experiment using a varying number of working nodes from 3 to 18, namely 12 to 72 cores.

As Figure 5 shows, for all three schemas, given a fixed number of cores, the execution time increases linearly with the increased number of input files. On the one hand, the increasing trend is apparent with fewer cores because each core is fully utilized and the more input files, the larger number of parallel tasks are assigned to it. For example, given 12 cores, as the file number

increases from 10 to 93, the execution time increases from 739 to 2,281 seconds for the MapReduce schema, from 375 to 2,751 seconds for the HBase schema, and from 361 to 1,699 seconds for the Spark schema, respectively. On the other hand, with relatively more cores such as 72, this linear increasing trend is less pronounced because there are more cores than tasks so that all cores are assigned at most one task. We also notice that when input file size is small to moderate, the Spark schema does not always show consistent improvement in terms of execution time when using more cores, for example, the intersection of curves of 24 and 72 cores in Figure 5c. This phenomenon is attributed to the limitation of Spark's internal task assignment policy which gives rise to the possibility that some nodes are assigned more than one tasks while others remain idle.

In another experiment in which the input file number is fixed at 93, the core number increases from 12 to 72 (Figure 6). For all three schemas, execution time is reduced with more cores, from 2,281 to 514 seconds for MapReduce, from 2,751 to 591 seconds for HBase, and from 1,699 to 460 seconds for Spark, respectively. Therefore, all three schemas demonstrate nice scalability on input data size and computing resources.

The Anatomic Performances Analysis of Cluster-based Schemas

Another important goal of our study is to identify potential performance bottlenecks, so we evaluate the execution time of each phase/stage of all three schemas. Figure 7 shows the trend of anatomic computing time spent on merging increasing number of VCF files using 48 cores. For the MapReduce schema (Figure 7a), its two phases account for a comparable proportion of total time and both show a linear or sublinear increasing pattern. For the three phases of the HBase schema (Figure 7b), they generally scale well with the input file number. Meanwhile, the second phase becomes more dominant with more input files owing to the larger amount of shuffled data during the writing of HFiles. However, we do not consider it as a bottleneck since all tasks of this phase are parallelized with no workload or computational hotspot. We do not observe an obvious super-linear increment pattern from the figure either. Finally, Figure 7c shows the time costs of three stages of the Spark schema. They show a uniform increasing trend with input file number. Among them, the second one takes up a considerable proportion of the total execution time as it has the relatively expensive sort-by-key shuffling operation. Although no data is shuffled in the first stage, its time lapse is close to that of the second stage. This is because at the end of the first

stage, data are sampled for determining the boundaries used by sort-by-key's range partitioner. This operation demands a considerable execution time because it scans all the data and balances them if necessary.

Put together, these results suggest that all phases/stages of the three schemas scale well on input data size. Therefore we are not expecting to see any bottleneck when dealing with even larger scale of data.

Comparisons between Single Machine Based Methods and Cluster-based Schemas

Another intriguing question in our experiments is how much can our schemas outperform the current single machine based methods and applications. To achieve this, in the first experiment, we test the performance of merging 40 VCF files into one VCF using VCFTools v4.2 as a benchmark. As shown in Table 2, VCFTools takes 30,189 seconds while the fastest cluster-based schema, MapReduce-based, takes only 484 seconds using 72 cores, which is 62-fold faster. In the second experiment, we test the performance of merging of VCF files into a TPED using multiway-merge based implementation as a benchmark. We choose to implement multiway-merge because,

to our best knowledge, currently no software/application is available to perform this task with a simple call. Rather, VCF files need to be manually converted to individual TPED files first which in turn are merged together using merging utility of PLINK which essentially is based on multiway-merge. Our multi-way merge implementation saves this manual conversion step, and is also more concise and efficient than PLINK. The multiway-merge implementation is tested on a single node while the three schemas on a cluster of nodes with 72 cores. Initially with fewer input files, the execution time difference is 399 seconds or about 2.8-fold between multiway-merge and the fastest cluster-based schema, MapReduce. However, this difference becomes significant with more input files. For example, the largest difference is 5,585 seconds or about 13-fold (Figure 8) on merging 93 files. As an extreme test of merging 642 VCF files (not shown), the computing time is 1,228 minutes for multiway merge implementation versus 11.3 minutes of the MapReduce schema running on a 400-core cluster, more than 100-fold of speed up.

We also compare the performances among the three schemas all of which are evaluated on a 72-core cluster with increasing number of files as inputs (Figure 8). It turns out that the three schemas have comparable performance. More specifically, MapReduce-based schema performs best with

small input size, HBase-based schema performs best with moderate input size, while Spark-based schema performs best with large input size. The rationale behind such an observation is when the input data size is small, MapReduce can make the most usage of computing resources because it has a constant 25 parallel jobs (one for each of chromosomes 1-22, X Y and M (Mitochondria)) in its second phase. In contrast, Spark has much fewer tasks with a number equals to the number of input files for achieving maximized data-task locality. When the input data size is moderate, HBase triumphs due to its internal sorting and relative compact storage format of intermediate data. When the input data size is large, Spark-based schema outperforms the other two owing to its least number of data shuffling (only one), execution plan optimization, and ability to cache intermediate results in memory. We caution that the computing time may fluctuate depending on the genomic location profile of input files as well as the data loading balance of the HDFS.

Discussion

In this report, we describe three cluster-based schemas running on Apache Hadoop (MapReduce), HBase and Spark platforms respectively for performing sorted merging of variants identified from WGS. We manage to show that all three schemas are highly scalable on both input data size and

computing resources, suggesting that large scale sequencing of variant data can be merged efficiently given computing resources that are readily available in the cloud. We also show that even with a moderate-sized cluster and input data, all three schemas are able to significantly outperform the broadly-used, single-machine based VCFTools and multiway-merge implementation. We expect a much more significant performance improvement when merging a much larger scale of data using a larger cluster or the cloud.

Unlike normal merging, efficient sorted merging of many large tables has always been a difficult problem in the field of data management. Multiway-merge is the most efficient single-machine based method for sorted merging, but its performance is limited by the disk I/O [25]. Sorted merging also places challenges to distributed system based solutions because neither the efficient hash-based merging nor caching the intermediate table in shared memory is feasible [26]. Although a utility named total-order-joining is provided by the Hadoop for addressing this problem, it suffers from both network communication and local disk I/O bottleneck, thus is not scalable [16, 27]. In contrast, our schemas divide this problem into different phases of tasks each

conquered in parallel to bypass these bottlenecks and achieve maximum parallelism and scalability. Furthermore, in addition to merging sequencing variant data, the schemas can be generalized for other key-based, sorted merging problems that are frequently encountered in genetics and genomics data processing. As an example, they can be slightly modified to merge multiple BED format files such as ChIP-seq peak lists [28] and other genomic regions of interest. Another potentially useful feature is that, unlike traditional sorted merging algorithms which usually require presorted inputs for better performance, our schemas are free of such a requirement.

Finally, in light of the different features and specialties of the three platforms, each of the three schemas we developed has its own advantages and disadvantages in different application scenarios as summarized in Table 1. For example, the MapReduce schema is good for static one-time, non-incremental merging on small to moderate-sized data since it can have the most parallel jobs, the least overhead, and the most transparent workflow. The HBase schema, supported by data warehousing technologies, fits for incremental merging since it does not need to re-merge existing results with new ones from the scratch only if the incremental merging is performed on the same

chromosomes. Also, it provides highly-efficient storage and On-Line Analytical Processing (OLAP) on merged results. The Spark schema is ideal for merging large scale of data because it has the least data shuffling and keeps intermediate results in memory. A bonus brought by Spark is that subsequent statistical analyses can be carried out directly on the merged results using its rich set of parallel statistical utilities.

Availability and Requirements

Project name: CloudMerge

Project home page: https://github.com/xsun28/CloudMerge

Operating system(s): Linux

Programming language: Java

Other requirements: Java 1.7 or higher, Hadoop-2.7, HBase-1.3, Spark-2.1

License: Apache License 2.0

Availability of Data and Materials

The source code of the project is available on GitHub. The original 93 individual VCF files used in our experiments are from the Consortium on Asthma among African-ancestry Population in the Americas (CAAPA) [22]. To conceal the potential individual identifiable genotype information from the public, we encrypt the authentic genomic location of all VCF files to generate a new batch of encrypted VCF files, which are available on AWS S3 as https://s3.amazonaws.com/xsun316/encrypted/encrypted.tar.gz. We also provide sample results of merging 93 VCF files into either one VCF or one TPED file using our cluster-based schemas, which are available on AWS S3 as https://s3.amazonaws.com/xsun316/sample_results/result.tar.gz.

Abbreviations

VCF: Variant Call Format; GWAS: Genome Wide Association Studies; WGS: Whole Genome Sequencing; WES: whole exome sequencing; AWS: Amazon Web Service; CGDM: Collaborative Genomic Data Model; SAM/BAM: Sequence/Binary Alignment/Map; RDD: Resilient Distributed Dataset; DAG: Directed Acyclic Graph; EMR: Elastic-MapReduce; CAAPA: Consortium on Asthma among African-ancestry Population in the Americas;

Consent for Publication

Not applicable

Competing Interests

The authors declare they have no competing interests.

Authors Contributions

J.G. proposed the problem. X.S., F.W. initiated this project. X.S. designed and implemented the CloudMerge project. X.S. drafted the manuscript. X.S., J.P., F.W. and Z.Q. revised the manuscript.

Acknowledgements

We are grateful for Duck, Alyssa Leann for helping on revising and rephrasing the manuscript.

References

1. Massie M, Nothaft F, Hartl C, Kozanitis C, Schumacher A, Joseph AD, et al. Adam: Genomics formats and processing patterns for cloud scale computing. University of California, Berkeley Technical Report, No UCB/EECS-2013. 2013;207.
2. Siretskiy A, Sundqvist T, Voznesenskiy M and Spjuth O. A quantitative assessment of the hadoop framework for analyzing massively parallel dna sequencing data. Gigascience. 2015;4 1:26.
3. Burren OS, Guo H and Wallace C. VSEAMS: a pipeline for variant set enrichment analysis using summary GWAS data identifies IKZF3, BATF and ESRRA as key transcription factors in type 1

diabetes. Bioinformatics. 2014;30 23:3342-8. doi:10.1093/bioinformatics/btu571.

4. Apache Hadoop. http://hadoop.apache.org/. Accessed 10 Oct 2017.

5. Dean J and Ghemawat S. Mapreduce: Simplified data processing on large clusters. Commun Acm. 2008;51 1:107-13. doi:Doi 10.1145/1327452.1327492.

6. Vora MN. Hadoop-HBase for large-scale data. In: *Computer science and network technology (ICCSNT), 2011 international conference on* 2011, pp.601-5. IEEE.

7. Zaharia M, Chowdhury M, Das T, Dave A, Ma J, McCauley M, et al. Resilient distributed datasets: A fault-tolerant abstraction for in-memory cluster computing. In: *Proceedings of the 9th USENIX conference on Networked Systems Design and Implementation* 2012, pp.2-. USENIX Association.

8. Schatz MC. CloudBurst: highly sensitive read mapping with MapReduce. Bioinformatics. 2009;25 11:1363-9. doi:10.1093/bioinformatics/btp236.

9. Langmead B, Schatz MC, Lin J, Pop M and Salzberg SL. Searching for SNPs with cloud computing. Genome Biol. 2009;10 11:R134. doi:10.1186/gb-2009-10-11-r134.

10. Wang S, Mares MA and Guo YK. CGDM: collaborative genomic data model for molecular profiling data using NoSQL. Bioinformatics. 2016;32 23:3654-60. doi:10.1093/bioinformatics/btw531.

11. AWS Genomics Guide. https://d0.awsstatic.com/Industries/HCLS/Resources/AWS_Genomics_WP.pdf. Accessed 10 Oct 2017.

12. Gruber K. Google for genomes. Nature Research, 2014.

13. Purcell S, Neale B, Todd-Brown K, Thomas L, Ferreira MA, Bender D, et al. PLINK: a tool set for whole-genome association and population-based linkage analyses. Am J Hum Genet. 2007;81 3:559-75. doi:10.1086/519795.

14. Mohammed EA, Far BH and Naugler C. Applications of the MapReduce programming framework to clinical big data analysis: current landscape and future trends. BioData Min. 2014;7:22. doi:10.1186/1756-0381-7-22.

15. Huang H, Tata S and Prill RJ. BlueSNP: R package for highly scalable genome-wide association studies using Hadoop clusters. Bioinformatics. 2013;29 1:135-6. doi:10.1093/bioinformatics/bts647.

16. White T. Hadoop: The definitive guide. " O'Reilly Media, Inc."; 2012.

17. Silberschatz A, Korth HF and Sudarshan S. DatabaseSystem Concepts. 2010.

18. Danecek P, Auton A, Abecasis G, Albers CA, Banks E, DePristo MA, et al. The variant call format and VCFtools. Bioinformatics. 2011;27 15:2156-8. doi:10.1093/bioinformatics/btr330.

19. Multiway-Merge Algorithm. https://en.wikipedia.org/wiki/K-Way_Merge_Algorithms. Accessed 10 Oct 2017.

20. Chang F, Dean J, Ghemawat S, Hsieh WC, Wallach DA, Burrows M, et al. Bigtable: A distributed storage system for structured data. Acm T Comput Syst. 2008;26 2 doi:Artn 4

10.1145/1365815.1365816.

21. Genomes Project C, Abecasis GR, Altshuler D, Auton A, Brooks LD, Durbin RM, et al. A map of human genome variation from population-scale sequencing. Nature. 2010;467 7319:1061-73. doi:10.1038/nature09534.

22. Mathias RA, Taub MA, Gignoux CR, Fu W, Musharoff S, O'Connor TD, et al. A continuum of admixture in the Western Hemisphere revealed by the African Diaspora genome. Nat Commun. 2016;7:12522. doi:10.1038/ncomms12522.

23. Kwon Y, Balazinska M, Howe B and Rolia J. A study of skew in mapreduce applications. Open Cirrus Summit. 2011;11.

24. O’Neil P, Cheng E, Gawlick D and O’Neil E. The log-structured merge-tree (LSM-tree). Acta Informatica. 1996;33 4:351-85.

25. Sedgewick R and Flajolet P. An introduction to the analysis of algorithms. Addison-Wesley; 2013.

26. Özsu MT and Valduriez P. Principles of distributed database systems. Springer Science & Business Media; 2011.

27. Miner D and Shook A. MapReduce Design Patterns: Building Effective Algorithms and Analytics for Hadoop and Other Systems. " O'Reilly Media, Inc."; 2012.

28. Chen L, Wang C, Qin ZS and Wu H. A novel statistical method for quantitative comparison of multiple ChIP-seq datasets. Bioinformatics. 2015;31 12:1889-96.

Figure legends

Figure 1. Converting VCF files to TPED. Left tables are input VCF files. Right table is the merged TPED file. Records are filtered out if their Filter value doesn’t equal to ‘PASS’ (Pos

10147). Individual genotypes with the same genomic location that exist in any VCF file are aggregated together on one row. The resulting TPED file thus has an inclusive set of sorted genomic locations from all VCF files.

Figure 2. The workflow chart of MapReduce schema. It consists of two phases: In the first phase, input VCF records are filtered, grouped around chromosomes into bins, and mapped into key-value records. Two samplings are performed to generate partition lists of chromosomes. In the second phase, parallel jobs of specified chromosomes are launched. Within each job, records from corresponding bins are loaded, partitioned, sorted and merged by genomic locations before being outputted as TPED files.

Figure 3. The workflow chart of HBase schema. The workflow is divided into three phases. The first one is a sampling, filtering and mapping phase. A MapReduce job samples out VCF records whose genomic positions are used as region boundaries when creating the HBase table. Only qualified records are mapped as key-values and saved as Hadoop sequence files. The second phase is HBase bulk loading in which a MapReduce job load and writes records outputted from the

previous phase, aggregating them into corresponding regional HFiles in the form of HBase's row key and column families. Finished HFiles are moved into data folders on region servers. In the third phase, we launch parallel scans over regions of the whole table to retrieve desired records which are subsequently merged and exported as TPED files.

Figure 4. The workflow chart of Spark schema. It is a single Spark job consisting of three stages. In the first stage, VCF records are loaded, filtered, and mapped to PairRDDs with keys of genomic position and values of genotype. The sort-by-key shuffling spans across the first two stages, sorting and grouping together records by keys. Then grouped records with the same key are locally merged into one record in TPED format. Finally, merged records are exported as TPED files.

Figure 5. The scalability of clustered based schemas on input size. Subfigures a, b and c refer to MapReduce, HBase and Spark schemas respectively. As input file number increases from 10 to 93, the time cost of all three schemas with 12, 24 or 72 cores show at most a linear increasing trend which suggests good scalabilities. The HBase schema with 12 cores has the largest increase

from 375 to 2,751 seconds, or 7.3-fold.

Figure 6. The scalability of cluster based schemas on available computing core number. Subfigures a, b and c refer to MapReduce, HBase and Spark schemas respectively. In these experiments, the number of input files is fixed at 93. As core number increases from 12 to 72, the time cost of the three schemas decrease linearly until a plateau is reached where computing resources become excessive. The Spark schema shows lowest reduction of time cost from 1,699 to 460 seconds, or 3.7-fold. These results suggest good scalabilities of these schemas on computing resources.

Figure 7. The performance anatomy of cluster-based schemas on increasing input size. The number of cores in these experiments is fixed at 48. All phases of the three schemas show good scalabilities with input data size. **a)** MapReduce schema: The two MapReduce phases have a comparable time cost, increasing 3.0- and 2.2-fold respectively as input file number increases from 10 to 93. **b)** HBase schema: The time spent in each phase increases 2.0-, 2.7- and 2.2-fold respectively as input file number increases from 10 to 93. The bulk loading and exporting phases

together take up more than 90% of total time expense. **c)** Spark schema: The time cost increases 3.1-, 3.0- and 3.4-fold respectively for the three stages as input file number increases from 10 to 93. Like the HBase schema, the first two stages of the Spark schema together account for more than 90% of total time cost.

Figure 8. Performance comparison among multiway-merge implementations and cluster-based schemas: Firstly, we compare of the performances of the three schemas with that of the multiway-merge implementation. When input file number is 10, the time differences between multiway-merge and our schemas are relatively small, ranging from 2- to 2.8-fold. As file number increases to 93, the differences turn out to be more significant, ranging from 10.2- to 13.1-fold. Secondly, we compare the performances among the three schemas which are comparable to each other regardless of the input file number. MapReduce schema has best performance in merging 10 files; HBase schema performs best in merging 20, 40 and 60 files; Spark schema is fastest in merging 93 files.

Tables

Table 1. Performance comparisons of VCTools versus MapReduce, HBase and Spark schemas

	VCFTools	MapReduce	HBase	Spark
**Time cost (seconds)**	30,189	484	577	596
**Fold (faster)**	-	62.4	52.3	50.7

We compare the time cost of VCFTools and our three schemas using 72 cores on merging 40VCF files into one VCF file. VCFTools takes more than 30,000 seconds to finish. In contrast, all three schemas take less than 600 seconds to finish. MapReduce schema has the largest performance improvement which is about 62-fold.

Table 2. Pros and Cons of MapReduce, HBase and Spark schemas

Schemas	***Pros***	***Cons***
**MapReduce**	• Simple architecture and least overhead.<br>• Best parallelism for small input size (<= 20).<br>• Good for one-time merging.<br>• Performance is stable.	• Merging is not incremental.

**HBase**	• Good for intermediate input size (>=20 and <=100).<br>• Supports incremental merging.<br>• Supports On-Line Analytical Processing (OLAP).<br>• Best storage efficiency.	• Users must determine region number in advance.<br>• Has most local I/O.<br>• Complex performance tuning.
**Spark**	• Good for large input size (>100).<br>• Keeps intermediate results in memory and least local I/O.<br>• Good for subsequent statistical analysis on merged results.	• Possibly weakened data locality during loading.<br>• Slight unstable performance when computing resources exceeds needs of input size.<br>• Actual execution plan is not transparent.<br>• Complex performance tuning.

Each of the three distributed systems has its own specialties and limitations. As a result, the schemas running on them have different pros and cons, and application scenarios as listed above.

Figure1

Click here to download Figure Figure1.pdf

VCF File 1

Chr	Pos	Ref	Alt	Filter	…	Genotype
1	10147	A	T	q20	…	1/0:43
1	10240	T	G	PASS	…	1/0:5
…	…	…	…	…	…	…
Y	11590	G	C	PASS	…	0/0:10

VCF File 2

Chr	Pos	Ref	Alt	Filter	…	Genotype
1	10186	G	A	PASS	…	1/0:9
1	10240	T	G	PASS	…	1/1:11
…	…	…	…	…	…	…
Y	11872	G	T	PASS	…	0/1:10

Genotypes

Chr	Rs	Distance	Pos	Ind_1	Ind_2
1	.	0	10186	G G	G A
1	.	0	10240	T G	G G
…	…	…	…	…	…
Y	.	0	11590	G G	G G
Y	.	0	11872	G G	G T

Merged TPED file

Figure2

Click here to download Figure Figure2.pdf

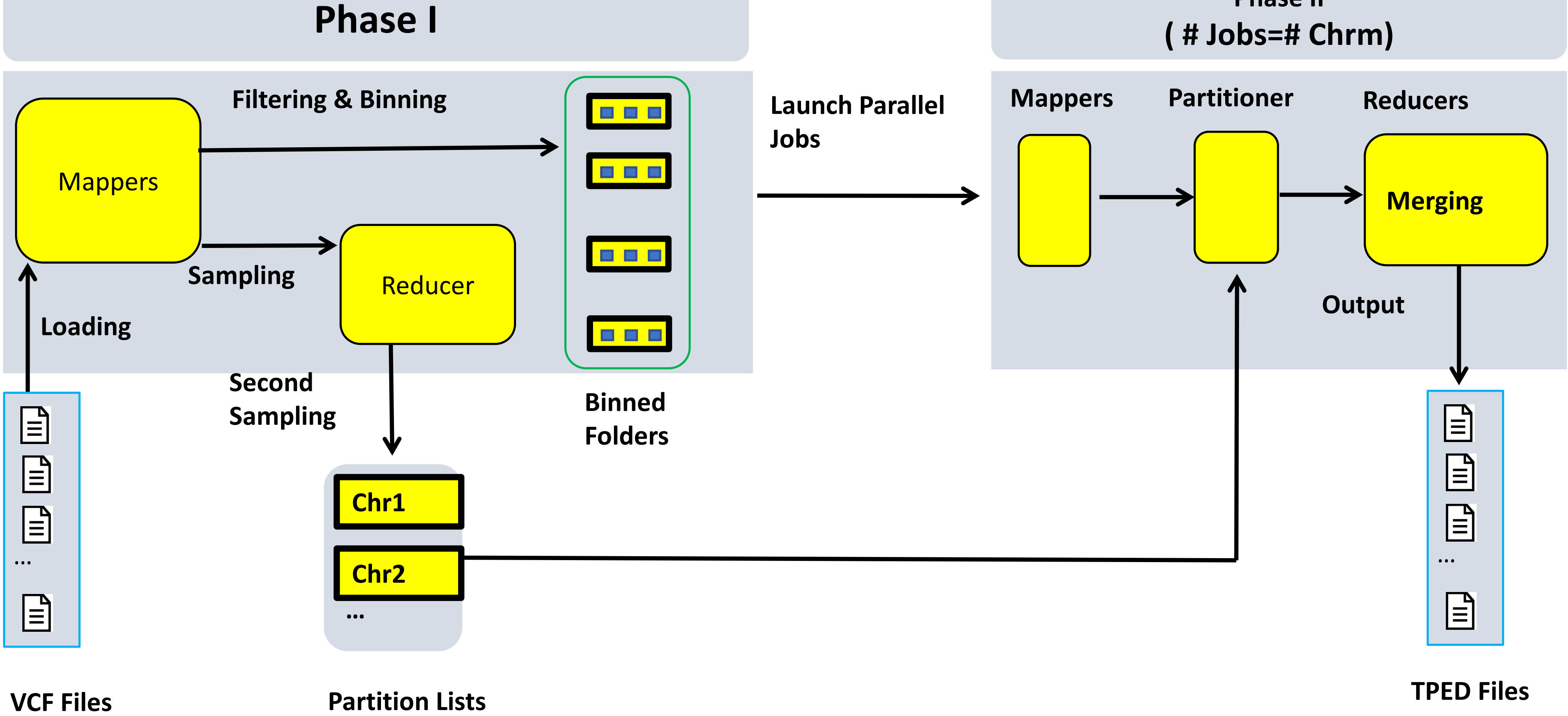

Figure3

Click here to download Figure Figure3.pdf

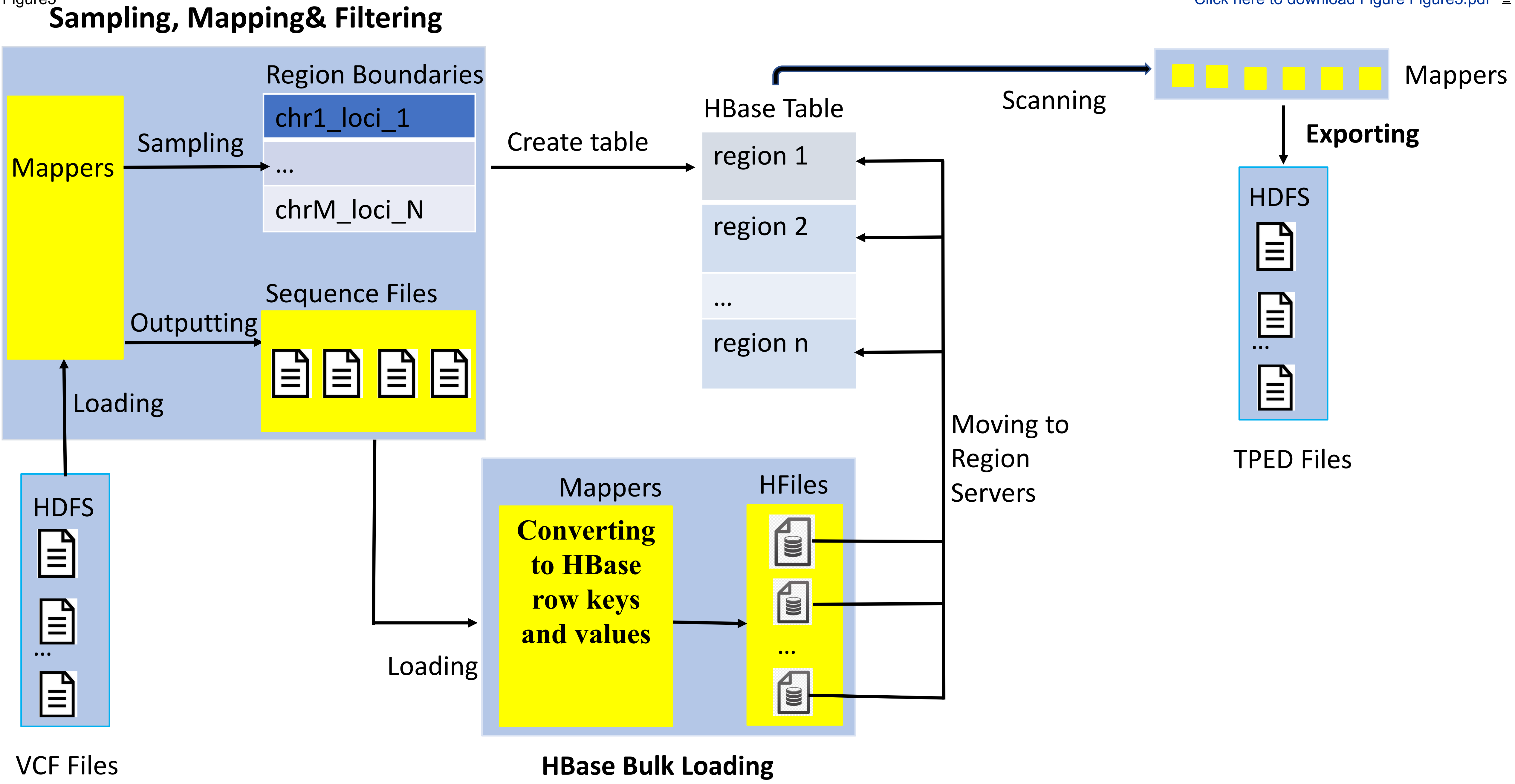

Figure4

Click here to download Figure Figure4.pdf

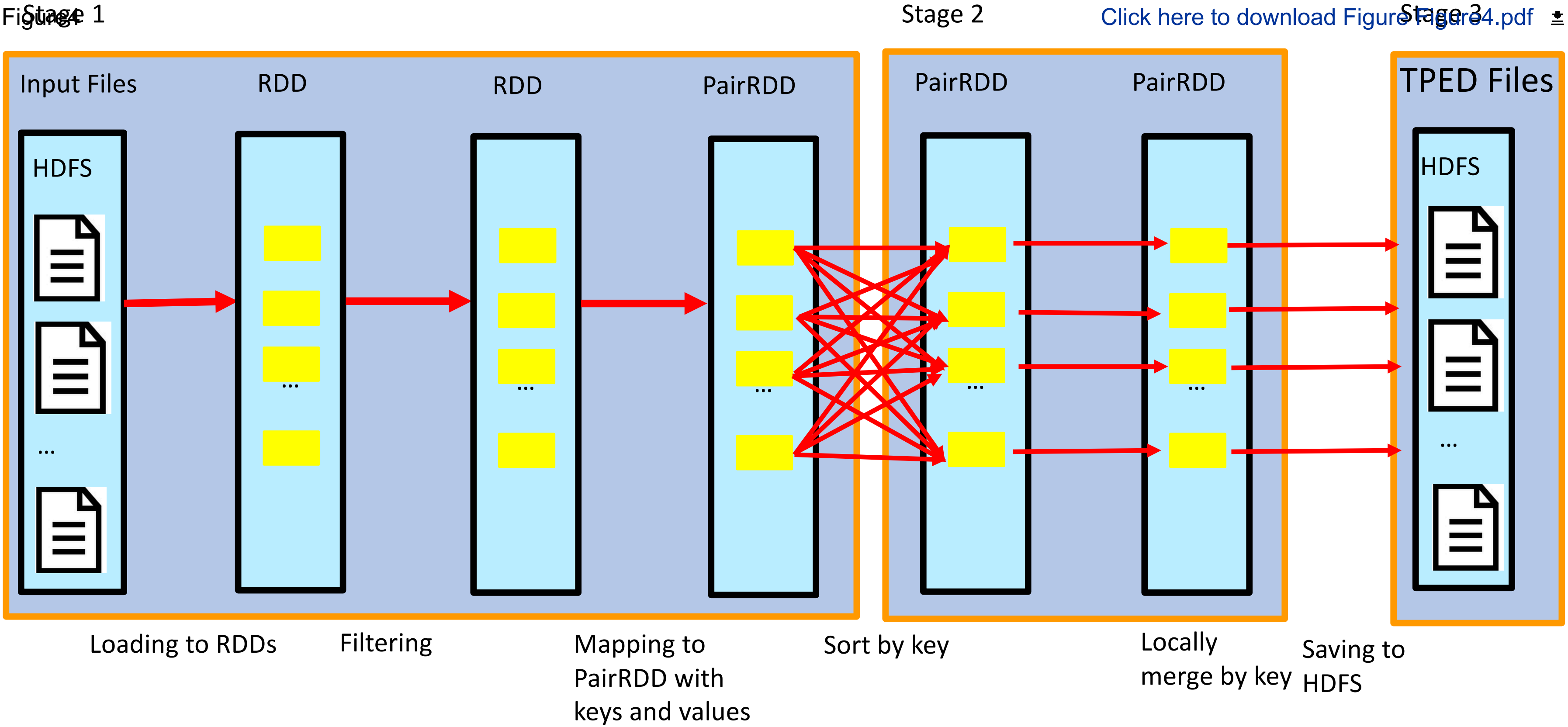

Figure5

Click here to download Figure Figure5.pdf

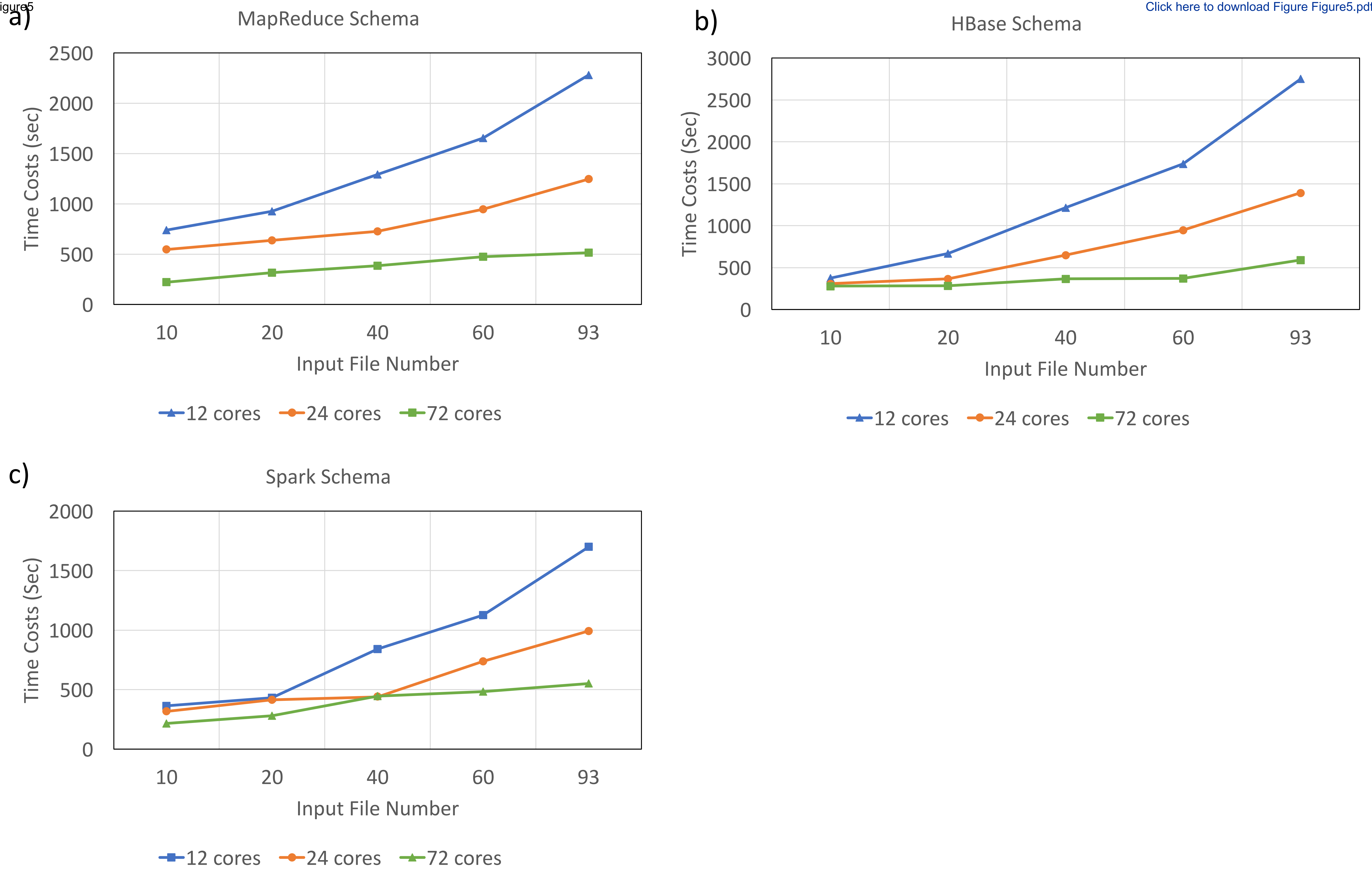

Figure6
Click here to download Figure Figure6.pdf

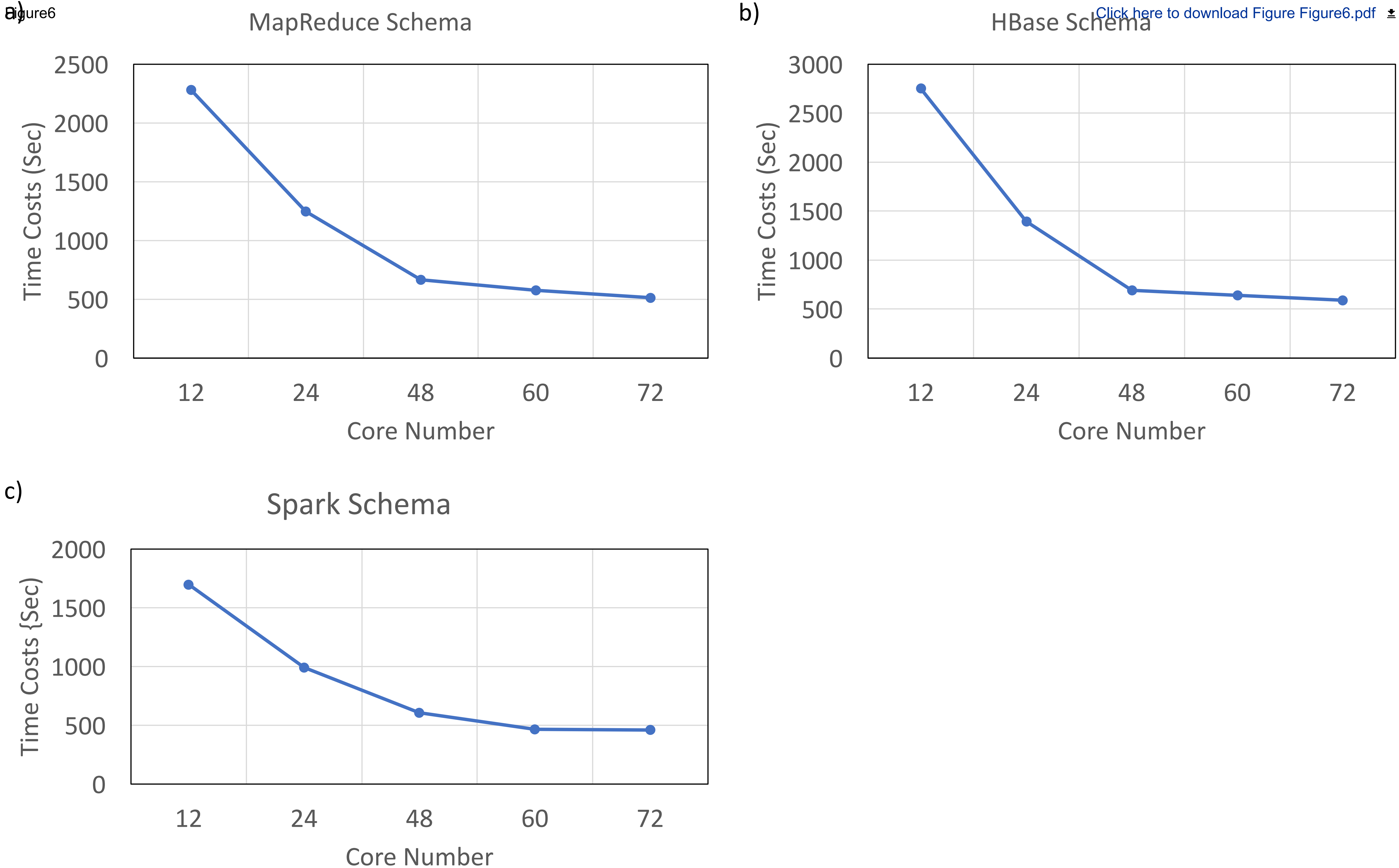

Figure7

Click here to download Figure Figure7.pdf

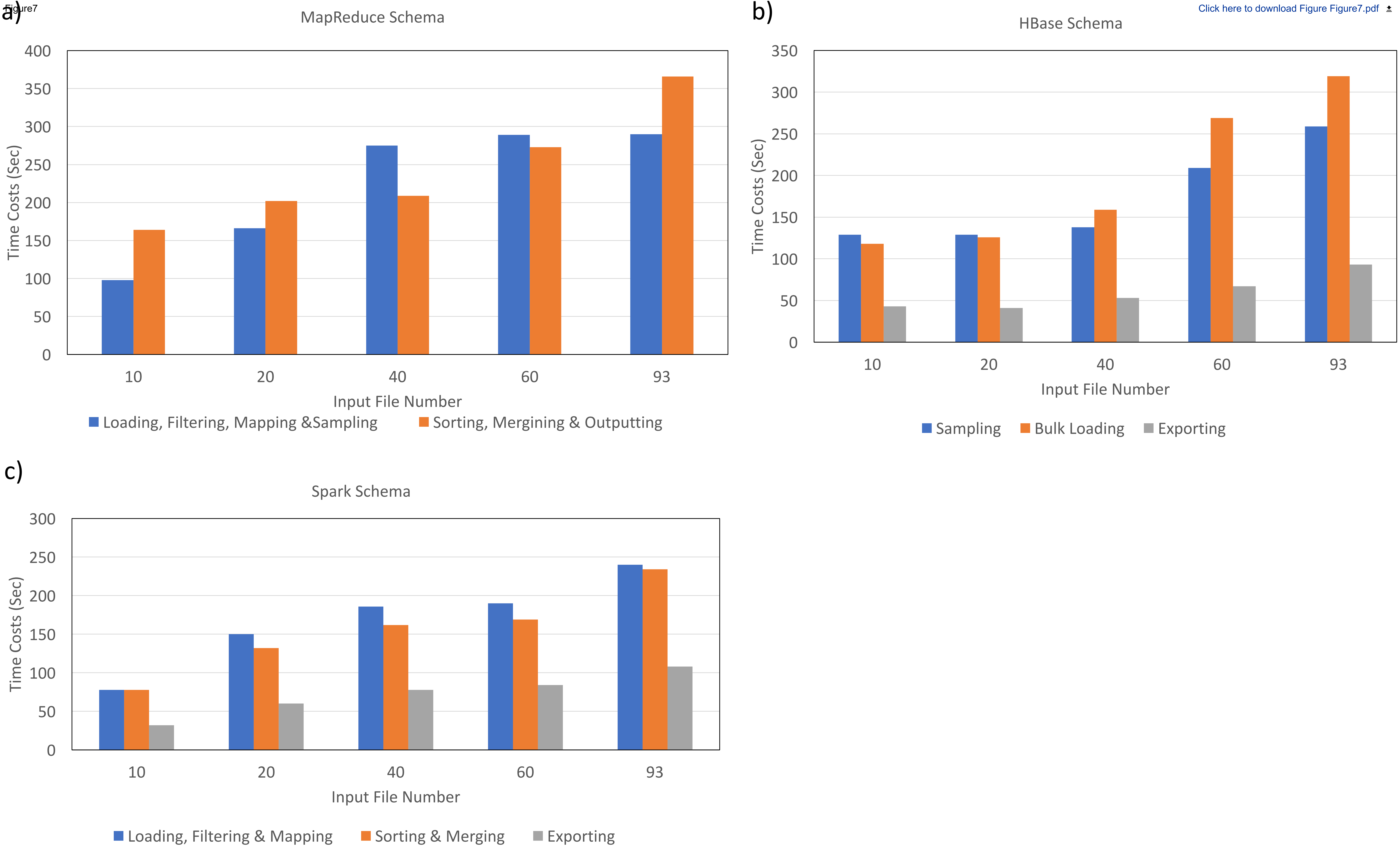

Figure8

Click here to download Figure Figure8.pdf

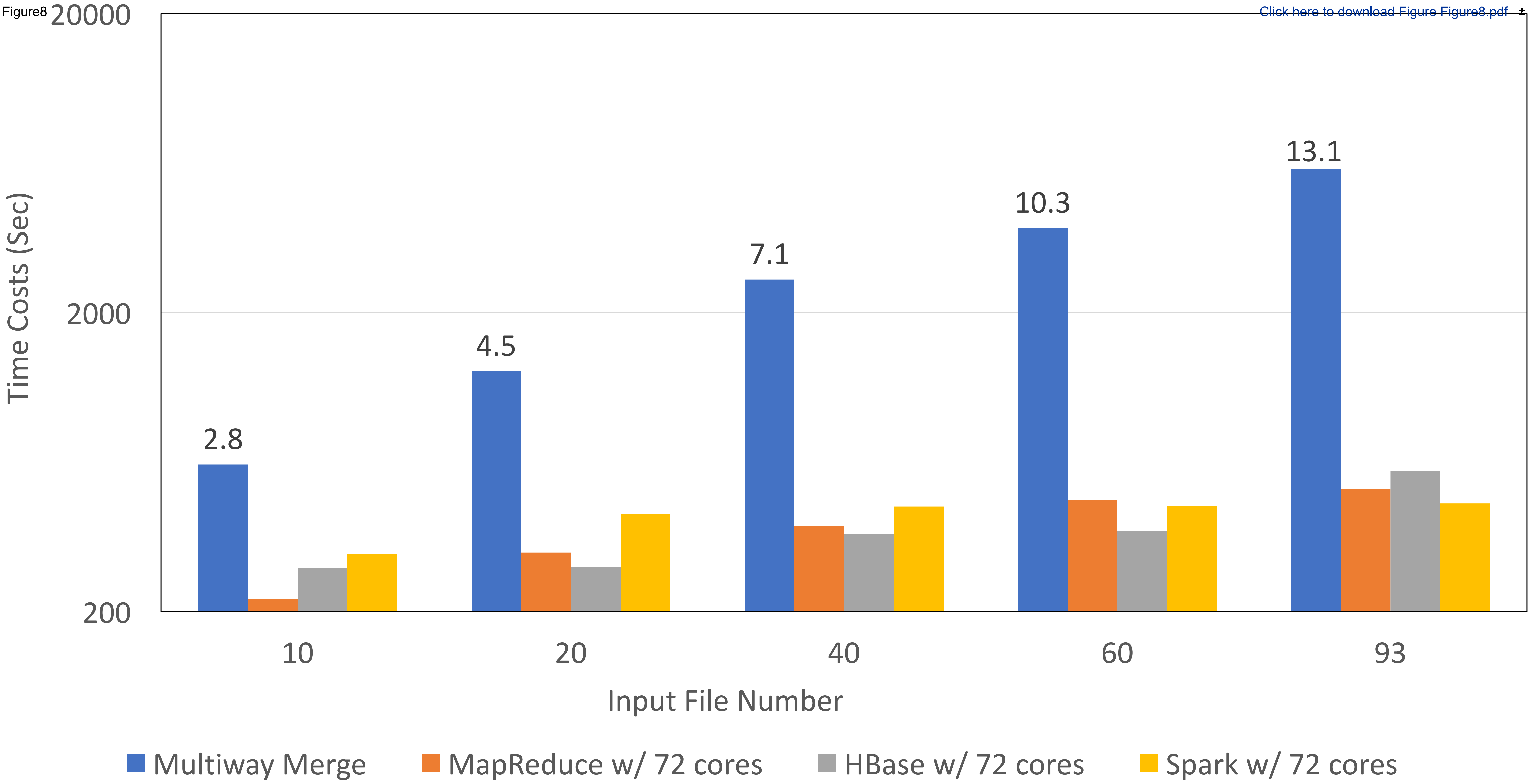

EMORY
ROLLINS SCHOOL OF PUBLIC HEALTH
Department of Biostatistics and Bioinformatics

October 12, 2017

Laurie Goodman, PhD
Editor-in-Chief
GigaScience

Dear Dr. Goodman:

On behalf of our colleague, Xiaobo Sun, Jingjing Gao and Peng Jin, I am pleased to submit a manuscript titled:

Optimized Distributed Systems Achieve Significant Performance Improvement on Sorted Merging of Massive Omics Data

for publication consideration in GigaScience as a Technical Note.

In the present manuscript, we describe three novel optimized schemas, running on Apache Hadoop, HBase and Spark respectively, for performing sorted merging on Omics data. Sorted merging is one of important data manipulation tools for Omics data, for example, the merged VCF or TPED files are required to perform statistical analysis in association studies on sequencing data. However, most existing tools for handling this task, such as VCFTools and PLINK, are running on single machine and implemented based on the multiway-merge algorithm. As a result, they suffer from the limitations of disk I/O and become very inefficient in face of large data size. The recent distributed systems offer an alternative solution. However, without optimized working schemas, naively using these systems does not lead to scalability.

In this study, we custom design optimized schemas for three Apache big data platforms. All three schemas are able to overcome the bottleneck problem by maintaining cluster's workload balance and achieving maximum parallelism. We have compared our schemas with VCFTools on merging 40 VCF files into a single one, as well as with a multiway-merge based implementation on merging up to 93 VCF files into a single TPED file. It turns out that even using a moderate sized cluster, we can archive speedup up to 62-fold compared to VCFTools. All three schemas show good scalability on both input size and number of cores, suggesting given enough computing resources we can guarantee the performance even in face of very large scale of data. Therefore our findings provide generalized scalable schemas for performing sorted merging on genetics and genomics data using these Apache distributed systems. Our schemas can be easily generalized to merge other types of Omics data such as ChIP-seq peak lists. **Therefore, we believe our schemas will have a high impact in the omics field as we enter the big data era.**

We hereby confirm that we do not have any potential competing interests and all authors have approved the manuscript for submission. We also confirm that the manuscript has not been submitted for publication elsewhere.

In light of the content, we suggested the following researchers as potential reviews for our manuscript: Dongxiao Zhu (Wayne State, email: ct4442@wayne.edu), Huanmei Wu (Indiana University–Purdue University Indianapolis, email: hw9@iupui.edu), W. Jim Zheng (UT Health at Houston, email: Wenjin.J.Zheng@uth.tmc.edu), Edmon Begoli (Oak Ridge National Laboratory, email: begolie@ornl.gov), Ulf Leser (Humboldt-Universität zu Berlin, email: leser@informatik.hu-berlin.de).

Thank you very much for your kind editorial assistance.

Sincerely,

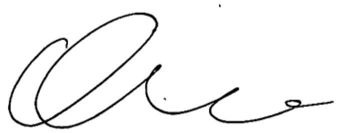

Zhaohui (Steve) Qin, Ph.D.
Associate Professor
Department of Biostatistics and Bioinformatics
Emory University
Atlanta, GA 30322

Fusheng Wang, Ph.D.
Assistant Professor
Department of Biomedical Informatics
Department of Computer Science
Stony Brook University
2313D Computer Science,
Stony Brook, NY 11794-8330
