# Supplementary material for: Optimized distributed systems achieve significant performance improvement on sorted merging of massive VCF files
Source: Gigascience. 2018 May 10;7(6):giy052. doi: 10.1093/gigascience/giy052 (PMC6007233; doi:10.1093/gigascience/giy052)
Supplement: GIGA-D-17-00267_Revision_1.pdf [file giy052_giga-d-17-00267_revision_1.pdf]

GigaScience

Optimized Distributed Systems Achieve Significant Performance Improvement on Sorted Merging of Massive VCF Files

--Manuscript Draft--

Manuscript Number:	GIGA-D-17-00267R1
Full Title:	Optimized Distributed Systems Achieve Significant Performance Improvement on Sorted Merging of Massive VCF Files
Article Type:	Technical Note
Funding Information:	
Abstract:	Background: Sorted merging of genomic data is a common data operation necessary in many sequencing-based studies. It involves sorting and merging genomic data from different subjects by genomic locations. In particular, merging a large number of Variant Call Format (VCF) files are frequently encountered in large scale whole genome sequencing or whole exome sequencing projects. Traditional single machine based methods become increasingly inefficient when processing hundreds or even thousands of VCF files due to the excessive computation time and I/O bottleneck. The distributed systems and the more recent cloud-based systems offer an attractive solution. However, carefully designed and optimized working flow patterns and execution plans (schemas) are required to take full advantage of the increased computing power while overcoming bottlenecks to achieve high performance.<br><br>Findings: In this study, we custom design optimized schemas for three Apache big data platforms, Hadoop (MapReduce), HBase and Spark, to perform sorted merging of large number of VCF files. These schemas all adopt the divide-and-conquer strategy to split the merging job into sequential phases/stages consisting of subtasks which are conquered in an ordered, parallel and bottleneck-free way. In two illustrating examples, we test the performance of our schemas on merging multiple VCF files into either a single TPED or VCF file, which are benchmarked with the traditional single/parallel multiway-merge methods, message passing interface (MPI) based high performance computing (HPC) implementation and the popular VCFTools.<br><br>Conclusions: Our experiments suggest that all three schemas either deliver a significant improvement in efficiency or render much better strong/weak scalabilities over traditional methods. We believe that our findings provide generalized scalable schemas for performing sorted merging on genetics and genomics data using these Apache distributed systems.
Corresponding Author:	Zhaohui Qin<br><br>UNITED STATES
Corresponding Author Secondary Information:	
Corresponding Author's Institution:	
Corresponding Author's Secondary Institution:	
First Author:	Xiaobo Sun
First Author Secondary Information:	
Order of Authors:	Xiaobo Sun
	Zhaohui Qin
	Fusheng Wang
	Jingjing Gao
	Peng Jin
Order of Authors Secondary Information:	

Powered by Editorial Manager® and ProduXion Manager® from Aries Systems Corporation

Response to Reviewers:	Reviewer #1:<br>This paper is a cross between a case study and an application note. As a case study it considers the task of improving the performance of sorted merging of variant call data using parallel computation. As an application note it provides software implementations of tools which can be downloaded and used by bioinformatics practitioners. Although the case study considers sorting variant call data (VCF files) the techniques developed can easily be extended to other coordinate oriented data sets.<br><br>The paper describes in moderate detail the implementation of three different parallelisation techniques based on the Apache distributed platforms: Hadoop (MapReduce), HBase, Spark.<br><br>It compares the performance of these implementations for strong and weak scaling (though the authors do not use this terminology) against each other, and also against non-distributed parallel versions using the widely used package VCFTools, and also a custom built multiway-merge implementation. The authors also consider the performance characteristics of different phases of their parallel implementations to see whether any of them might suffer bottlenecks when scaling to larger data sets.<br><br>The performance outcomes are fairly predictably in favour of the distributed parallel systems. However, the authors have a tendency to overstate the significance of the outcomes, saying that "Traditional single machine based methods are no longer feasible to process big data due to the prohibitive computation time and I/O bottleneck".<br><br>I do not believe that the results of this paper show that single node merging is infeasible, rather they show that distributed parallel techniques can scale to larger data sets. This is something we have known for a long time. The authors also say that "The newly distributed systems have the potential to offer a much needed boost in performance", however, distributed systems are now decades-old ideas. Also, the paper does not really attempt to investigate similar strong and weak scaling for the single node implementations.<br><br>We thank the reviewer's comments. We apologize for making the overstatements. We took the reviewer's advice and have rephrased the two sentences as following.<br><br>On line 21:<br>"Traditional single machine based methods are no longer feasible to process big data due to the prohibitive computation time and I/O bottleneck"<br>Changed to:<br>"Traditional single machine based methods become increasingly inefficient when processing hundreds or even thousands of VCF files due to the excessive computation time and I/O bottleneck."<br><br>On line 23:<br>"The newly distributed systems have the potential to offer a much needed boost in performance"<br>Changed to:<br>"The distributed systems and the more recent cloud-based systems offer an attractive solution."<br><br><br>Following the reviewer's advice, we investigated strong and weak scalabilities for single node implementation. We implemented a parallel multiway-merger running on a single node to test its performance in terms of strong and weak scaling.<br><br>For scaling test, there are three ways to implement the merger on a single node:<br>1.Simply increasing the number of CPUs of the single node. However, the implementation is based on multiway-merge algorithm, which can only allow one writer (single process or thread) to retrieve records from the underlying priority queue data structure. As a result, when using just single multiway-merger, adding more CPUs won't help improving the performance. However, there are two non-trivial ways for scaling which are illustrated below.

Powered by Editorial Manager® and ProduXion Manager® from Aries Systems Corporation

2.We can have multiple multiway-mergers run at the same time where each merger handles the merging of a subset of all files (task-parallelism). Then in the next round, merged files resulted from the previous round are gathered and merged again. This process continues until all files are merged into one file, which will take approximately log(n) rounds where n is the number of input files. This method is essentially our MPI-based HPC implementation running on a single node.

3.We can have multiple multiway-mergers run at the same time where each merger handles a non-overlapping genomic region of the same length from all input files (data-parallelism). Although this strategy sounds promising, it is not quite practical unless the data distribution across all files are uniform or we know the exact data distribution in advance. As a result, it is very difficult to get a balanced workload for each merger unless we sample through all the files to get the data distribution profile. In that case, if we are using single thread/process sampling, this will be slower than the single multiway merge. If we are using multi-thread/process sampling to do this, it essentially becomes the same as our Hadoop implementation running on a single machine but without task coordination offered by YARN. To make a compromise, we can assume the data distribution is uniform and try to assign each merger a genomic region of the same length. Additionally, when reading the input files, we do not want each merger to seek the beginning of its genomic region from the very beginning of the file. Instead we use TabixReader which takes advantage of the Tabix index built on the VCF file to seek directly to the right offset. In addition to the potential workload unbalance problem, this method also has three limitations: 1. Additional time costs are incurred for building the Tabix indices for each file. 2. Tabix can only be used for VCFs, so it cannot be generalized to other data formats. 3. Each process needs to maintain a whole set of file descriptors, Tabix indices, current data blocks of all files in memory, which could cause memory leak or extremely slow garbage collection (GC) time when input data size is large (in our Java implementation, 16 parallel mergers with 93 files consume over 100GB memory. This memory burden may be slightly lower in C/C++ implementation). The following table summarizes the results from weak and strong scaling test running on a r4.8xlarge EC2 instance.

Strong scalability test:
Number of file: 93
# of cores
1
2
4
8
16
Time cost (seconds)
2,410
1,410
959
388
414

From strong scaling test, we can see that as the number of cores increases, the efficiency reduces significantly, indicating poor strong scalability. This is because the more processes, the more imbalanced the workload among processes. Additionally, the saturation of I/O to and from disk as well as higher memory management costs (GC, swap and so on) also contribute to its inefficiency. Results are displayed in Figure 7 in the manuscript.

Weak scalability test:
# of files
10
20
40
80
160
# of cores
1
2

Powered by Editorial Manager® and ProduXion Manager® from Aries Systems Corporation

4
8
16
Time cost (seconds)
242
299
334
417
508

From weak scaling test, we can see that although we fix the data size per process, because of the same reasons as in strong scalability test, the efficiency still decrease significantly. . Results are displayed in Figure 8 in the manuscript.

In conclusion, when input data size is relatively small, the parallel multiway-merge has significant performance advantage over cloud-based method because it avoids the data network transmission latency. However, when input data size is very large which is often the case in real applications, the problem of workload imbalance across all processes, I/O saturation and memory burden worsens significantly. As a result, this method no longer scale well.

In the manuscript, we added “Parallel Multiway-Merge and MPI-based High Performance Computing Implementations” (line 298) and “Strong and Weak Scalabilities” (line 347) sections under the Method to describe how they are implemented and how the tests of strong and weak scalabilities are conducted. We also added a “Comparing Strong and Weak Scalabilities between Apache Cluster-based Schemas and Traditional Parallel Methods” (line 395) section under the Results to show and compare the strong and weak scalabilities of Apache cluster-based schemas with parallel multiway-merge and HPC-based implementations. Finally, we compared the execution speed of both parallel multiway-merge and HPC-based implementations with the Apache cluster-based schemas in the “Comparing Execution Speed between Apache Cluster-based Schemas and Traditional Methods” (line 452) section in the Results.

I believe that the authors have also overstated the scalability of the distributed implementations, saying they have "nice" scalability (nice is a poor choice of adjective in a scientific paper), however, the results shown in figures 5 and 6 show distinct degradation in both strong and weak scaling at higher core counts/input sizes.

We agree with the reviewer and have rephrased the statement to the following (line 379):
“As Figure 6 shows, for all three schemas, given a fixed number of cores, the execution time increases in a slower pace than that of the input data size.”

One thing missing from the paper is a comparison to a distributed parallel version not based on Apache platforms. That is to say, could we achieve similar performance results on a traditional HPC cluster with a shared filesystem to the demonstrated cloud-based solutions? This question is important to consider, because many bioinformatics organisations have access to HPC systems, and heterogenous cloud-HPC platforms are still difficult to manage. It is not clear whether the effort required to implement systems within the Apache frameworks is justified, compared to a comparatively simpler approach on a traditional HPC system.

We thank the reviewer for raising this important question. There are two HPC-based solutions for this problem: data parallelism and task parallelism. However, data parallelism is difficult to achieve because we do not know the data distribution. Randomly splitting and assigning data to HPC nodes causes workload imbalance and jeopardies the overall performance. We could do parallel sampling to acquire the distribution profile, but this will make its workflow the same as that of the MapReduce-based schema with the same operations and the same order: sampling in parallel, dividing the dataset into splits of equal sizes, and assigning the splits to processes to perform the merging. But this implementation is without data locality offered by HDFS and task coordination offered by YARN and thus has a performance no better than the

Powered by Editorial Manager® and ProduXion Manager® from Aries Systems Corporation

MapReduce-based schema. Consequently, we only implement and test the task parallelism using openMPI as following: firstly, we scatter an approximate number of input files to each HPC process across the whole cluster. Each process fetches the assigned files from the NFS system and do the multiway-merging locally. Secondly, in the following rounds, we adopt a tree-structured merging strategy. To be specific, in the second round, processes whose id number is even (process id starts from 0) retrieve merged files from its adjacent process to the right and do the local multiway-merging. In the third round, processes whose id can be fully divided by 4 retrieve merged results of its adjacent process to the right in the second round. For example, process 4 will receive merged results from process 6. This process continues until all the files are merged into a single file. The total number of rounds is log(n).

Strong scalability test:
Number of file: 93

# of nodes (# of processes)
1 (4)
2 (8)
4 (16)
8 (32)
16 (64)
Time cost (seconds)
17745
9105
5377
3098
2093

In the strong scaling test, the efficiency drops significantly as we add more nodes and processes. This is because although adding nodes lead to a better parallelism, the workload imbalance (because it is almost impossible to assign each node exactly the same number of files) is also increased among nodes in every round. Results are displayed in Figure 7 in the manuscript.

Weak scalability test:
# of files
10
20
40
80
160
# of nodes(# of processes)
1 (4)
2 (8)
4 (16)
8 (32)
16(64)
Time cost (seconds)
1311
1799
2142
2919
3777

In the weak scaling test, we fixed the input data size per node. The efficiency drops even more because of increased number of merging rounds. Results are displayed in Figure 8 in the manuscript.

In conclusion, we showed that sorted merging by traditional HPC (task parallelism) shows inferior scalability and are much slower than the methods running on Apache cluster-based framework.

We have added the description of HPC-based implementation and its tests of strong

Powered by Editorial Manager® and ProduXion Manager® from Aries Systems Corporation

and weak scalabilities in the “Parallel Multiway-Merge and MPI-based High Performance Computing Implementations” (line 298) section and “Strong and Weak Scalabilities” (line 347) section.

Perhaps the main contribution of this paper is not the resulting software tools, but the insights provided into parallelisation techniques on the Apache cloud-based platforms. Here, the paper exhibits more value as a case study than as an application note. The methods section makes up a considerable portion of the paper, and attempts to explain the main ideas underpinning each implementation, and also contains some useful observations about potential pitfalls in each approach. Generally speaking I find it difficult to closely follow algorithms when they are described as prose, and this paper suffers from a lack of clarity in the explanations of each implementation. Figures 2-4 do help with the presentation, though I wonder whether a pseudo-code style presentation might also be helpful?

We thank the reviewer for the suggestion. We now have included pseudo-code presentation in Figure S1-S3

Overall I think the paper will be of most interest to bioinformatics software implementors who are investigating the parallelisation of tools on cloud-based platforms. It is probably of less interest to bioinformatics practitioners, who are interesting in putting the tools into use.

We agree with the reviewer’s comment.

Below are some remarks about the presentation which might improve future revisions:

We really appreciate the reviewer’s careful proofreading and specific corrections!

- (page 2) "achieve maximum performance" -> "achieve high performance" (it is not clear that "maximum" performance is well defined)

Done. See line 26.

- there are numerous instance where the definite article "the" is incorrectly used. For example:
(page 3) "of _the_ powerful computing resources" (should be "of powerful computing resources")
(page 5) "applications of Apache big data platform" (should be "of _the_ Apache big data platform")
(page 21) "More specifically, MapReduce-based schema" (should be "More specifically, _the_ MapReduce-based schema")
I have not listed all examples, and recommend a thorough proof read before final submission.

We have conducted a thorough proof read and corrected similar problems.

- (page 4) "takes advantage of" -> "take advantage of" (because you are talking about two different tools)

Done. See line 55.

- (page 4) "researchers have recently started to embrace distributed systems" -> I don't think this is really true. Distributed systems have been in use in bioinformatics for quite some time now. The trend towards cloud-based and hadoop-styled computations is somewhat more recent, but still not new.

We agree with reviewer’s opinion.
On line 61:
We change it to: “In bioinformatics, researchers have already started to embrace Apache distributed systems to manage and process large amount of high throughput omics data.”

- (page 5) "plenty of" -> "many"

Powered by Editorial Manager® and ProduXion Manager® from Aries Systems Corporation

Done. See line 76.

- (page 5) maybe replace "single-threaded" with "sequential"

Done. See line 226.

- (page 6) it is not clear what "working schemas" means here. Also it might be best to describe what you mean by "schemas" in general, since that is an important concept in the paper.

We are sorry for the confusion and in the abstract part (line 24) add: “However, carefully designed and optimized working flow patterns and execution plans (schemas) are required to take full advantage of the increased computing power while overcoming bottlenecks to achieve high performance.”

- (page 7) "boosts" -> "improves"

Done. See line 114.

- there are numerous instances where pluralisation is done incorrectly. For example:
(page 8) "bottleneck and hotspot can happen" (should be "bottlenecks and hotspots can happen"), and "unbalanced workload" (should be "unbalanced workloads", or "an unbalanced workload")
(page 10) "locations that appears" (should be "locations that appear")
(page 11) "of interests" (should be "of interest")
(page 14) "finishing writings" (should be "finishing writing")
again, a thorough proof reading of the paper before submission should address these issues
- (page 8) "hit" -> "access" (likewise on page 14)
- (page 9) "In a typical WGS" -> "In a typical WGS experiment"
- (page 9) "one of the individual's" -> "one of an individual's"

We have corrected all of these and other places we found.

- (page 10) mentions an "unqualified filter": this concept may not be understood by readers who are not familiar with the VCF format, and therefore should be explained

We agree with the reviewer’s advices and add a brief description (line 163) as: “First, each record is associated with a data quality value in the FILTER column, which records the status of this genomic position passing all filters. Usually only qualified records with a “PASS” filter value are retained.”

- (page 10) "is homozygous reference alleles" -> "is a homozygous reference allele"

Done. See line 169.

- (page 11) "Meantime" -> "Meanwhile"

Done. See line 202.

- (page 11) Hopefully the pre-defined sampling rate is a parameter to your system?

Yes. the sampling rate can be set via the option –r when running the program.

- (page 11) "equal to the reciprocal of input file number": do you mean "equal to the reciprocal of the number of input files"?

We are sorry for the loose description and have corrected it as the reviewer suggested (line 209).

- (page 12) "a partitioner shuffles": it is not clear what the point of shuffling is here. Shuffling suggests that the order is being changed, as in shuffling a deck of cards. Is that is what is happening here? If not, is there a better way to describe this.

Powered by Editorial Manager® and ProduXion Manager® from Aries Systems Corporation

We are sorry for the confusion. Usually a hash-based partitioner shuffles data. Here we use a custom partitioner that keeps the order. So we change shuffle to redirects (line 217).

- (page 13) You use an inconsistent way to number the explanations for each parallelisation technique. For MapReduce, you use "First", "Second". For HBase you use numberings "1) 2)". For Spark you use "Stage I, Stage II". It would be good to be consistent across the manuscript.

We thank the reviewer for the advices. And we change the numbering to make them consistent. We use stage instead of phase in Spark is because stage is a specific term used in Spark literatures for describing groups of steps separated by a data shuffling operation. Other than that, everything is consistent.

- (page 14) "This necessitates the adding of this phase". This sentence seems unusual on its own. Maybe it can be incorporated into the previous sentence, or avoided altogether?

We take the reviewer's advice and incorporate it into the previous sentence (line 246).

- (page 14) "a magnitude faster" -> "and order of magnitude faster"

Done (line 255).

- (page 14) it is not clear what "the normal loading" is.

We are sorry for the confusion.
On line 254
We change it to: "This procedure is therefore at least an order of magnitude faster than the normal loading in which data are loaded sequentially via HBase servers' I/O routines."

- (page 20) "Therefore we are not expecting to see any bottleneck when dealing with even larger scale of data": why not run some much larger tests and report the actual results rather than speculating?

We agree with the reviewer and increase the number of input files to 186. Initially we test on 93 because running cloud cluster is expensive.

- (page 20) In the comparison against a single node implementation, it would be best if you (re)-stated the number of cores used by the single node. Otherwise, saying X-core parallel implementations are Y-times faster is not very helpful. If they use N times as many cores, then we would hope to approach an N times speedup. You could also run some scaling tests on the single-node system.

We thank the reviewer for the suggestion. We added the following for re-state (line 467) the number of cores used by single node: "The single multiway merger is run on a node with the hardware configuration (4 cores and 15G memory) identical to the nodes on which the Apache cluster-based schemas are run."

We have added scaling test on single-node system as described previously, and incorporate the single node cluster of the Apache schemas in the scaling tests.

- (page 22) "We manage to show that all three schemas are _highly_ scalable on both input and data size". I do not think that the reported results warrant the "highly scalable" description for all implementations. The number of cores demonstrated and the size of the input files are not particularly large for modern genomics research. To warrant "highly scalable" would require much larger benchmarks to be reported, and more distinction between strong and weak scaling.

We are sorry for the overstatement and changed "highly scalable" to "scalable" (line 508).

Powered by Editorial Manager® and ProduXion Manager® from Aries Systems Corporation

- (page 27) "grateful for" -> "grateful to"

Done. See line 593.

Reviewer #2:
This paper is about a comparison among three Apache big data platforms, MapReduce, HBase and Spark, to perform sorted merging of massive genome-wide data.

The topic is very important for Bioinformatics, the paper is clear, figures and graphs are easy to read, the scalability is well studied and I appreciate that the code is available for testing.

On the dark side, I read in the introduction that the frameworks were tested using two different tests, while basically only the analysis and integration of VCF files has been tested.

We apologize for the confusion. In the introduction, we mention two tests, one is merging multiple VCF files into one VCF with VCFTools as benchmark (results shown in Table 1), and the other is converting and merging VCF files into a TPED file used by PLINK (results shown in figure 5-10). For the first test, we only compared performance of our schemas with VCFTools without scalability tests because the underlying mechanism is same as merging to TPED file which we show detailed results on scalability. The backbone workflow and execution process of all three schemas remain the same in both applications, the only thing changes is the value content of each key-value pairs. So we expect the scalability properties of first application to be identical to the second one. We add a paragraph (line 170) in the “Data Formats and Operations” section describing the rules of merging VCF files into a single VCF file and reasons why we skip the scalability tests of this application. Also, on the project website at https://github.com/xsun28/CloudMerge/, we provide the source codes and programs for running both applications.

I honestly don't know if this is enough for a publication in such an important journal. Is a single application enough to say what platform is the best for "Sorting and Merging Massive Omics Data"?

In case the title should be changed to specify the single application described.

Otherwise, in order to be more general about omics data, probably at least another test on a completely different bioinformatic application would be necessary to really describe pros and cons of these three different frameworks. I may suggest something about metagenomics, such as:

Zhou, Wei, et al. "MetaSpark: a spark-based distributed processing tool to recruit metagenomic reads to reference genomes." Bioinformatics 33.7 (2017): 1090-1092.

The reason why we used the broad term in the title is because we think the VCF format is representative of many different types of omics data as they can be thought of as collections of genomic regions along the genome. These files are oftentimes need to be merged (and sorted to keep the order). What is more important is that many of these omics data can be represented as a combination of keys and values. This representation allows the reuse of the backbones of our schemas, and users only need to provide their custom functions of generating the contents of their own keys and values, just as what we did in modifying our first application (VCFs to a single TPED) into the second application (VCFs to a single VCF). But we fully agree with the reviewer that omics data are more than just VCF files or collection of genomic regions. So we took the reviewer’s advice and change the title to “… Sorting and Merging Massive VCF files” to narrow down and more accurately reflect the types of data that our methods can be applied to. Although this looks like a single application (a very important one), the sorted merging methods we tackled here can be applied in several other scenarios such as finding union or intersection of multiple ChIP-seq peaks.

Powered by Editorial Manager® and ProduXion Manager® from Aries Systems Corporation

We also added a paragraph in the Introduction about the potential of additional bioinformatics applications and cited the Zhou et al. paper (line 68) as: “The MetaSpark [16] utilized Spark’s distributed data set to recruit large scale of metagenomics reads to reference genomes, achieves better scalability and sensitivity than single-machine based programs.” We did not test our method on metagenomics data since our algorithm is designed to carry out sorted merging, which is not the main computational problem in metagenomics. But we do believe the same principles we demonstrated in this study can be applied to address computational problems encountered in other contexts such as some aspects of the metagenomics analyses that involve the key-value model and sorted merging operations.

Another major point is that no related works are presented. The analysis of massive genotyping datasets in VCF format has been addressed at least by another work, which should be discussed and compared in the paper:

O'Brien, Aidan R., et al. "VariantSpark: population scale clustering of genotype information." BMC genomics 16.1 (2015): 1052.

We thank the reviewer for pointing this out. We now cited this paper and a few related papers. Because the purpose of VariantSpark is to cluster variants efficiently, which is different from the purpose of our tools, hence we are unable to conduct a performance comaprison.

Additionally, we add some other papers about applying Apache distributed systems in bioinformatics and WGS:

On line 62: “For example, the Cancer Genome Atlas project made use of Hadoop framework to split genome data into chunks distributed over the cluster for parallel processing.”

On line 64: Add the Seal and Hadoop-BAM software citations.

On line 74: “Although numerous Apache cluster-based applications have already been developed for processing and analyzing large scale of genomics data including ADAM [1],VariantSpark [20], SparkSeq [21], Halvade [22], SeqHBase [23] among others, we believe there are still many opportunities in biomedical data analyses to take advantage of distributed systems as data becomes larger and more complex.”

We also added a review paper (line 54) on this topic in the Introduction Section.

Al last, I think that there is a bit of confusion in the terminology between Hadoop and MapReduced, which should be solved.

We thank the reviewer for raising this issue. Hadoop is Apache’s implementation of MapReduce Framework on YARN and HDFS. We have clarified this in the manuscript on line 105 and 188.

Reviewer #3:
Authors designed, implemented and evaluated three strategies to perform sorted merging of genomic variant data using distributed processing engines.
They demonstrated that proposed approaches outperform typical single-node solutions (such as VCF-tools), and allows to process larger datasets because of their scalability.
The work improves our knowledge in adapting Big Data tools for genomic variant analysis and provide novel, efficient tools for bioinformatics community.

Major comment: Authors did not mention the problem of dealing with multi-allelic sites (genomic positions with more than 2 different alleles), which is especially important when working with large-scale sequencing data.

We thank the reviewer for the advices, we add to the discussion (line 538): “Our

Powered by Editorial Manager® and ProduXion Manager® from Aries Systems Corporation

	implementations automatically take care of multi-allelic positions which are frequent in large scale VCF flies by retaining the information of all alleles until the merging actually occurs."<br><br>Minor comment: Please, synchronized formatting of subsections.<br><br>We thank the reviewer for pointing it out, and we have synchronized formatting of subsections.
**Additional Information:**	
**Question**	**Response**
Are you submitting this manuscript to a special series or article collection?	No
**Experimental design and statistics**<br><br>Full details of the experimental design and statistical methods used should be given in the Methods section, as detailed in our Minimum Standards Reporting Checklist. Information essential to interpreting the data presented should be made available in the figure legends.<br><br>Have you included all the information requested in your manuscript?	Yes
**Resources**<br><br>A description of all resources used, including antibodies, cell lines, animals and software tools, with enough information to allow them to be uniquely identified, should be included in the Methods section. Authors are strongly encouraged to cite Research Resource Identifiers (RRIDs) for antibodies, model organisms and tools, where possible.<br><br>Have you included the information requested as detailed in our Minimum Standards Reporting Checklist?	Yes
**Availability of data and materials**<br><br>All datasets and code on which the conclusions of the paper rely must be either included in your submission or deposited in publicly available repositories (where available and ethically appropriate), referencing such data using a unique identifier in the references and in the "Availability of Data and Materials" section of your manuscript.	Yes

Have you have met the above requirement as detailed in our Minimum Standards Reporting Checklist?	

Powered by Editorial Manager® and ProduXion Manager® from Aries Systems Corporation

Revised Manuscript

Click here to download Manuscript reviewedmanuscriptSubmission.docx

Optimized Distributed Systems Achieve Significant Performance Improvement on Sorted Merging of Massive VCF Files

Xiaobo Sun[1], Jingjing Gao[2], Peng Jin[3], Fusheng Wang[4*], Zhaohui Qin[2,5*]

[1]Department of Computer Sciences, Emory University, Atlanta, GA 30322, USA.

[2]Department of Medical Informatics, Emory University School of medicine, Atlanta, GA 30322, USA.

[3]Department of Human Genetics, Emory University School of Medicine, Atlanta, GA 30322, USA.

[4]Department of Biomedical Informatics, Stony Brook University, Stony Brook, NY 11794, USA.

[5]Department of Biostatistics, Emory University, Atlanta, GA 30322, USA.

X.S. Email: xsun28@emory.edu

J.G. Email: jingjing.gao@abbvie.com

P.J. Email: peng.jin@emory.edu

F.W. Email: fusheng.wang@stonybrook.edu

Z.Q. Email: zhaohui.qin@emory.edu

*Correspondence: zhaohui.qin@emory.edu, fusheng.wang@stonybrook.edu

Abstract

Background: Sorted merging of genomic data is a common data operation necessary in many sequencing-based studies. It involves sorting and merging genomic data from different subjects by genomic locations. In particular, merging a large number of Variant Call Format (VCF) files are frequently encountered in large scale whole genome sequencing or whole exome sequencing projects. Traditional single machine based methods become increasingly inefficient when processing hundreds or even thousands of VCF files due to the excessive computation time and I/O bottleneck. The distributed systems and the more recent cloud-based systems offer an attractive solution. However, carefully designed and optimized working flow patterns and execution plans (schemas) are required to take full advantage of the increased computing power while overcoming bottlenecks to achieve high performance.

Findings: In this study, we custom design optimized schemas for three Apache big data platforms, Hadoop (MapReduce), HBase and Spark, to perform sorted merging of large number of VCF files. These schemas all adopt the divide-and-conquer strategy to split the merging job into sequential phases/stages consisting of subtasks which are conquered in an ordered, parallel and bottleneck-

free way. In two illustrating examples, we test the performance of our schemas on merging multiple VCF files into either a single TPED or VCF file, which are benchmarked with the traditional single/parallel multiway-merge methods, message passing interface (MPI) based high performance computing (HPC) implementation and the popular VCFTools.

Conclusions: Our experiments suggest that all three schemas either deliver a significant improvement in efficiency or render much better strong/weak scalabilities over traditional methods. We believe that our findings provide generalized scalable schemas for performing sorted merging on genetics and genomics data using these Apache distributed systems.

Keywords: Sorted merging, whole genome sequencing, MapReduce, Hadoop, HBase, Spark.

Findings

Introduction

With the rapid development of high-throughput biotechnologies, genetics studies have entered the Big Data era. Studies like Genome Wide Association Studies (GWASs), Whole Genome Sequencing (WGS) and whole exome sequencing (WES) studies have produced a massive amount

of data. The ability to efficiently manage and process such massive data becomes increasingly important in a successful large scale genetics study [1-3]. Single machine based methods are inefficient when processing such big data due to the prohibitive computation time, I/O bottleneck, and CPU and memory limitations. Traditional HPC techniques based on MPI/OpenMP also suffer from limitations such as not allowing addition of computing nodes at runtime, shortage of a fault-tolerant and high available file system, inflexibility of customizing the computing environment without administrator permission of the cluster [3, 4]. It becomes increasingly attractive for investigators to take advantage of more powerful distributed computing resources or the cloud to perform data processing and analyses [3, 5]. Apache Foundation has been a leading force in this endeavor and has developed multiple platforms and systems including Hadoop [6, 7], HBase [8] and Spark [9]. All these three Apache platforms have gained substantial popularity in recent years, and have been endorsed and supported by major vendors such as Amazon Web Services (AWS).

In bioinformatics, researchers have already started to embrace Apache distributed systems to manage and process large amount of high throughput omics data. For example, the Cancer

Genome Atlas project made use of the Hadoop framework to split genome data into chunks distributed over the cluster for parallel processing[3, 10]. The CloudBurst [11], Seal [12], Hadoop-BAM [13] and Crossbow software [14] took advantage of the Hadoop framework to accelerate sequencing read mapping, aligning and manipulations as well as SNP calling. The Collaborative Genomic Data Model (CGDM) [15] adopted HBase to boost the querying speed for the main classes of queries on genomic databases. The MetaSpark [16] utilized Spark’s distributed data set to recruit large scale of metagenomics reads to reference genomes, achieves better scalability and sensitivity than single-machine based programs [17]. Industry cloud computing vendors such as Amazon [18] and Google [19] are also beginning to provide specialized environments to ease genomics data processing in the cloud.

Although numerous Apache cluster-based applications have already been developed for processing and analyzing large scale of genomics data including ADAM [1],VariantSpark [20], SparkSeq [21], Halvade [22], SeqHBase [23] among others, we believe there are still many opportunities in biomedical data analyses to take advantage of distributed systems as data

becomes larger and more complex. One particular example is sorted merging, which is a ubiquitous operation in processing genetics and genomics data. As an example, in WGS, variants identified from individuals are often called and stored in separate Variant Call Format (VCF) files. Eventually these VCF files need to be merged (into a VCF or TPED file) as required by downstream analysis tools such as PLINK [24] and BlueSNP [25, 26]. Either a VCF or TPED file requires data to be sorted by genomic locations, thus these tasks are equivalent to the well-known sorted full-outer-joining problem [27, 28]. Currently, they are handled by software such as VCFTools [29] and PLINK, which become very cumbersome even in the face of a moderate number of VCF files. The main reason is that these tools adopt the multiway-merge-like method [30] with a priority queue as the underlying data structure to ensure the output order. Although such a method only requires one round of read through of the input files, a key deficiency is that it can only have one consumer to access items from the data queue, which literally makes it sequential on writing. This problem cannot be eliminated even if the multiway-merging is implemented as parallel processes due to I/O saturation, workload imbalance among computing units, and memory limitation. Therefore, these single-machine based tools are inefficient and time-consuming when handling large datasets.

In this study, we use the case of sorted-merging multiple VCF files to demonstrate the benefits of using Apache distributed platforms. However, simply running sorted merging on such distributed systems runs into problems of bottlenecks, hotspots and unordered results commonly seen in parallel computations. Rather, we believe working schemas custom designed for each specific distributed platform are required to unleash their full potentials. To overcome the limitations of single-machine, traditional parallel/distributed, and simple Apache distributed system based methods, we propose and implement three schemas running on Hadoop, Spark and HBase respectively. We choose these three platforms because they represent cloud distributed systems providing data partitioning based parallelism with distributed storage, data partitioning based parallelism with in-memory based processing, and high dimensional table like distributed storage, respectively. Hadoop [6] is the open source implementation of MapReduce [7] based parallel key-value processing technique, and has the advantage of transparency and simplicity. HBase [8] is a data warehousing platform which adopts Google's BigTable data storing structure [31] to achieve high efficiency in storing and reading/writing large scale of sparse data. Spark [9] introduces the

concept of Resilient Distributed Dataset (RDD) and Directed Acyclic Graph (DAG) execution to parallel key-value processing, thus enabling fast, robust and repetitive in-memory data manipulations. Specifically, our schemas involve dividing the job into multiple phases corresponding to tasks of loading, mapping, filtering, sampling, partitioning, shuffling, merging and outputting. Within each phase, data and tasks are evenly distributed across the cluster, enabling processing large scale of data in a parallel and scalable manner, which in turn improves both speed and scalability.

Methods

Overview

The benefits of using the three Apache distributed platforms to perform sorted merging are four-fold when compared to using the multiway-merge method [30], a relational database based approach, or a HPC framework. First, with genomic locations as keys and genotypes as values, it is readily transformed into the key-value model in which all three platforms offer a rich set of parallel operations. Second, data in VCF files are semi-structured. This type of data ideally fit for

the three platforms which allow defining the schema during data loading, avoiding the preprocessing of raw data into a rigid schema as in a relational database. Third, all these platforms provide built-in efficient task coordination, high fault tolerance, data availability and locality which are absent in the traditional HPC framework. Fourth, the merged results are directly saved onto a distributed file system such as HDFS or Amazon S3 which can be directly used for subsequent cluster-based GWAS or WGS analytical tools such as BlueSNP.

Despite these advantages, simply performing sorted merging on these Apache distributed systems will not deliver expected results for the following reasons. First, it can lead to globally unsorted results. Hash-based shuffling of input data is the default mechanism for distributing data to parallel working units in the system. However, shuffling will lead to globally unsorted results. Second, bottlenecks and hotspots can happen during the processing in the cluster. Bypassing the hashing based shuffling can lead to unbalanced workloads across the cluster, result in straggling computing units which become the bottlenecks for response time. In addition, for parallel loading of presorted data into HBase, data being loaded from all the loading tasks access the same node simultaneously while other nodes are idling, leading to an I/O hotspot. Third, sampling costs could

become prohibitive. Although Hadoop provides a built-in utility named *total-order-merging* [27] to achieve both workload balance and global order, it involves transferring to and sampling all the data on a single node. The communication costs over the network and disk I/O can be prohibitive when data size is very large. In the following sections, we will illustrate how our custom designed schemas are able to overcome these limitations in detail.

Data Formats and Operations

In a typical WGS experiment, data analysis often starts from individual genotype files in the VCF format [32]. A VCF file contains data arranged into a table consisting of eight mandatory fields including chromosome (CHROM), the genomic coordinate of the start of the variant (POS), the reference allele (REF), a comma separated list of alternate alleles (ALT), among others. In our experiments, we use a dataset consisting of the VCF files of 186 individuals [33] generated from Illumina's BaseSpace software (Left tables in Figure 1). Each VCF file has around 4-5 million rows, each row contains information on one of the individual's genomic variants. Each VCF file is about 300 megabyte in size. In an attempt to protect the privacy of the study subjects, we apply the following strategy to conceal their real genetic variant information contained in the VCF files: we

first transform each original genomic location by multiplying it with an undisclosed constant real number, taking the floor integer of the result, and then add another undisclosed constant integer number.

It is common that multiple VCF files need to be merged into a single TPED file for analysis tools such as PLINK. A TPED file resembles a big table, aggregating genotypes of all individuals under investigation by genomic locations (right table in Figure 1). The merging follows several rules. First, each record is associated with a data quality value in the FILTER column, which records the status of this genomic position passing all filters. Usually only qualified records with a “PASS” filter value are retained. Second, genotypes in VCF files are stored in the form of allele values, where 0 stands for the reference allele, 1 stands for the first mutant allele, 2 stands for the second mutant allele, and so on. Allele values must be translated into corresponding types of nucleotides in the TPED file. Third, all individuals need to have a genotype for genomic locations that appear in at least one VCF file. The default genotype for a missing value is a pair of homozygous reference alleles. The merging of multiple VCF files to a single VCF file follows the rules as: First, the ALT and INFO columns of a genomic location in the merged file are set as the

concatenated values of the corresponding columns on that location from all input files with duplicated values removed. Second, the QUAL column of a genomic location in the merged file is set as a weight-averaged quality value of all individuals on that location. Third, a genomic location is kept only when it appears in at least one input file and has a FILTER column value that equals to “PASS”. Fourth, if an individual does not have allele values on a genomic location in the input file, their missing allele values are designated as “.” in the merged file.

For our Apache cluster-based schemas, the merging of multiple VCF files into a single TPED file and the merging of multiple VCF files into a single VCF file differ only in the value contents of the key-value pairs, so they should have the same scalability property. Although we implement the applications of both merging types using our Apache cluster-based schemas, which are available on our project website, we focus our experiments on the merging of multiple VCF files into a single TPED file and only evaluate the execution speed of the merging of multiple VCF files into a single VCF file with VCFTools as the benchmark.

MapReduce (Hadoop) Schema

This schema is built on Hadoop's underlying model MapReduce and running on Hadoop clusters. MapReduce [7] is a parallel computing model based on a *split-apply-combine* strategy for data analysis, in which data are mapped to key-values for splitting (mapping), shuffling and combining (reducing) for final results. We use Apache Hadoop-2.7 as the system for our implementation. Our optimized schema consists of two MapReduce phases, as shown in Figure 2 (the pseudocodes are shown in Figure S1).

1) First MapReduce phase.

Raw data are loaded from HDFS into parallel mappers to perform the following tasks: First, unqualified data are filtered out and qualified ones are mapped to key-value pairs. The mapper output key is the genomic location and output value is the genotype and individual ID. Second, Key-value pairs are grouped together by chromosomes and temporarily saved as compressed Hadoop sequence files [34] for faster I/O in the second MapReduce phase. With this grouping, we only need to merge records from selected chromosomes of interest rather than from all of them. Meanwhile, these records are sampled to explore their distribution profile of keys along chromosomes to determine boundaries. The boundaries are determined such that there is an

approximately equal number of records within each segment. Because all records falling in the same segment will be assigned to the same reducer in the later phase, boundaries calculated in this way ensure that the workload of each reducer is balanced. There are two rounds of samplings. The first one happens in each mapper with a pre-specified sampling rate, which in our case is set to be 0.0001. Sampled records are then separated and distributed to different reducers in this phase by chromosomes, where they are sampled again with a rate equal to the reciprocal of the number of input files. This second sampling effectively limits the number of final sampled records even in the face of a very large number of input files. Because the number of reducers instantiated in the second phase is decided by the number of sampled records, we can therefore avoid launching unnecessary reducers thus reducing task overheads.

2) Second MapReduce phase.

In this phase, multiple parallel MapReduce jobs are created, one for each chromosome, to handle all the records in sequence files generated from the first phase. Within each job, a partitioner redirects records to the appropriate reducer by referring to the splitting boundaries from the previous phase, so that records falling in between the same pair of boundaries are aggregated

together. Finally, each reducer sorts and merges aggregated records by genomic locations before saving them to a TPED file. In this way, globally sorted merging can be fulfilled.

HBase Schema

HBase [8] is a column-oriented database where data are grouped into column families and split horizontally into regions spreading across the cluster. With this data storing structure, it supports efficient sequential reading and writing of large-scale data as well as fast random data accessing. Also, HBase is storage efficient because it can remember null values without saving them on disk. These features make HBase an ideal platform for managing large, sparse data with relatively low latency which naturally fits the sorted merging case. We use the HBase-1.3 as the system for our implementation. As shown in Figure 3, our optimized HBase schema is divided into three phases as discussed next (refer to Figure S2 for pseudocodes).

1) Sampling phase

The main challenge of HBase is to avoid computational hotspot in the cluster which can happen when it starts loading a table from a single region hosted by a single node. Therefore, we need to

presplit the table into regions of approximately equal size before loading. The sampling phase is introduced to determine reasonable presplitting regional boundaries. The total number of regions is set to be half of the number of input files so that the size of each region is approximately 1GB. Meanwhile, mappers of this phase also save qualified records as compressed Hadoop sequence files on HDFS which are used as inputs in the next phase. In addition, filtering and key-value mapping also take place in this phase.

2) Bulk loading phase

Even when the table has been presplit evenly, the hotspot problem of loading sorted inputs can still emerge because sorted records are loaded sequentially and at any instant they still access the same region and server, which necessitates the adding of this phase. During the bulk loading, the key and value of each record produced from the previous phase is converted into HBase's binary row-key and column-value respectively, and saved into a HFile, HBase's native storage format. The row-key here is in the form of chromosome-genomic location, and column-value refers to reference allele, individual ID and genotype. The bulk loading populates each HFile with records falling in the same pair of presplit regional boundaries. Because HFiles are written simultaneously

by parallel mappers/reducers, all working nodes are actively involved and the regional hotspot is thus circumvented. Upon finishing writings, the HBase can readily load HFiles in parallel into the table by simply moving them into local HBase storage folders. This procedure is therefore at least an order of magnitude faster than the normal loading in which data are loaded sequentially via HBase servers' I/O routines. The order of records in the table is guaranteed because they are internally sorted by writing reducers and HBase's Log-Structured Merge-tree [35]. It is noteworthy to mention that VCF records are sparse, thus HBase is very storage-efficient.

3) Exporting phase

A scan of a specified genomic window is performed on the table. It involves launching parallel mappers each receiving records from a single HBase region, filling in missing genotypes, concatenating records with the same row-key, and outputting final results into TPED files.

Spark Schema

Spark [9] is a distributed engine that embraces the ideas of MapReduce and RDD. It can save intermediate results in the form of RDD in memory, and perform computations on them. Also, its

computations are lazily evaluated, which means the execution plan can be optimized since it tries to include as many computational steps as possible. As a result, it is ideal for iterative computations such as sorted merging. We implement our optimized Spark schema on Spark-2.1. It has three stages which we describe below and present in Figure 4 (refer to Figure S3 for pseudocodes).

1) RDD preprocessing stage

This stage involves loading raw data as RDDs, filtering, and mapping RDDs to paired-RDDs with keys (chromosome and genomic position) and values (reference allele, sample ID and genotype). This stage ends with a sorting-by-key action which extends to the next stage.

2) Sorting and merging stage

The sort-by-key shuffling repartitions and sorts PairRDD records so that records with the same key are aggregated together, which are then merged into the TPED format and converted back to RDD records for outputting. However, Spark's native family of group-by-key functions for merging should not be used here because their default partitioner is hash-based and different from

the range-based partitioner used by previous sort-by-key function. Consequently, the merged results would be reshuffled into an unsorted status. We therefore optimize the merging in order to bypass these functions such that merging can be performed locally without data reshuffling to ensure both order and high speed.

3) Exporting stage

In this stage, merged RDD records are saved as TPED files on HDFS.

Execution parallelism has an important impact on the performance. To maximize performance, the number of parallel tasks is set to be the number of input files. In this way, the data locality is maximized and each task is assigned a proper amount of work. In addition, unlike using MapReduce or HBase, when performing sorting by keys, no explicit sampling is needed because Spark keeps track of the number of records before determining repartition boundaries.

Parallel Multiway-Merge and MPI-based High Performance Computing Implementations

For most bioinformatics researchers, their daily working environment is still traditional in-house

HPC clusters or stand-alone powerful servers (with cores ≥ 16 and memory ≥ 200G) rather than heterogeneous cloud-based clusters. Therefore we also implement a parallel multiway-merge program running on a single machine and a MPI-based (mpi4py v3.0) "single program, multiple data (SPMD)" program running on a HPC cluster as benchmarks. The source codes are available at https://github.com/xsun28/CloudMerge (CloudMerge; RRID: SCR_016051). We choose to implement multiway-merge, because many existing bioinformatics tools, including VCFTools and PLINK, adopt it as the underlying algorithm for sorted merging. Multiway-merge is highly efficient on single machine as it requires only one scan of sorted input files, so it can theoretically run at the speed of disk I/O.

Generally, there are two types of parallelism---data parallelism and task parallelism. The former splits data horizontally into data blocks of roughly equal sizes (the size of genomic intervals in our case) before assigning them to all available processes; the latter assigns a roughly equal number of input files to each process. For parallel multiway-merge, we choose data parallelism because the implementation of task parallelism would be the same as the HPC-based implementation running on a single node. Perhaps the most difficult part of data parallelism is that we do not know the data

distribution across all input files, which usually leads to the problem of workload imbalance among processes. If we pre-sample all the input files to estimate the record distribution, then a full scan of the input files is required which will almost certainly takes more time than the single-process multiway-merge method. As a compromise, we assume that the distributions of SNP locations in all VCF files are uniform and the input files can be split into regions of approximately equal sizes. The total number of regions are set to be the number of concurrent processes so that each region is specifically handled by a process. To avoid seeking of a process's file reader to its starting offset from the beginning of the file, we take advantage of the Tabix indexer [36], which builds indices on data blocks of the input file and place the reader's pointer directly onto the desired offset. One important aspect of the Tabix indexer is that it requires the input file to be compressed in bgzip format which is not supported by Hadoop, HBase or Spark. The compression and decompression of a file in bgzip format can be much faster than in bz2 format used in our cluster-based schemas, single multiway-merge and HPC-based implementations, so parallel multiway-merge can run much faster than other methods/schemas when input data size is small.

For the HPC-based implementation, we adopt the task parallelism (Figure 5) to avoid sampling and workload imbalance. Otherwise the workflow of HPC-based implementation is the same as that of the MapReduce-based schema with the same operations and the same order: sampling in parallel, dividing the dataset into splits of equal sizes, and assigning the splits to processes to perform the merging. But this implementation is without data locality offered by HDFS and task coordination offered by YARN and thus has a performance no better than the MapReduce-based schema. Specifically, input files are shared across all nodes in the cluster via a Network File System (NFS). In the first round, each core/process fetches roughly the same number of files from the NFS and performs multiway-merging locally. In the following rounds, we adopt a tree-structured execution strategy. In the second round, processes with even ID numbers (process id starts from 0) retrieve the merged file from its adjacent process to the right, which are then merged with its local merged file. Processes with odd ID number are terminated. In the third round, processes with ID divisible by four retrieve the merged file from its adjacent process to the right in the second round to merge with its local merged file. This process continues until all the files are merged into a single file for a total of log(n) rounds, where n is the number of the input files.

Strong and Weak Scalabilities

In this study, we quantify scalability by measuring computing efficiency in tests of strong and weak scalabilities. We define efficiency as the average time cost of processing a file per core:

$$\text{Efficiency} = (T_b*C_b/N_b) / (T_i*C_i/N_i)$$

where T_b is the baseline running time, C_b is the baseline number of cores, N_b is the baseline number of input files, T_i is the current running time, C_i is the current number of cores, N_i is the current number of input files. We also incorporate the parallel multiway-merge and MPI-based HPC implementations as benchmarks in the tests.

For the strong scalability test, we fix the number of input files at 93 and increase the computing resources up to 16-fold from the baseline. The baseline is a single node (4 cores) for all methods/schemas except for the parallel multiway-merge in which only a single core is used because it can only run on a single machine. For the weak scalability test, we increase both computing resources and input data size at the same pace. The ratio is ten file/core for parallel multiway-merge and ten file/node for all others.

Results

We conduct experiments of Apache cluster-based schemas using Amazon's Elastic MapReduce (EMR) service and experiments of the HPC-based implementation using MIT's StarCluster^{TM} toolkit which launches an AWS openMP virtual private cluster (VPC). Within both infrastructures, we choose EC2 working nodes of m3.xlarge type, which has four High Frequency Intel Xeon E5-2670 v2 (Ivy Bridge) Processors and 15GB memory. We conduct experiments of parallel multiway-merge on a single EC2 r4.8xlarge instance with 32 High Frequency Intel Xeon E5-2686 v4 (Broadwell) processors and 244 GB memory. We use a dataset consisting of 186 VCF files [33] generated from Illumina's BaseSpace software.

Overall Performance Analysis of Clustered-based Schemas

Our primary goal is to explore the scalabilities of the three schemas on input data size and available computing resources, namely CPUs. To achieve this, in this experiment we adjust the number of input files from 10 to 186, with an approximate total uncompressed size from 2.5 G to 40 G, and use a varying number of working nodes from 3 to 18, namely 12 to 72 cores.

As Figure 6 shows, for all three schemas, given a fixed number of cores, the execution time increases at a slower pace than that of the input data size. On the one hand, the increase of execution time is more obvious with fewer cores because each core is fully utilized. As the number of input files increases, so does the number of parallel tasks assigned to each core. For example, given 12 cores, as the number of input files increases from 10 to 186 (18.6 fold), the execution time increases from 739 to 4,366 seconds (~5.9 fold) for the MapReduce schema, from 375 to 5,479 seconds (~14.6 fold) for the HBase schema, and from 361 to 1,699 seconds (~4.7 fold) for the Spark schema. On the other hand, with relatively more cores such as 72, this linear increasing trend is less pronounced because there are more cores than tasks so that all cores are assigned at most one task. We also notice that when input data size is small or moderate, the Spark schema does not always show a consistent improvement in terms of execution time when using more cores. This is reflected, for example, in the intersection of curves occurred between 24 and 72 cores in Figure 6c. This phenomenon is attributed to the limitation of Spark's internal task assignment policy which gives rise to the possibility that some nodes are assigned more than one tasks while others remain idle.

Comparing Strong and Weak Scalabilities between Apache Cluster-based Schemas and Traditional Parallel Methods

Figure 7 shows the results of the strong scalability. In accordance with the Amdahl's law [37], all schemas/methods show degraded efficiency with increasing computing nodes/cores. Parallel multiway-merge has the steepest degradation because the more parallel processes, the higher likelihood of workload imbalances among them. In addition, disk I/O reaches saturation as more processes write simultaneously. Furthermore, to achieve data parallelism and improve execution speed, we use Tabix indexer to index data blocks of input files. While reading, each process needs to maintain a full copy of file descriptors, indices and uncompressed current data blocks of all input files in memory. When both the number of processes and input files are large, great pressure is placed on the memory management. For instance a test with 93 files and 16 processes requires over 100GB memory, which results in a very long memory swap and garbage collection (GC) time. In contrast, the MapReduce-based schema has the best efficiency. Surprisingly, its efficiency even improves when the number of cores doubles from the baseline. This is because it has many parallel tasks in its second MapReduce phase, and when the core allowance is low, the overheads of repetitive task launching and terminating on a single core become non-negligible.

Consequently, as the number of cores starts to increase, the actual proportion of overheads in the total running time decreases, leading to an improved efficiency. Nonetheless, as the number of cores further increases, the unparalleled parts of the schema gradually dominate the total running time, leading to a reduced efficiency eventually.

For the weak scalability test (Figure 8), following Gustafson's law [38], all methods/schemas show a much better efficiency than in the strong scalability test. Meanwhile, for the same reasons as the strong scalability, the MapReduce-based schema enjoys the best efficiency while the HPC-based implementation has the worst. This is because, for the HPC-based implementation, as the number of input files increases, the total number of merging rounds also increases, leading to a significantly reduced efficiency. Finally, all three Apache cluster-based schemas demonstrate significantly better weak scalability than the two traditional parallel methods.

The Anatomic Performances Analysis of Apache Cluster-based Schemas

Another important goal of our study is to identify potential performance bottlenecks, so we evaluate the execution time of each phase/stage of all three schemas. Figure 9 shows the trends of

the anatomic computing time spent on merging increasing number of VCF files (from 10 to 186) using 48 cores. For the MapReduce schema (Figure 9a), its two phases account for a comparable proportion of total time and both show a linear or sublinear scalability. The reason that the time cost of the first phase between 40 and 93 input files remains flat is because both runs use two rounds of mappers. As the number of files doubles to 186, four rounds of mappers are required which results in about a two-fold increase in the time cost as expected. For the three phases of the HBase schema (Figure 9b), they are scalable with input data size. Meanwhile, the second phase becomes more dominant with more input files owing to the larger amount of shuffled data during the writing of HFiles. However, we do not consider it as a bottleneck since all tasks of this phase are parallelized with no workload or computational hotspot. We do not observe a super-linear (relative to input data size) increment pattern from the figure neither. Finally, Figure 9c shows the time costs of the three stages of the Spark schema. They show a uniform increasing trend with the number of input files. Among them, the second stage takes up a considerable proportion of the total execution time as it has a relatively expensive sort-by-key shuffling operation. Although no data is shuffled in the first stage, its time lapse is close to that of the second stage. This is because at the end of the first stage, data are sampled to determine the boundaries used by sort-by-key's

range partitioner. This operation demands a considerable execution time because it scans all the data and balances them if necessary.

Given that no super-linear increasing trend is observed in running time for all phases/stages of the three schemas and they generally scale well with the input data size, we reach the conclusion that although the performances of these schemas might degrade to some extent when dealing with even larger input data due to overheads such as data transmission over network, we do not expect to see any significant bottleneck.

Comparing Execution Speed between Apache Cluster-based Schemas and Traditional Methods

Another intriguing question is: how does the speed of the Apache cluster-based schemas compare to single machine based and traditional parallel/distributed methods/applications on merging multiple VCF files into a single VCF or TPED file? To answer this question, we choose the widely-used VCFTools (v4.2) and a single-process multiway-merge implementation as single-process benchmarks and parallel multiway-merge and HPC-based implementations as

parallel/distributed benchmarks, which are the same ones used in the experiments of strong and weak scalabilities shown above.

In the first experiment, we merge 40 VCF files into one VCF file using VCFTools as the benchmark. As shown in Table 2, VCFTools takes 30,189 seconds while the fastest Apache cluster-based schema among the three, the MapReduce-based, takes only 484 seconds using 72 cores, which is about 62-fold faster. In the second experiment (Figure 10), we test the time costs of merging of multiple VCF files into a single TPED file using single/parallel multiway-merge and HPC-based implementations as benchmarks. The single multiway merger is run on a node with the hardware configuration (4 cores and 15G memory) identical to the nodes on which the Apache cluster-based schemas are run. The parallel multiway merger is run on a node with a maximum of 18 simultaneously running processes. The HPC-based implementation is run on an 18-node cluster with the same hardware configuration as the cluster where the Apache cluster-based schemas are run. Initially, with ten input files, the parallel multiway-merge (~30 seconds) is much faster than all the other methods; it is about 7.3-fold faster than the fastest Apache cluster-based schema (MapReduce, 221 seconds). On the other hand, the slowest method is the single-process multiway

merger which takes 620 seconds to finish and is about 2.8-fold slower than the MapReduce-based schema. It is worth mentioning that, in this test the parallel multiway-merge is essentially the same as the single-process multiway-merge, and the speed difference (~378 seconds) between them is the result of a different compression format (bz2 vs bgzip) of the input files as explained above. As we gradually increase the number of input files to 186, the difference in speed between the fastest overall method (parallel multiway merger, 602 seconds) and the fastest Apache cluster-based schema (MapReduce, 809 seconds) reduces to about 1.3-fold, while the difference between the slowest overall method (single multiway merger, 13,219 seconds) and the MapReduce-based schema increases to 16.3-fold. In addition, all three Apache schemas significantly outperform the HPC-based implementation. As explained in the strong and weak scalabilities section, we expect that the larger the input data size, the faster the Apache cluster-based schemas run compared to the other traditional methods.

We also compare the time cost among the three schemas (Figure 10). It turns out that they have a comparable speed. More specifically, the MapReduce schema performs the best if enough cores are available and the input data size is large; the HBase schema performs the best with moderate

input data size; the Spark schema performs the best if only a limited number of cores are available and the input data size is large. The rationale behind our observation is that, when the number of cores is sufficient, the MapReduce-based schema can make the most use of the available computing resources because it runs a constant 25 parallel jobs (one for each of chromosomes 1-22, X Y and M (Mitochondria)) in its second phase. In contrast, the Spark-based schema has fewer tasks whose number equals to the number of input files in order to achieve maximum data-task locality. When the input data size is moderate, the HBase-schema triumphs due to its internal sorting and relative compact storage format of intermediate data. When the input data size is large and computing resource is relatively limited, the Spark-based schema outperforms the other two owing to its least number of data shuffling (only one), execution plan optimization, and ability to cache intermediate results in memory. We caution that the computing time may fluctuate depending on the distribution of genomic locations in the input files as well as the data loading balance of the HDFS.

Discussion

In this report, we describe three cluster-based schemas running on the Apache Hadoop

(MapReduce), HBase and Spark platforms respectively for performing sorted merging of variants identified from WGS. We show that all three schemas are scalable on both input data size and computing resources, suggesting that large scale of omics data can be merged efficiently given the computing resources that are readily available in the cloud. Furthermore, the three schemas show better strong and weak scalabilities than traditional single machine-based parallel multiway-merge and cluster-based HPC methods owing to the absence of I/O bottleneck, better workload balance among nodes, less pressure on memory, as well as data locality and efficient task coordination mechanisms provided by HDFS and YARN. We also show that even with a moderate-sized cluster and input data, all three schemas significantly outperform the broadly-used, single-machine based VCFTools, single-process multiway-merge and HPC-based implementations. Although initially the parallel multiway-merge implementation is much faster than the Apache schemas owing to its advantage of local I/O and light compression of input files, its poor scalability diminishes its initial advantage as the number of concurrent processes and input files increases. Consequently, we expect that the Apache cluster-based schemas eventually outperform the parallel multiway-merge when merging a much larger scale of data using a larger number of cores.

Unlike normal merging, efficient sorted merging of many large tables has always been a difficult problem in the field of data management. Multiway-merge is the most efficient single-machine based method for sorted merging, but its performance is limited by the disk I/O [39]. Sorted merging also places challenges to distributed system based solutions because neither the efficient hash-based merging nor caching the intermediate table in shared memory is feasible [40]. Although a utility named *total-order-joining* is provided by the Hadoop for addressing this problem, it suffers from both network communication and local disk I/O bottlenecks, thus is not scalable [27, 41]. In contrast, our schemas divide this problem into different phases/stages of tasks each conquered in parallel to bypass these bottlenecks and achieve maximum parallelism. Furthermore, in addition to merging sequencing variant data, the schemas can be generalized for other key-based, sorted merging problems that are frequently encountered in genetics and genomics data processing. As an example, they can be slightly modified to merge multiple BED format files such as ChIP-seq peak lists [42] and other genomic regions of interest. Other potentially useful features include: 1) Unlike traditional sorted merging algorithms which usually

require presorted inputs for a better performance, our schemas are free of such a requirement; 2) Our implementations automatically take care of multi-allelic positions which are frequent in large scale VCF flies by retaining the information of all alleles until the merging actually occurs.

Finally, in light of different features and specialties of the three platforms, each of the three schemas we developed has its own advantages and disadvantages under different application scenarios as summarized in Table 1. For example, the MapReduce schema is good for a static one-time, non-incremental merging on large-size data provided sufficient cores are available since it has the most parallel jobs, the least overheads, and the most transparent workflow. The HBase schema, supported by data warehousing technologies, fits for an incremental merging since it does not need to re-merge existing results with new ones from the scratch only if the incremental merging is performed on the same chromosomes. Also, it provides a highly-efficient storage and On-Line Analytical Processing (OLAP) on merged results. The Spark schema is ideal for merging large scale of data with relatively limited computing resources because it has the least data shuffling and keeps intermediate results in memory. A bonus brought by Spark is that subsequent statistical analyses can be carried out directly on the merged results using its rich set of parallel

statistical utilities.

Availability and Requirements

Project name: CloudMerge

Project home page: https://github.com/xsun28/CloudMerge

Operating system(s): Linux

Programming language: Java, Python

Other requirements: Java 1.7 or higher, Python 2.7 or 3.6, Hadoop-2.7, HBase-1.3, Spark-2.1, StarCluster 0.95, MPI for Python 3.0.0

License: Apache License 2.0

Availability of Data and Materials

The source codes of the project are available in GitHub. The 186 individual VCF files used in our experiments are modified from the original VCF files obtained from WGS conducted by the Consortium on Asthma among African-ancestry Population in the Americas (CAAPA) [33]. To

conceal the potential individual identifiable genotype information from the public, we encrypt the authentic genomic location of the original 93 VCF files to generate a new batch of encrypted VCF files. Please refer to *Data Formats and Operations* section for details. The encrypted VCF files are available on AWS S3 at https://s3.amazonaws.com/xsun316/encrypted/encrypted.tar.gz. We also provide sample results of merging 93VCF files into either one VCF or one TPED file using our Apache cluster-based schemas, which are available on AWS S3 at https://s3.amazonaws.com/xsun316/sample_results/result.tar.gz.

Abbreviations

VCF: Variant Call Format; MPI: Message Passing Interface; HPC: High Performance Computing; GWAS: Genome Wide Association Studies; WGS: Whole Genome Sequencing; WES: whole exome sequencing; AWS: Amazon Web Service; CGDM: Collaborative Genomic Data Model; SAM/BAM: Sequence/Binary Alignment/Map; RDD: Resilient Distributed Dataset; DAG: Directed Acyclic Graph; SPMD: Single Program, Multiple Data; NFS: Network File System; EMR: Elastic-MapReduce; VPC: Virtual Private Cluster; GC: Garbage Collection; CAAPA: Consortium on Asthma among African-ancestry Population in the Americas;

Consent for Publication

Not applicable

Competing Interests

The authors declare they have no competing interests.

Authors Contributions

J.G. introduced the problem. X.S., F.W. initiated this project. X.S. designed and implemented the CloudMerge project. X.S. drafted the manuscript. X.S., J.P., F.W. and Z.Q. revised the manuscript.

Acknowledgements

We thank the three referees for their constructive critiques and detailed comments. We are grateful to Ms. Mary Taylor Mann and Ms. Alyssa Leann Duck for their editorial help during writing and revising of the manuscript.

References

1. Massie M, Nothaft F, Hartl C, Kozanitis C, Schumacher A, Joseph AD, et al. Adam: Genomics formats and processing patterns for cloud scale computing. University of California, Berkeley Technical Report, No UCB/EECS-2013. 2013;207.

2. Siretskiy A, Sundqvist T, Voznesenskiy M and Spjuth O. A quantitative assessment of the hadoop framework for analyzing massively parallel dna sequencing data. Gigascience. 2015;4 1:26.
3. Merelli I, Pérez-Sánchez H, Gesing S and D'Agostino D. Managing, analysing, and integrating big data in medical bioinformatics: open problems and future perspectives. BioMed research international. 2014;2014.
4. Reyes-Ortiz JL, Oneto L and Anguita D. Big data analytics in the cloud: Spark on hadoop vs mpi/openmp on beowulf. Procedia Computer Science. 2015;53:121-30.
5. Burren OS, Guo H and Wallace C. VSEAMS: a pipeline for variant set enrichment analysis using summary GWAS data identifies IKZF3, BATF and ESRRA as key transcription factors in type 1 diabetes. Bioinformatics. 2014;30 23:3342-8. doi:10.1093/bioinformatics/btu571.
6. Apache Hadoop. http://hadoop.apache.org/. Accessed 10 Oct 2017.
7. Dean J and Ghemawat S. Mapreduce: Simplified data processing on large clusters. Commun Acm. 2008;51 1:107-13. doi:Doi 10.1145/1327452.1327492.
8. Vora MN. Hadoop-HBase for large-scale data. In: *Computer science and network technology (ICCSNT), 2011 international conference on* 2011, pp.601-5. IEEE.
9. Zaharia M, Chowdhury M, Das T, Dave A, Ma J, McCauley M, et al. Resilient distributed datasets: A fault-tolerant abstraction for in-memory cluster computing. In: *Proceedings of the 9th USENIX conference on Networked Systems Design and Implementation* 2012, pp.2-. USENIX Association.
10. McKenna A, Hanna M, Banks E, Sivachenko A, Cibulskis K, Kernytsky A, et al. The Genome Analysis Toolkit: a MapReduce framework for analyzing next-generation DNA sequencing data. Genome research. 2010;20 9:1297-303.
11. Schatz MC. CloudBurst: highly sensitive read mapping with MapReduce. Bioinformatics. 2009;25 11:1363-9. doi:10.1093/bioinformatics/btp236.
12. Pireddu L, Leo S and Zanetti G. SEAL: a distributed short read mapping and duplicate removal tool. Bioinformatics. 2011;27 15:2159-60.
13. Niemenmaa M, Kallio A, Schumacher A, Klemelä P, Korpelainen E and Heljanko K. Hadoop-BAM: directly manipulating next generation sequencing data in the cloud. Bioinformatics. 2012;28 6:876-7.
14. Langmead B, Schatz MC, Lin J, Pop M and Salzberg SL. Searching for SNPs with cloud computing. Genome Biol. 2009;10 11:R134. doi:10.1186/gb-2009-10-11-r134.
15. Wang S, Mares MA and Guo YK. CGDM: collaborative genomic data model for molecular profiling

data using NoSQL. Bioinformatics. 2016;32 23:3654-60. doi:10.1093/bioinformatics/btw531.
16. Zhou W, Li R, Yuan S, Liu C, Yao S, Luo J, et al. MetaSpark: a spark-based distributed processing tool to recruit metagenomic reads to reference genomes. Bioinformatics. 2017;33 7:1090-2.
17. Niu B, Zhu Z, Fu L, Wu S and Li W. FR-HIT, a very fast program to recruit metagenomic reads to homologous reference genomes. Bioinformatics. 2011;27 12:1704-5.
18. AWS Genomics Guide. https://d0.awsstatic.com/Industries/HCLS/Resources/AWS_Genomics_WP.pdf. Accessed 10 Oct 2017.
19. Gruber K. Google for genomes. Nature Research, 2014.
20. O'Brien AR, Saunders NF, Guo Y, Buske FA, Scott RJ and Bauer DC. VariantSpark: population scale clustering of genotype information. BMC genomics. 2015;16 1:1052.
21. Wiewiórka MS, Messina A, Pacholewska A, Maffioletti S, Gawrysiak P and Okoniewski MJ. SparkSeq: fast, scalable and cloud-ready tool for the interactive genomic data analysis with nucleotide precision. Bioinformatics. 2014;30 18:2652-3.
22. Decap D, Reumers J, Herzeel C, Costanza P and Fostier J. Halvade: scalable sequence analysis with MapReduce. Bioinformatics. 2015;31 15:2482-8.
23. He M, Person TN, Hebbring SJ, Heinzen E, Ye Z, Schrodi SJ, et al. SeqHBase: a big data toolset for family based sequencing data analysis. Journal of medical genetics. 2015:jmedgenet-2014-102907.
24. Purcell S, Neale B, Todd-Brown K, Thomas L, Ferreira MA, Bender D, et al. PLINK: a tool set for whole-genome association and population-based linkage analyses. Am J Hum Genet. 2007;81 3:559-75. doi:10.1086/519795.
25. Mohammed EA, Far BH and Naugler C. Applications of the MapReduce programming framework to clinical big data analysis: current landscape and future trends. BioData Min. 2014;7:22. doi:10.1186/1756-0381-7-22.
26. Huang H, Tata S and Prill RJ. BlueSNP: R package for highly scalable genome-wide association studies using Hadoop clusters. Bioinformatics. 2013;29 1:135-6. doi:10.1093/bioinformatics/bts647.
27. White T. Hadoop: The definitive guide. " O'Reilly Media, Inc."; 2012.
28. Silberschatz A, Korth HF and Sudarshan S. DatabaseSystem Concepts. 2010.
29. Danecek P, Auton A, Abecasis G, Albers CA, Banks E, DePristo MA, et al. The variant call format and

VCFtools. Bioinformatics. 2011;27 15:2156-8. doi:10.1093/bioinformatics/btr330.

30. Multiway-Merge Algorithm. https://en.wikipedia.org/wiki/K-Way_Merge_Algorithms. Accessed 10 Oct 2017.

31. Chang F, Dean J, Ghemawat S, Hsieh WC, Wallach DA, Burrows M, et al. Bigtable: A distributed storage system for structured data. Acm T Comput Syst. 2008;26 2 doi:Artn 4
10.1145/1365815.1365816.

32. Genomes Project C, Abecasis GR, Altshuler D, Auton A, Brooks LD, Durbin RM, et al. A map of human genome variation from population-scale sequencing. Nature. 2010;467 7319:1061-73. doi:10.1038/nature09534.

33. Mathias RA, Taub MA, Gignoux CR, Fu W, Musharoff S, O'Connor TD, et al. A continuum of admixture in the Western Hemisphere revealed by the African Diaspora genome. Nat Commun. 2016;7:12522. doi:10.1038/ncomms12522.

34. Kwon Y, Balazinska M, Howe B and Rolia J. A study of skew in mapreduce applications. Open Cirrus Summit. 2011;11.

35. O'Neil P, Cheng E, Gawlick D and O'Neil E. The log-structured merge-tree (LSM-tree). Acta Informatica. 1996;33 4:351-85.

36. Li H. Tabix: fast retrieval of sequence features from generic TAB-delimited files. Bioinformatics. 2011;27 5:718-9.

37. Amdahl GM. Validity of the single processor approach to achieving large scale computing capabilities. In: *Proceedings of the April 18-20, 1967, spring joint computer conference* 1967, pp.483-5. ACM.

38. Gustafson JL. Reevaluating Amdahl's law. Commun Acm. 1988;31 5:532-3.

39. Sedgewick R and Flajolet P. An introduction to the analysis of algorithms. Addison-Wesley; 2013.

40. Özsu MT and Valduriez P. Principles of distributed database systems. Springer Science & Business Media; 2011.

41. Miner D and Shook A. MapReduce Design Patterns: Building Effective Algorithms and Analytics for Hadoop and Other Systems. " O'Reilly Media, Inc."; 2012.

42. Chen L, Wang C, Qin ZS and Wu H. A novel statistical method for quantitative comparison of multiple ChIP-seq datasets. Bioinformatics. 2015;31 12:1889-96.

Figure legends

Figure 1. Merging multiple VCF files into a single TPED file. Left tables represent input VCF files. Table to the right represents the merged TPED file. Records are filtered out if their Filter value does not equal to "PASS" (Pos 10147). Individual genotypes from multiple VCF files with the same genomic location are aggregated together in one row. The resulting TPED file thus has an inclusive set of sorted genomic locations of all variants found in the input VCF files.

Figure 2. The workflow chart of the MapReduce schema. The workflow is divided into two phases: In the first phase, variants are filtered, grouped by chromosomes into bins, and mapped into key-value records. Two sampling steps are implemented to generate partition lists of all chromosomes. In the second phase, parallel jobs of specified chromosomes are launched. Within each job, records from corresponding bins are loaded, partitioned, sorted and merged by genomic locations before being saved into a TPED file.

Figure 3. The workflow chart of the HBase schema. The workflow is divided into three phases. The first is a sampling, filtering and mapping phase. A MapReduce job samples out variants

whose genomic positions are used as region boundaries when creating the HBase table. Only qualified records are mapped as key-values and saved as Hadoop sequence files. The second is the HBase bulk loading phase in which a MapReduce job loads and writes records generated from the previous phase, aggregating them into corresponding regional HFiles in the form of HBase's row key and column families. Finished HFiles are moved into HBase data storage folders on region servers. In the third phase, parallel scans were launched over regions of the whole table to retrieve desired records which are subsequently merged and exported to the TPED file.

Figure 4. The workflow chart of the Spark schema. The workflow is divided into three stages. In the first stage, VCF records are loaded, filtered, and mapped to PairRDDs with keys of genomic position and values of genotype. The sort-by-key shuffling spans across the first two stages, sorting and grouping together records by keys. Then grouped records with the same key are locally merged into one record in TPED format. Finally, merged records are exported to the TPED file.

Figure 5. The execution plan of the HPC-based implementation. The execution plan resembles

a branched-tree. In the first round, each process is assigned an approximately equal number of files to merge locally. In the second round, even-numbered process retrieves the merged file of its right adjacent process to merge with its local merged file. In the third round, processes whose ID can be fully divided by four retrieve the merged file of its right adjacent process in the second round and do the merging. This process continues recursively until all files are merged into a single TPED file (round four).

Figure 6. The scalability of Apache cluster-based schemas on input data size. A. MapReduce schema. B. HBase schema. C. Spark schema. As the number of input files increases from 10 to 186, the time costs of all three schemas with 12, 24 or 72 cores increase in a slower pace than that of the input data size, especially when the number of cores is relatively large. The HBase schema with 12 cores has the largest increase (from 375 to 5,479 seconds, ~14.6 fold).

Figure 7. Comparing the strong scalability between traditional parallel/distributed methods and Apache cluster-based schemas. We fix the number of files at 93 and increase the number of nodes/cores. The baseline for the parallel multiway-merge is one single core, while for the others

is one single node (4 cores). All methods/schemas show a degraded efficiency as computing resources increase 16 fold from the baseline. Specifically, the efficiency of MapReduce-, HBase-, Spark-based schemas drops to 0.83, 0.63 and 0.61 respectively, while the efficiency of parallel multiway-merge and HPC-based implementations drops to 0.06 and 0.53 respectively.

Figure 8. Comparing the weak scalability between traditional parallel/distributed methods and Apache cluster-based schemas. We simultaneously increase the number of cores and input data sizes while fixing the ratio of file/core (parallel multiway-merge) or file/node (all others) at ten. The baseline is the same as in the test of strong scalability. All but the MapReduce-based schema have degraded efficiency, among which the HPC-based implementation has the steepest degradation. Specifically, when computing resource increases 16 fold from the baseline, the efficiency of MapReduce-, HBase- and Spark-based schemas changes to 3.1, 0.87 and 0.75 respectively, and for parallel multiway-merge and HPC-based implementations, the efficiency reduces to 0.42 and 0.35 respectively.

Figure 9. The performance anatomy of cluster-based schemas on increasing input data size.

The number of cores in these experiments is fixed at 48. Time costs of all phases of the three schemas have a linear or sub-linear correlation with the input data size. **a)** MapReduce schema: The two MapReduce phases have a comparable time cost, increasing 6.3- and 3.1-fold respectively as the number of input files increases from 10 to 186. **b)** HBase schema: The time spent in each phase increases 4.2-, 5.6- and 5.0-fold respectively as the number of input files increases from 10 to 186. The bulk loading and exporting phases together take up more than 80% of total time expense. **c)** Spark schema: The time cost increases 5.8-, 6.0- and 6.0-fold respectively for the three stages as the number of input files increases from 10 to 186 files. Like the HBase schema, the first two stages of the Spark schema together account for more than 80% of the total time cost.

Figure 10. Execution speed comparison among Apache cluster-based schemas and traditional methods. Firstly, we compare of the speeds of the three Apache schemas with that of three traditional methods which are single-process multiway-merge, parallel multiway-merge and HPC-based implementations. As the number of input files increases from 10 to 186, the speeds of Apache cluster-based schemas improve much more significantly than traditional methods. The

numbers in the figures indicate the ratio of the time cost of each traditional method to that of the fastest Apache cluster-based schema. Secondly, we compare the processing speed among the three Apache cluster-based schemas which are comparable to each other regardless of the input data size. The MapReduce schema performs the best in merging 10 and 186 files; The HBase schema performs the best in merging 20, 40 and 60 files; The Spark schema performs the best in merging 93 files.

Figure S1. Pseudocodes of the MapReduce schema.

Figure S2. Pseudocodes of the HBase schema.

Figure S3. Pseudocodes of the Spark schema.

Tables

Table 1. Performance comparisons between VCTools and Apache cluster-based schemas

	VCFTools	MapReduce	HBase	Spark

**Time cost (seconds)**	30,189	484	577	596
**Fold (faster)**	-	62.4	52.3	50.7

Table 2. Pros and Cons of MapReduce, HBase and Spark schemas

Schemas	***Pros***	***Cons***
**MapReduce**	• Good for large input data size and sufficient computing resources.<br>• Simple architecture and least overheads given sufficient computing resources.<br>• Best parallelism<br>• Good for one-time merging.<br>• Performance is stable.	• Merging is not incremental.<br>• Much overheads when computing resources are limited
**HBase**	• Good for intermediate input data size (>=20 and <=100 VCF files).<br>• Supports incremental merging.<br>• Supports On-Line Analytical Processing (OLAP).<br>• Best storage efficiency.	• Users must determine region number in advance.<br>• Has most local I/O.<br>• Complex performance tuning.

**Spark**	• Good for large input data size (>100 VCF files) and relative limited computing resources.<br>• Keeps intermediate results in memory and least local I/O.<br>• Good for subsequent statistical analysis on merged results.	• Possibly weakened data locality during loading.<br>• Slight unstable performance when computing resources exceeds needs of input data size.<br>• Actual execution plan is not transparent.<br>• Complex performance tuning.

791

Figure1

Click here to download Figure Figure1.pdf

VCF File 1

Chr	Pos	Ref	Alt	Filter	...	Genotype
1	10147	A	T	q20	...	1/0:43
1	10240	T	G	PASS	...	1/0:5
...	...	...	...	...	...	...
Y	11590	G	C	PASS	...	0/0:10

VCF File 2

Chr	Pos	Ref	Alt	Filter	...	Genotype
1	10186	G	A	PASS	...	1/0:9
1	10240	T	G	PASS	...	1/1:11
...	...	...	...	...	...	...
Y	11872	G	T	PASS	...	0/1:10

Genotypes

Chr	Rs	Distance	Pos	Ind_1	Ind_2
1	.	0	10186	G G	G A
1	.	0	10240	T G	G G
...	...	...	...	...	...
Y	.	0	11590	G G	G G
Y	.	0	11872	G G	G T

Merged TPED file

Figure2

Click here to download Figure Figure2.pdf

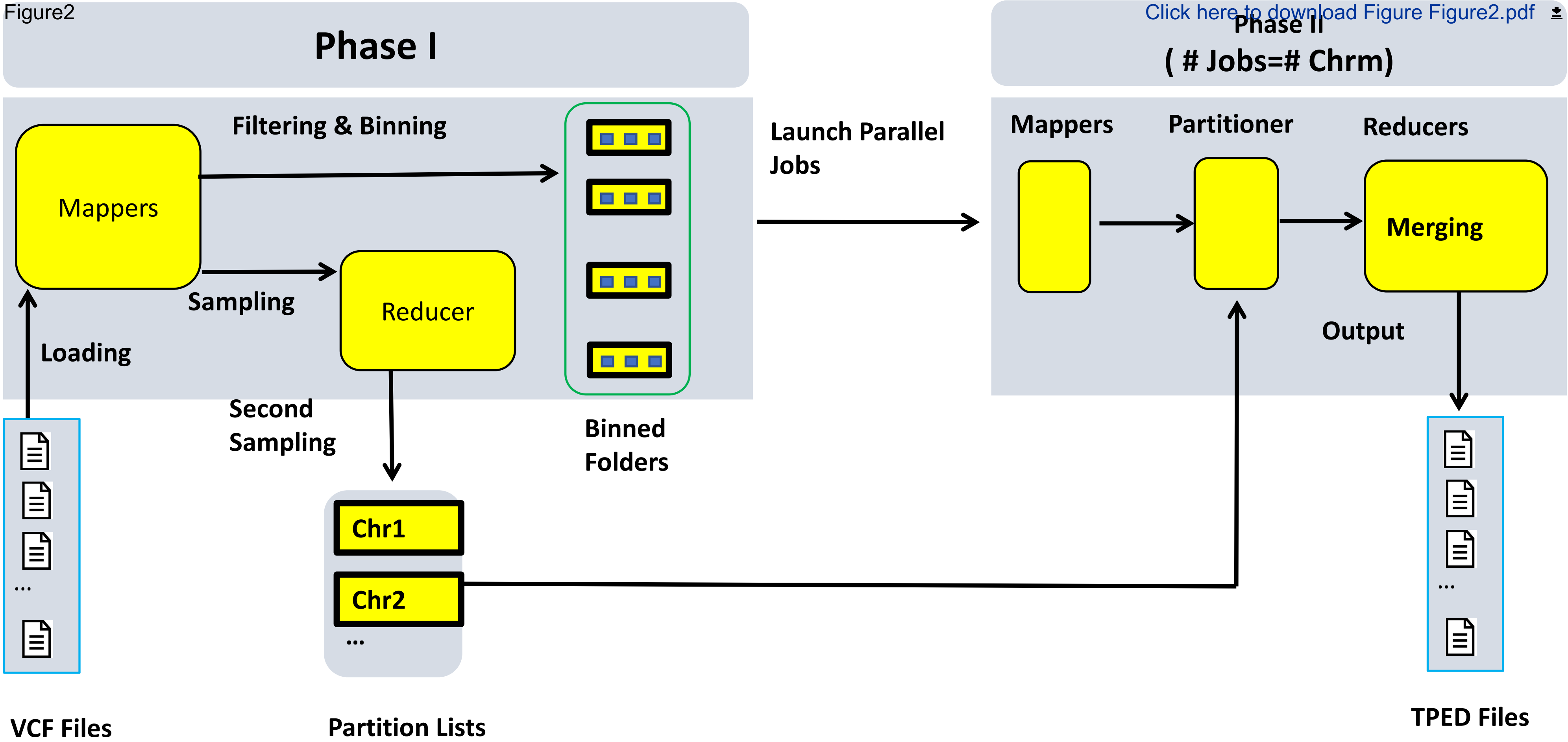

Click here to download Figure Figure3.pdf

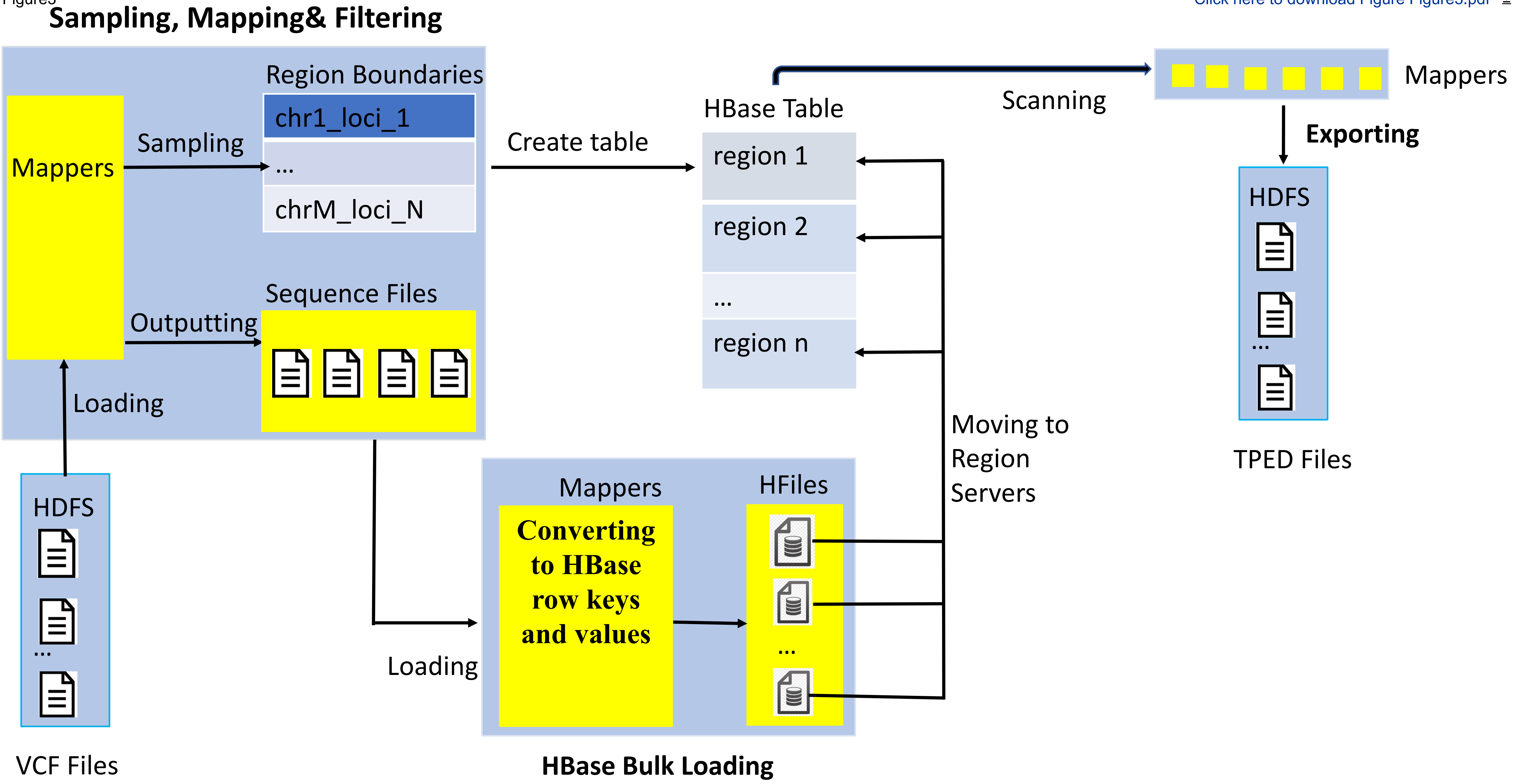

Figure4

Click here to download Figure Figure4.pdf

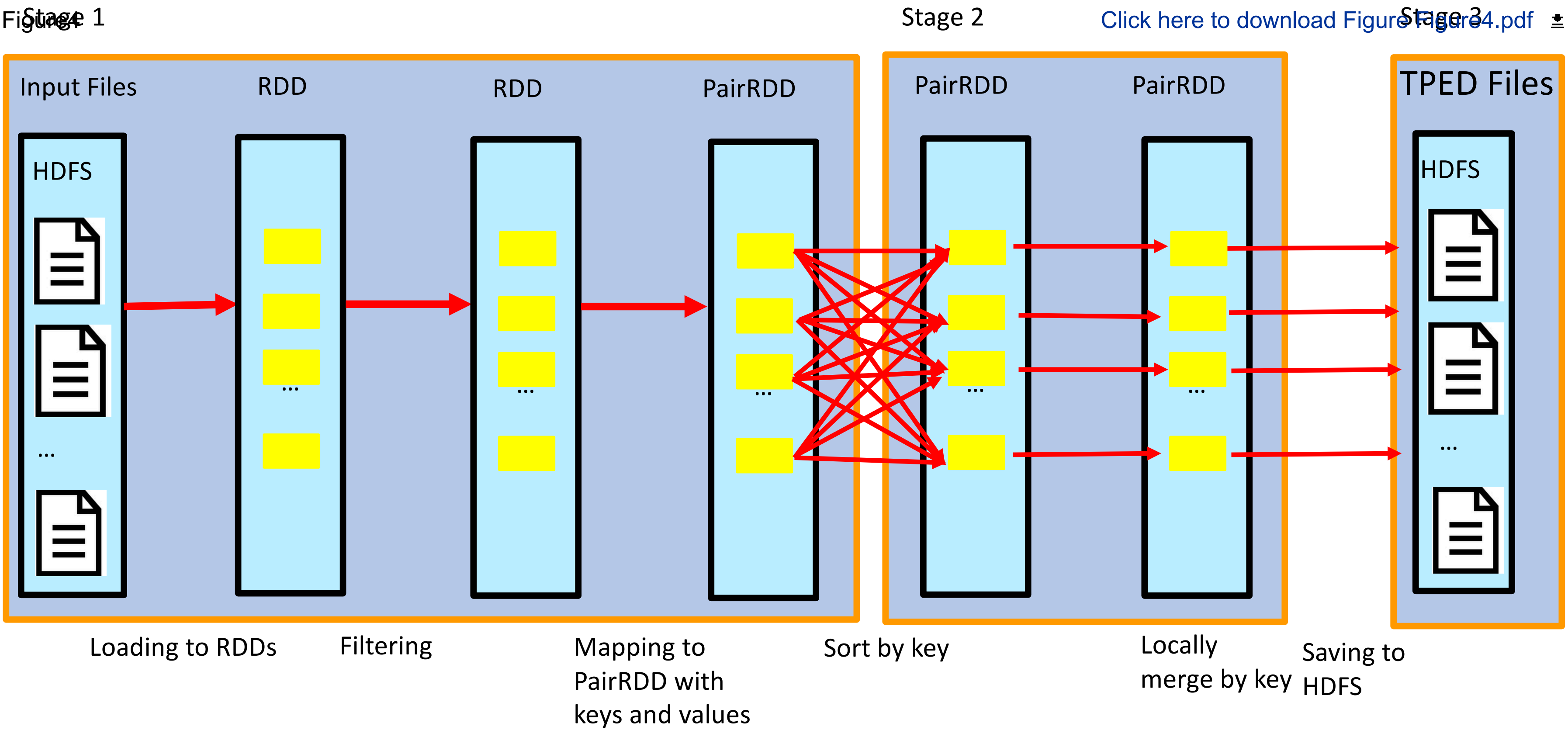

 Click here to download Figure Figure5.pdf

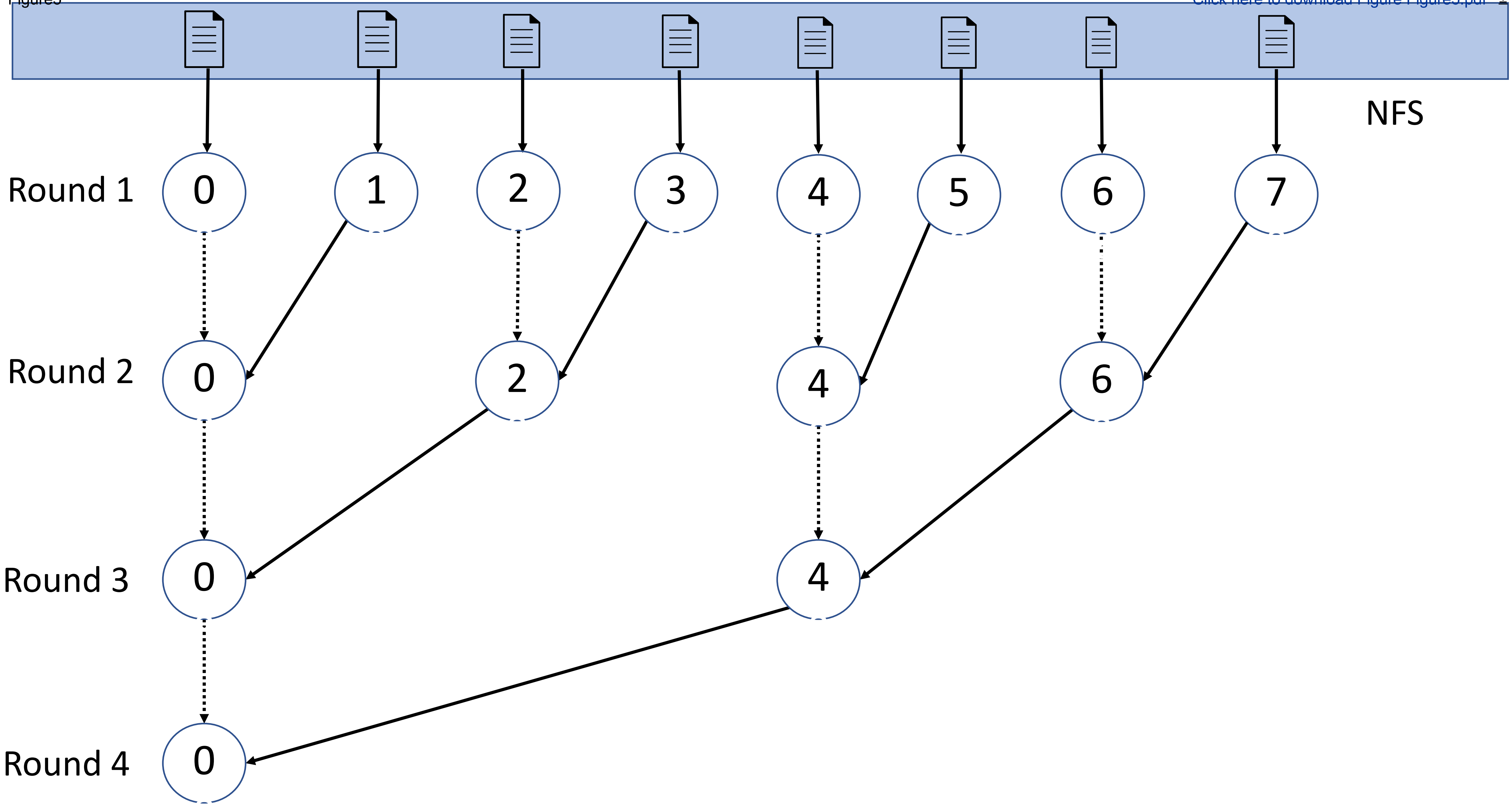

Figure6

Click here to download Figure Figure6.pdf

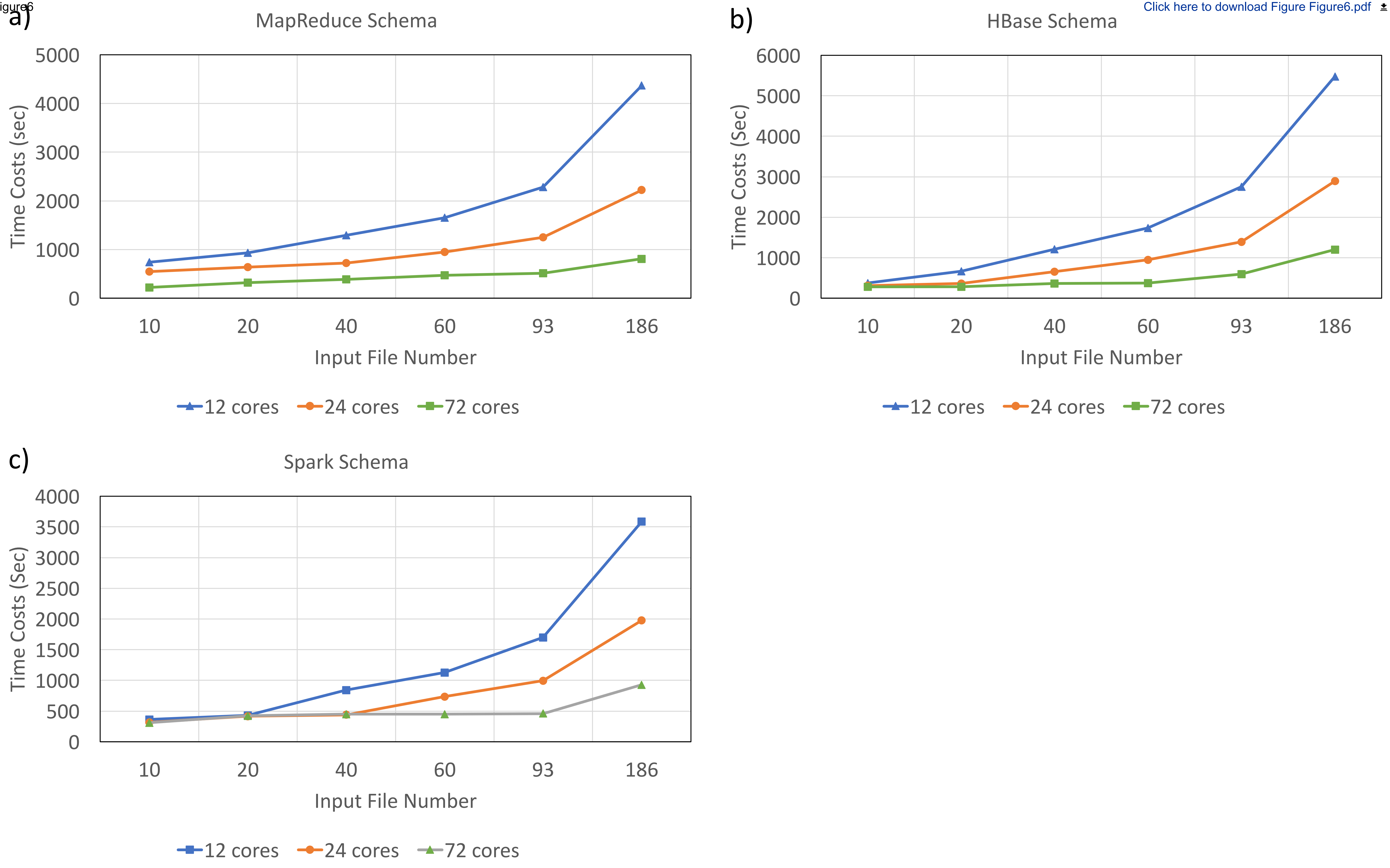

Figure7

Click here to download Figure Figure7.pdf

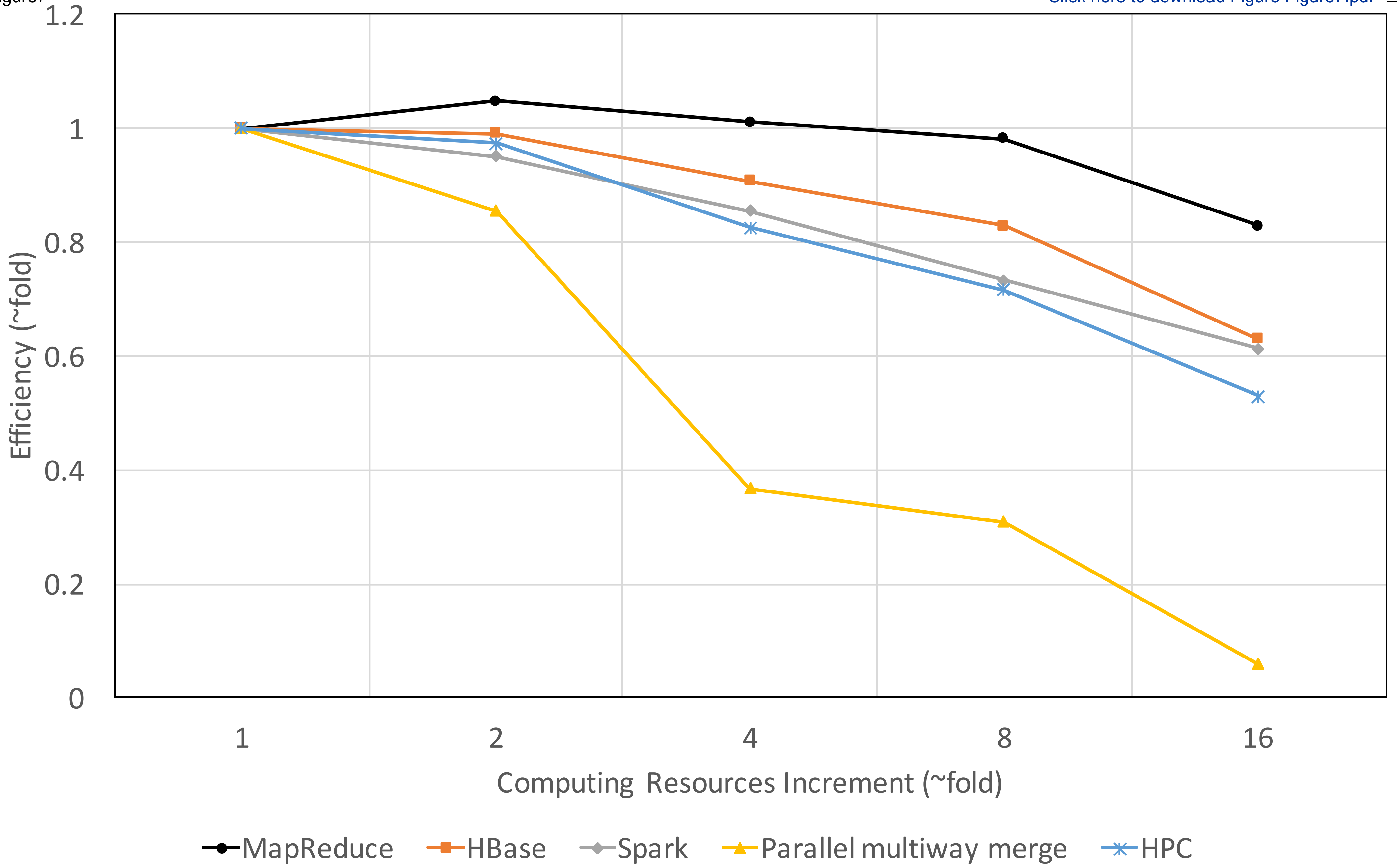

Figure8

Click here to download Figure Figure8.pdf

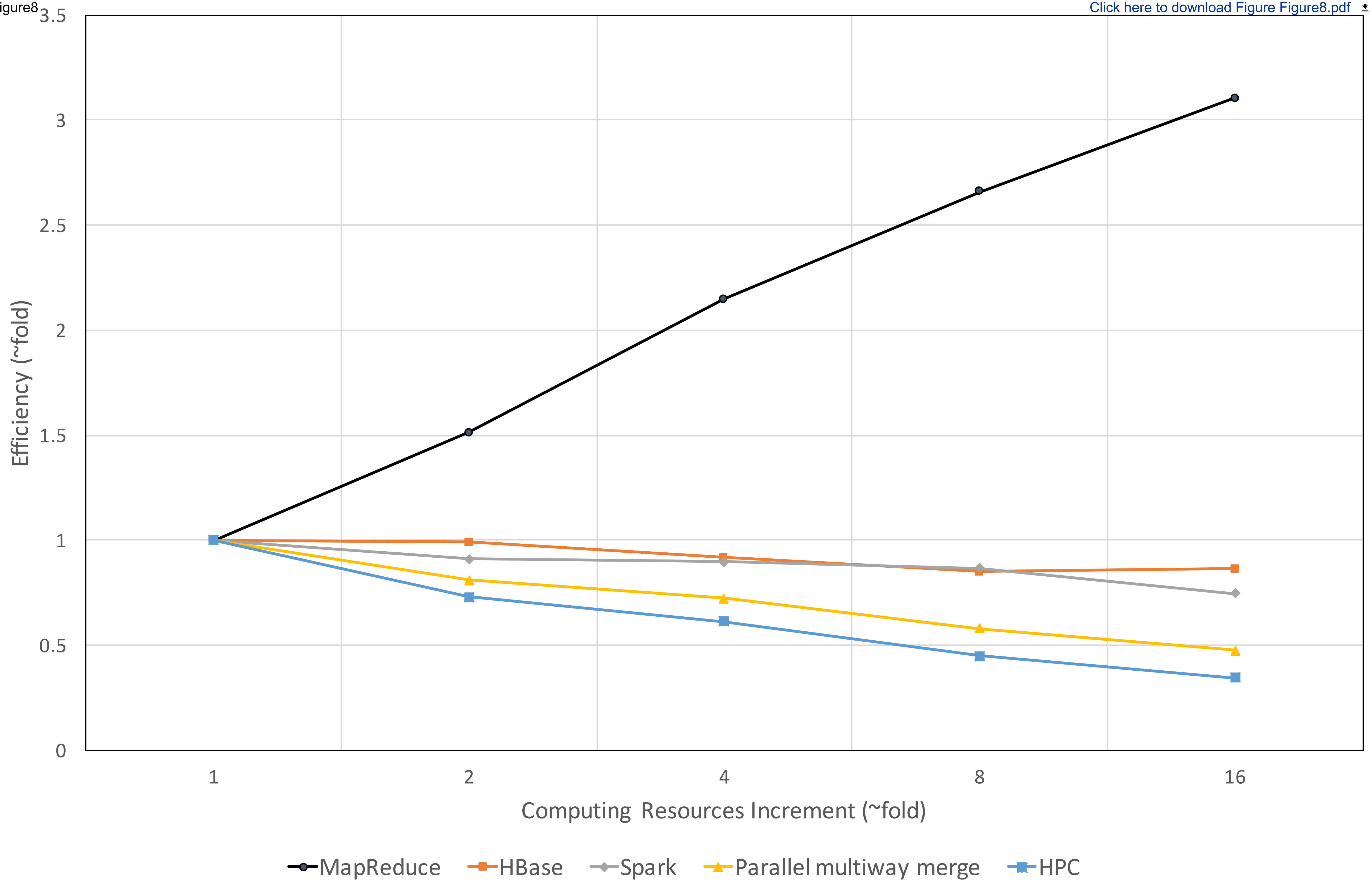

Figure9

Click here to download Figure Figure9.pdf

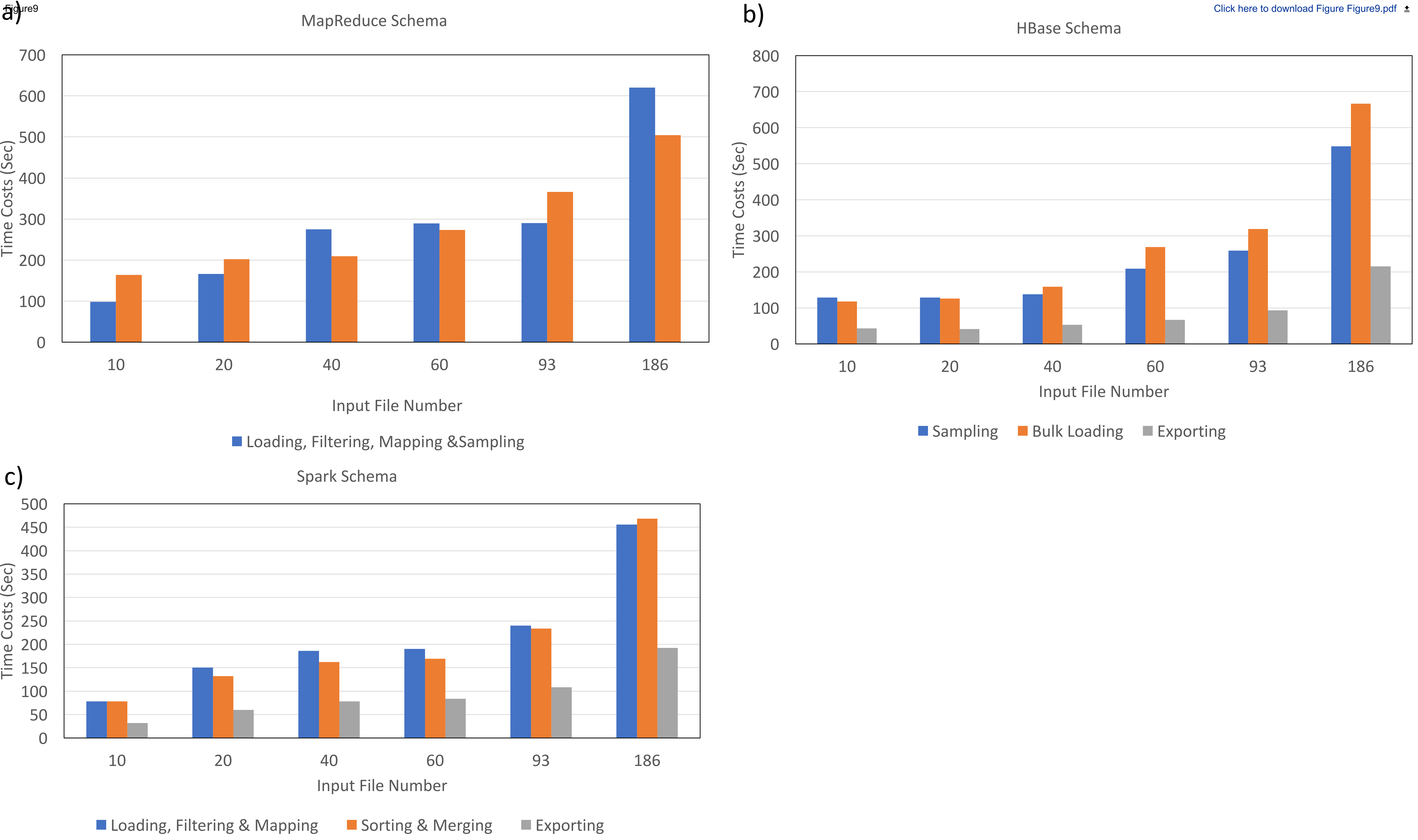

Figure10

Click here to download Figure Figure10.pdf

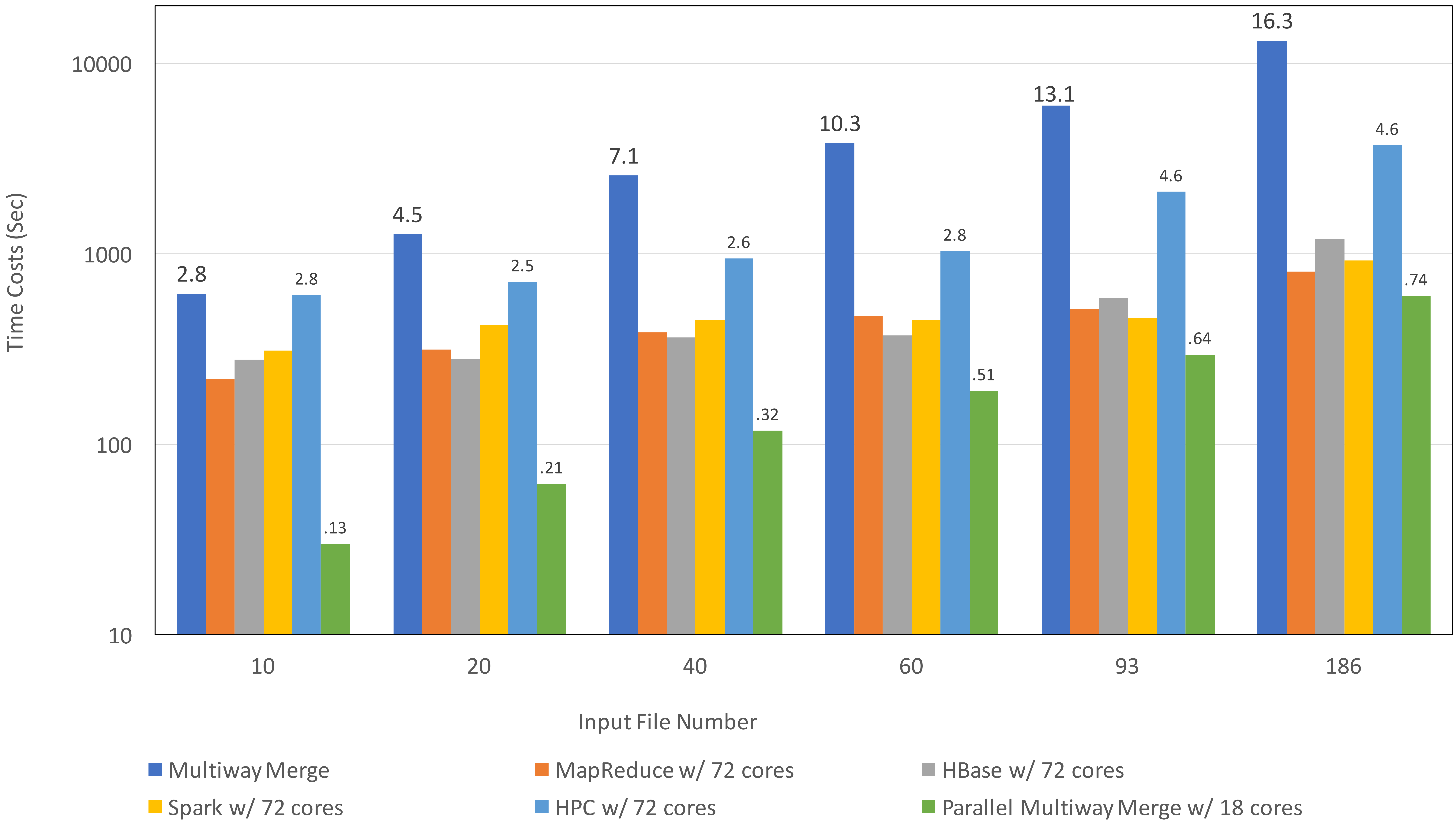

Supplementary Figure S1

Click here to access/download
Supplementary Material
FigureS1.pdf

Supplementary Figure S2

Click here to access/download
Supplementary Material
FigureS2.pdf

Supplementary Figure S3

Click here to access/download
Supplementary Material
FigureS3.pdf

Manuscript with highlighted changes

Click here to access/download
Supplementary Material
reviewedmanuscriptmarked.docx

Cover and response letter

Click here to download Personal Cover Cover and response letter.submission.docx

EMORY
ROLLINS SCHOOL OF PUBLIC HEALTH
Department of Biostatistics and Bioinformatics

February 6, 2018

Laurie Goodman, PhD
Editor-in-Chief
GigaScience

RE: manuscript number GIGA-D-17-00267

Dear Dr. Goodman:

On behalf of my colleague, Xiaobo Sun, Jingjing Gao and Peng Jin, we pleased to submit the revised version of our manuscript titled:

Optimized Distributed Systems Achieve Significant Performance Improvement on Sorted Merging of Massive VCF Files

for publication consideration in GigaScience as a Technical Note. The original manuscript was submitted on October 12, 2017. We are grateful to you for giving us the opportunity to revise and resubmit.

In the revision, we have made significant changes to the main manuscript and the supplementary material based on the reviewers' comments and suggestion. In addition to the revised manuscript, we also enclose a detailed point-by-point response letter to address the reviewer' comments, and a supplementary file with all the changes made highlighted in red. Please do not hesitate to contact us if you have any further question.

Thank you very much for your kind editorial assistance.

Sincerely,

Zhaohui (Steve) Qin, Ph.D.
Associate Professor
Department of Biostatistics and Bioinformatics
Emory University
Atlanta, GA 30322

Fusheng Wang, Ph.D.
Assistant Professor
Department of Biomedical Informatics
Department of Computer Science
Stony Brook University
2313D Computer Sciences
Stony Brook, NY 11794-8330

We thank the reviewers for their thorough reviews, thoughtful comments and constructive suggestions. We have taken the reviewers' comments seriously and revised the manuscript and Supplementary Material accordingly. We believe the revised version is able to better communicate the contribution of our manuscript. To be specific, major modifications in this revision are summarized below:

- Implemented two parallel benchmark methods which are parallel multiway-merge running on a single machine and HPC-based sorted merging method. Included these two methods in performance comparison.
- Added experiments of strong and weak scalabilities on both Apache cluster-based schemas and benchmarks.
- Increased dataset size from 93 input VCF files to 186, and updated the results.
- Added more references about big data and applications of Apache systems in bioinformatics.
- Removed overstatements, corrected grammar mistakes.
- Changed the title to "… Sorting and Merging Massive VCF files" based on reviewers' opinions to narrow down and more accurately reflect the types of data that our methods can be applied to.

Below we present itemized responses to all the comments, organized by reviewers. The reviewers' comments are in **bold**. Our responses are in dark blue color.

Reviewer #1:

This paper is a cross between a case study and an application note. As a case study it considers the task of improving the performance of sorted merging of variant call data using parallel computation. As an application note it provides software implementations of tools which can be downloaded and used by bioinformatics practitioners. Although the case study considers sorting variant call data (VCF files) the techniques developed can easily be extended to other coordinate oriented data sets.

The paper describes in moderate detail the implementation of three different parallelisation techniques based on the Apache distributed platforms: Hadoop (MapReduce), HBase, Spark.

It compares the performance of these implementations for strong and weak scaling (though the authors do not use this terminology) against each other, and also against non-distributed parallel versions using the widely used package VCFTools, and also a custom built multiway-merge implementation. The authors also consider the performance characteristics of different phases of their parallel implementations to see whether any of them might suffer bottlenecks when scaling to larger data sets.

The performance outcomes are fairly predictably in favour of the distributed parallel systems. However, the authors have a tendency to overstate the significance of the outcomes, saying that "Traditional single machine based methods are no longer feasible to process big data due to the prohibitive computation time and I/O bottleneck".

I do not believe that the results of this paper show that single node merging is infeasible, rather they show that distributed parallel techniques can scale to larger data sets. This is something we have known for a long time. The authors also say that "The newly distributed systems have the potential to offer a much needed boost in performance", however, distributed systems are now decades-old ideas. Also, the paper does not really attempt to investigate similar strong and weak scaling for the single node implementations.

We thank the reviewer's comments. We apologize for making the overstatements. We took the reviewer's advice and have rephrased the two sentences as following.

On line 21:
"Traditional single machine based methods are no longer feasible to process big data due to the prohibitive computation time and I/O bottleneck"
Changed to:
"Traditional single machine based methods become increasingly inefficient when processing hundreds or even thousands of VCF files due to the excessive computation time and I/O bottleneck."

On line 23:
"The newly distributed systems have the potential to offer a much needed boost in performance"
Changed to:

“The distributed systems and the more recent cloud-based systems offer an attractive solution.”

Following the reviewer’s advice, we investigated strong and weak scalabilities for single node implementation. We implemented a parallel multiway-merger running on a single node to test its performance in terms of strong and weak scaling.

For scaling test, there are three ways to implement the merger on a single node:

1. Simply increasing the number of CPUs of the single node. However, the implementation is based on multiway-merge algorithm, which can only allow one writer (single process or thread) to retrieve records from the underlying priority queue data structure. As a result, when using just single multiway-merger, adding more CPUs won’t help improving the performance. However, there are two non-trivial ways for scaling which are illustrated below.

2. We can have multiple multiway-mergers run at the same time where each merger handles the merging of a subset of all files (***task-parallelism***). Then in the next round, merged files resulted from the previous round are gathered and merged again. This process continues until all files are merged into one file, which will take approximately log(n) rounds where n is the number of input files. This method is essentially our MPI-based HPC implementation running on a single node.

3. We can have multiple multiway-mergers run at the same time where each merger handles a non-overlapping genomic region of the same length from all input files (***data-parallelism***). Although this strategy sounds promising, it is not quite practical unless the data distribution across all files are uniform or we know the exact data distribution in advance. As a result, it is very difficult to get a balanced workload for each merger unless we sample through all the files to get the data distribution profile. In that case, if we are using single thread/process sampling, this will be slower than the single multiway merge. If we are using multi-thread/process sampling to do this, it essentially becomes the same as our Hadoop implementation running on a single machine but without task coordination offered by YARN. To make a compromise, we can assume the data distribution is uniform and try to assign each merger a genomic region of the same length. Additionally, when reading the input files, we do not want each merger to seek the beginning of its genomic region from the very beginning of the file. Instead we use TabixReader which takes advantage of the Tabix index built on the VCF file to seek directly to the right offset. In addition to the potential workload unbalance problem, this method also has three limitations: 1. Additional time costs are incurred for building the Tabix indices for each file. 2. Tabix can only be used for VCFs, so it cannot be generalized to other data formats. 3. Each process needs to maintain a whole set of file descriptors, Tabix indices, current data blocks of all files in memory, which could cause memory leak or extremely slow garbage collection (GC) time when input data size is large (in our Java implementation, 16 parallel mergers with 93 files consume over 100GB memory. This memory burden

may be slightly lower in C/C++ implementation). The following table summarizes the results from weak and strong scaling test running on a r4.8xlarge EC2 instance.

Strong scalability test:
Number of file: 93

# of cores	1	2	4	8	16
Time cost (seconds)	2,410	1,410	959	388	414

From strong scaling test, we can see that as the number of cores increases, the efficiency reduces significantly, indicating poor strong scalability. This is because the more processes, the more imbalanced the workload among processes. Additionally, the saturation of I/O to and from disk as well as higher memory management costs (GC, swap and so on) also contribute to its inefficiency. Results are displayed in Figure 7 in the manuscript.

Weak scalability test:

# of files	10	20	40	80	160
# of cores	1	2	4	8	16
Time cost (seconds)	242	299	334	417	508

From weak scaling test, we can see that although we fix the data size per process, because of the same reasons as in strong scalability test, the efficiency still decrease significantly. . Results are displayed in Figure 8 in the manuscript.

In conclusion, when input data size is relatively small, the parallel multiway-merge has significant performance advantage over cloud-based method because it avoids the data network transmission latency. However, when input data size is very large which is often the case in real applications, the problem of workload imbalance across all processes, I/O saturation and memory burden worsens significantly. As a result, this method no longer scale well.

In the manuscript, we added “*Parallel Multiway-Merge and MPI-based High Performance Computing Implementations*” (line 298) and “*Strong and Weak Scalabilities*” (line 348) sections under the *Method* to describe how they are implemented and how the tests of strong and weak scalabilities are conducted. We also added a “*Comparing Strong and Weak Scalabilities between Apache Cluster-based Schemas and Traditional Parallel Methods*” (line 396) section under the *Results* to show and compare the strong and weak scalabilities of Apache cluster-based schemas with parallel multiway-merge and HPC-based implementations. Finally, we compared the execution speed of both parallel multiway-merge and HPC-based implementations with the Apache cluster-based schemas in the “*Comparing Execution Speed between Apache Cluster-based Schemas and Traditional Methods*” (line 453) section in the Results.

I believe that the authors have also overstated the scalability of the distributed implementations, saying they have "nice" scalability (nice is a poor choice of adjective in a scientific paper), however, the results shown in figures 5 and 6 show distinct degradation in both strong and weak scaling at higher core counts/input sizes.

> We agree with the reviewer and have rephrased the statement to the following (line 380): “As Figure 6 shows, for all three schemas, given a fixed number of cores, the execution time increases in a slower pace than that of the input data size.”

One thing missing from the paper is a comparison to a distributed parallel version not based on Apache platforms. That is to say, could we achieve similar performance results on a traditional HPC cluster with a shared filesystem to the demonstrated cloud-based solutions? This question is important to consider, because many bioinformatics organisations have access to HPC systems, and heterogenous cloud-HPC platforms are still difficult to manage. It is not clear whether the effort required to implement systems within the Apache frameworks is justified, compared to a comparatively simpler approach on a traditional HPC system.

> We thank the reviewer for raising this important question. There are two HPC-based solutions for this problem: data parallelism and task parallelism. However, data parallelism is difficult to achieve because we do not know the data distribution. Randomly splitting and assigning data to HPC nodes causes workload imbalance and jeopardies the overall performance. We could do parallel sampling to acquire the distribution profile, but this will make its workflow the same as that of the MapReduce-based schema with the same operations and the same order: sampling in parallel, dividing the dataset into splits of equal sizes, and assigning the splits to processes to perform the merging. But this implementation is without data locality offered by HDFS and task coordination offered by YARN and thus has a performance no better than the MapReduce-based schema. Consequently, we only implement and test the task parallelism using openMPI as following: firstly, we scatter an approximate number of input files to each HPC process across the whole cluster. Each process fetches the assigned files from the NFS system and do the multiway-merging locally. Secondly, in the following rounds, we adopt a tree-structured merging strategy. To be specific, in the second round, processes whose id number is even (process id starts from 0) retrieve merged files from its adjacent process to the right and do the local multiway-merging. In the third round, processes whose id can be fully divided by 4 retrieve merged results of its adjacent process to the right in the second round. For example, process 4 will receive merged results from process 6. This process continues until all the files are merged into a single file. The total number of rounds is $\log(n)$.

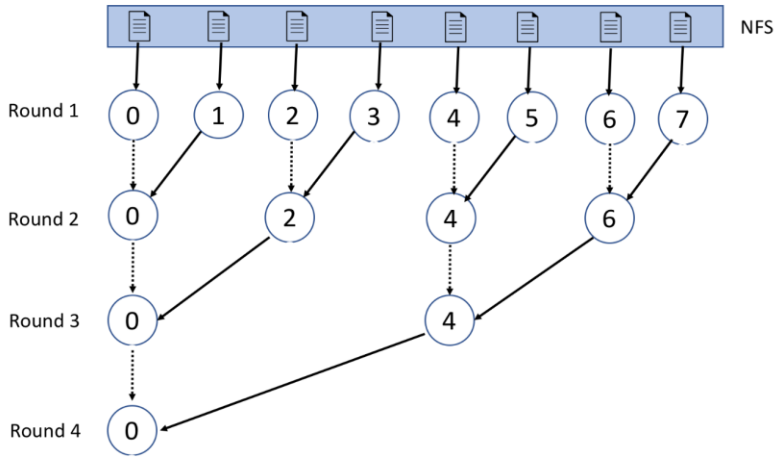

Strong scalability test:
Number of file: 93

# of nodes (# of processes)	1 (4)	2 (8)	4 (16)	8 (32)	16 (64)
Time cost (seconds)	17745	9105	5377	3098	2093

In the strong scaling test, the efficiency drops significantly as we add more nodes and processes. This is because although adding nodes lead to a better parallelism, the workload imbalance (because it is almost impossible to assign each node exactly the same number of files) is also increased among nodes in every round. Results are displayed in Figure 7 in the manuscript.

Weak scalability test:

# of files	10	20	40	80	160
# of nodes(# of processes)	1 (4)	2 (8)	4 (16)	8 (32)	16(64)
Time cost (seconds)	1311	1799	2142	2919	3777

In the weak scaling test, we fixed the input data size per node. The efficiency drops even more because of increased number of merging rounds. Results are displayed in Figure 8 in the manuscript.

In conclusion, we showed that sorted merging by traditional HPC (task parallelism) shows inferior scalability and are much slower than the methods running on Apache cluster-based framework.

We have added the description of HPC-based implementation and its tests of strong and weak scalabilities in the “*Parallel Multiway-Merge and MPI-based High Performance Computing Implementations*” (line 298) section and “Strong and Weak Scalabilities” (line 348) section.

Perhaps the main contribution of this paper is not the resulting software tools, but the insights provided into parallelisation techniques on the Apache cloud-based platforms. Here, the paper exhibits more value as a case study than as an application note. The methods section makes up a considerable portion of the paper, and attempts to explain the main ideas underpinning each implementation, and also contains some useful observations about potential pitfalls in each approach. Generally speaking I find it difficult to closely follow algorithms when they are described as prose, and this paper suffers from a lack of clarity in the explanations of each implementation. Figures 2-4 do help with the presentation, though I wonder whether a pseudo-code style presentation might also be helpful?

We thank the reviewer for the suggestion. We now have included pseudo-code presentation in Figure S1-S3

Overall I think the paper will be of most interest to bioinformatics software implementors who are investigating the parallelisation of tools on cloud-based platforms. It is probably of less interest to bioinformatics practitioners, who are interesting in putting the tools into use.

We agree with the reviewer’s comment.

Below are some remarks about the presentation which might improve future revisions:

We really appreciate the reviewer’s careful proofreading and specific corrections!

- (page 2) "achieve maximum performance" -> "achieve high performance" (it is not clear that "maximum" performance is well defined)

Done. See line 26.

- there are numerous instance where the definite article "the" is incorrectly used. For example:
(page 3) "of _the_ powerful computing resources" (should be "of powerful computing resources")
(page 5) "applications of Apache big data platform" (should be "of _the_ Apache big data platform")
(page 21) "More specifically, MapReduce-based schema" (should be "More specifically, _the_ MapReduce-based schema")
I have not listed all examples, and recommend a thorough proof read before final submission.

We have conducted a thorough proof read and corrected similar problems.

- (page 4) "takes advantage of" -> "take advantage of" (because you are talking about two different tools)

Done. See line 55.

- (page 4) "researchers have recently started to embrace distributed systems" -> I don't think this is really true. Distributed systems have been in use in bioinformatics for quite some time now. The trend towards cloud-based and hadoop-styled computations is somewhat more recent, but still not new.

We agree with reviewer's opinion.
On line 61:
We change it to: "In bioinformatics, researchers have already started to embrace Apache distributed systems to manage and process large amount of high throughput omics data."

- (page 5) "plenty of" -> "many"

Done. See line 76.

- (page 5) maybe replace "single-threaded" with "sequential"

Done. See line 226.

- (page 6) it is not clear what "working schemas" means here. Also it might be best to describe what you mean by "schemas" in general, since that is an important concept in the paper.

We are sorry for the confusion and in the abstract part (line 24) add: "However, carefully designed and optimized working flow patterns and execution plans (schemas) are required to take full advantage of the increased computing power while overcoming bottlenecks to achieve high performance."

- (page 7) "boosts" -> "improves"

Done. See line 114.

- there are numerous instances where pluralisation is done incorrectly. For example:
(page 8) "bottleneck and hotspot can happen" (should be "bottlenecks and hotspots can happen"), and "unbalanced workload" (should be "unbalanced workloads", or "an unbalanced workload")
(page 10) "locations that appears" (should be "locations that appear")
(page 11) "of interests" (should be "of interest")
(page 14) "finishing writings" (should be "finishing writing")
again, a thorough proof reading of the paper before submission should address these issues
- (page 8) "hit" -> "access" (likewise on page 14)
- (page 9) "In a typical WGS" -> "In a typical WGS experiment"

- (page 9) "one of the individual's" -> "one of an individual's"

We have corrected all of these and other places we found.

- (page 10) mentions an "unqualified filter": this concept may not be understood by readers who are not familiar with the VCF format, and therefore should be explained

We agree with the reviewer's advices and add a brief description (line 163) as: "First, each record is associated with a data quality value in the FILTER column, which records the status of this genomic position passing all filters. Usually only qualified records with a "PASS" filter value are retained."

- (page 10) "is homozygous reference alleles" -> "is a homozygous reference allele"

Done. See line 169.

- (page 11) "Meantime" -> "Meanwhile"

Done. See line 202.

- (page 11) Hopefully the pre-defined sampling rate is a parameter to your system?

Yes. the sampling rate can be set via the option –r when running the program.

- (page 11) "equal to the reciprocal of input file number": do you mean "equal to the reciprocal of the number of input files"?

We are sorry for the loose description and have corrected it as the reviewer suggested (line 209).

- (page 12) "a partitioner shuffles": it is not clear what the point of shuffling is here. Shuffling suggests that the order is being changed, as in shuffling a deck of cards. Is that is what is happening here? If not, is there a better way to describe this.

We are sorry for the confusion. Usually a hash-based partitioner shuffles data. Here we use a custom partitioner that keeps the order. So we change shuffle to redirects (line 217).

- (page 13) You use an inconsistent way to number the explanations for each parallelisation technique. For MapReduce, you use "First", "Second". For HBase you use numberings "1) 2)". For Spark you use "Stage I, Stage II". It would be good to be consistent across the manuscript.

We thank the reviewer for the advices. And we change the numbering to make them consistent. We use stage instead of phase in Spark is because stage is a specific term used in Spark literatures for describing groups of steps separated by a data shuffling operation. Other than that, everything is consistent.

- (page 14) "This necessitates the adding of this phase". This sentence seems unusual on its own. Maybe it can be incorporated into the previous sentence, or avoided altogether?

We take the reviewer's advice and incorporate it into the previous sentence (line 246).

- (page 14) "a magnitude faster" -> "and order of magnitude faster"

Done (line 255).

- (page 14) it is not clear what "the normal loading" is.

We are sorry for the confusion.
On line 254
We change it to: "This procedure is therefore at least an order of magnitude faster than the normal loading in which data are loaded sequentially via HBase servers' I/O routines."

- (page 20) "Therefore we are not expecting to see any bottleneck when dealing with even larger scale of data": why not run some much larger tests and report the actual results rather than speculating?

We agree with the reviewer and increase the number of input files to 186. Initially we test on 93 because running cloud cluster is expensive.

- (page 20) In the comparison against a single node implementation, it would be best if you (re)-stated the number of cores used by the single node. Otherwise, saying X-core parallel implementations are Y-times faster is not very helpful. If they use N times as many cores, then we would hope to approach an N times speedup. You could also run some scaling tests on the single-node system.

We thank the reviewer for the suggestion. We added the following for re-state (line 468) the number of cores used by single node: "The single multiway merger is run on a node with the hardware configuration (4 cores and 15G memory) identical to the nodes on which the Apache cluster-based schemas are run."

We have added scaling test on single-node system as described previously, and incorporate the single node cluster of the Apache schemas in the scaling tests.

- (page 22) "We manage to show that all three schemas are _highly_ scalable on both input and data size". I do not think that the reported results warrant the "highly scalable" description for all implementations. The number of cores demonstrated and the size of the input files are not particularly large for modern genomics research. To warrant "highly scalable" would require much larger benchmarks to be reported, and more distinction between strong and weak scaling.

We are sorry for the overstatement and changed “highly scalable” to “scalable” (line 509).

- (page 27) "grateful for" -> "grateful to"

Done. See line 594.

Reviewer #2:

This paper is about a comparison among three Apache big data platforms, MapReduce, HBase and Spark, to perform sorted merging of massive genome-wide data.

The topic is very important for Bioinformatics, the paper is clear, figures and graphs are easy to read, the scalability is well studied and I appreciate that the code is available for testing.

On the dark side, I read in the introduction that the frameworks were tested using two different tests, while basically only the analysis and integration of VCF files has been tested.

We apologize for the confusion. In the introduction, we mention two tests, one is merging multiple VCF files into one VCF with VCFTools as benchmark (results shown in Table 1), and the other is converting and merging VCF files into a TPED file used by PLINK (results shown in figure 5-10). For the first test, we only compared performance of our schemas with VCFTools without scalability tests because the underlying mechanism is same as merging to TPED file which we show detailed results on scalability. The backbone workflow and execution process of all three schemas remain the same in both applications, the only thing changes is the value content of each key-value pairs. So we expect the scalability properties of first application to be identical to the second one. We add a paragraph (line 170) in the “*Data Formats and Operations”* section describing the rules of merging VCF files into a single VCF file and reasons why we skip the scalability tests of this application. Also, on the project website at https://github.com/xsun28/CloudMerge/, we provide the source codes and programs for running both applications.

I honestly don't know if this is enough for a publication in such an important journal. Is a single application enough to say what platform is the best for "Sorting and Merging Massive Omics Data"?

In case the title should be changed to specify the single application described.

Otherwise, in order to be more general about omics data, probably at least another test on a completely different bioinformatic application would be necessary to really describe pros and cons of these three different frameworks. I may suggest something about metagenomics, such as:

Zhou, Wei, et al. "MetaSpark: a spark-based distributed processing tool to recruit metagenomic reads to reference genomes." Bioinformatics 33.7 (2017): 1090-1092.

The reason why we used the broad term in the title is because we think the VCF format is representative of many different types of omics data as they can be thought of as collections of genomic regions along the genome. These files are oftentimes need to be merged (and sorted to keep the order). What is more important is that many of these omics data can be represented as a combination of keys and values. This representation allows the reuse of the backbones of our schemas, and users only need to provide their custom functions of generating the contents of their own keys and values, just as what we did in modifying our first application (VCFs to a single TPED) into the second application (VCFs to a single VCF). But we fully agree with the reviewer that omics data are more than just VCF files or collection of genomic regions. So we took the reviewer's advice and change the title to "… Sorting and Merging Massive **VCF files**" to narrow down and more accurately reflect the types of data that our methods can be applied to. Although this looks like a single application (a very important one), the sorted merging methods we tackled here can be applied in several other scenarios such as finding union or intersection of multiple ChIP-seq peaks.

We also added a paragraph in the Introduction about the potential of additional bioinformatics applications and cited the Zhou et al. paper (line 68) as: "The MetaSpark [16] utilized Spark's distributed data set to recruit large scale of metagenomics reads to reference genomes, achieves better scalability and sensitivity than single-machine based programs." We did not test our method on metagenomics data since our algorithm is designed to carry out sorted merging, which is not the main computational problem in metagenomics. But we do believe the same principles we demonstrated in this study can be applied to address computational problems encountered in other contexts such as some aspects of the metagenomics analyses that involve the key-value model and sorted merging operations.

Another major point is that no related works are presented. The analysis of massive genotyping datasets in VCF format has been addressed at least by another work, which should be discussed and compared in the paper:

O'Brien, Aidan R., et al. "VariantSpark: population scale clustering of genotype information." BMC genomics 16.1 (2015): 1052.

We thank the reviewer for pointing this out. We now cited this paper and a few related papers. Because the purpose of VariantSpark is to cluster variants efficiently, which is different from the purpose of our tools, hence we are unable to conduct a performance comaprison.

Additionally, we add some other papers about applying Apache distributed systems in bioinformatics and WGS:

On line 62: “For example, the Cancer Genome Atlas project made use of Hadoop framework to split genome data into chunks distributed over the cluster for parallel processing.”

On line 64: Add the Seal and Hadoop-BAM software citations.

On line 74: “Although numerous Apache cluster-based applications have already been developed for processing and analyzing large scale of genomics data including ADAM [1],VariantSpark [20], SparkSeq [21], Halvade [22], SeqHBase [23] among others, we believe there are still many opportunities in biomedical data analyses to take advantage of distributed systems as data becomes larger and more complex.”

We also added a review paper (line 54) on this topic in the Introduction Section.

Al last, I think that there is a bit of confusion in the terminology between Hadoop and MapReduced, which should be solved.

We thank the reviewer for raising this issue. Hadoop is Apache’s implementation of MapReduce Framework on YARN and HDFS. We have clarified this in the manuscript on line 105 and 188.

Reviewer #3:

Authors designed, implemented and evaluated three strategies to perform sorted merging of genomic variant data using distributed processing engines.
They demonstrated that proposed approaches outperform typical single-node solutions (such as VCF-tools), and allows to process larger datasets because of their scalability.
The work improves our knowledge in adapting Big Data tools for genomic variant analysis and provide novel, efficient tools for bioinformatics community.

Major comment: Authors did not mention the problem of dealing with multi-allelic sites (genomic positions with more than 2 different alleles), which is especially important when working with large-scale sequencing data.

We thank the reviewer for the advices, we add to the discussion (line 539): “Our implementations automatically take care of multi-allelic positions which are frequent in large scale VCF flies by retaining the information of all alleles until the merging actually occurs.”

Minor comment: Please, synchronized formatting of subsections.

We thank the reviewer for pointing it out, and we have synchronized formatting of subsections.
