# Supplementary material for: Optimized distributed systems achieve significant performance improvement on sorted merging of massive VCF files
Source: Gigascience. 2018 May 10;7(6):giy052. doi: 10.1093/gigascience/giy052 (PMC6007233; doi:10.1093/gigascience/giy052)
Supplement: Supplemental Files [file giy052_supplemental_files.zip › FigureS1.pdf]

MapReduce Schema

```
Phase I
    Mapper
        loading VCF files by parallel mappers
            for each record in each mapper:
                quality check
                    if not pass: continue
                total_record++
                if sampled_record/total_record <= sampling_rate:
                    sampled_record++
                    parse genotype
                    send <pos, chr_n> to partitioner
                output <pos, sample_id+chr_n+ref+genotype> to chr_n directory
                on HDFS
    Partitioner
        for each record:
            sent to reducer of its chr
    Reducer
        for each chr, launch one reducer:
            for each sampled record:
                count++
                if count >= number of input files:
                    count = 0
                    output <pos, null> to chr_n's partition list file in
                    distributed cache

Phase II
    for each chr, launch one MapReduce job:
        for each job:
            Mapper
                loading data from the corresponding chr directory
                for each record:
                    output to TotalOrderPartitioner
            Partitioner
                load its chr's partition list from distributed cache:
                if partition list is empty:
                    Resample records of its chr with higher rate
                for each record
                    find pos_left, pos_right in partition list such that
                    pos_record ∈ [pos_left, pos_right]
                    output record to reducer of this interval
            Reducer
                for each pos interval in partition list, launch one reducer
                    group records by pos
                        join records to TPED format
                        output joined record onto HDFS
```
