# Supplementary material for: Optimized distributed systems achieve significant performance improvement on sorted merging of massive VCF files
Source: Gigascience. 2018 May 10;7(6):giy052. doi: 10.1093/gigascience/giy052 (PMC6007233; doi:10.1093/gigascience/giy052)
Supplement: Supplemental Files [file giy052_supplemental_files.zip › FigureS2.pdf]

**HBase Schema**

```
    if not incremental join start Sampling Phase
            same as Phase I of MapReduce Schema

    else Bulk Loading Phase
        create HBase table with region boundaries = record_i in partition lists where i % {number of regions} == 0
        Mapper
            load data by parallel mappers
                if incremental join:
                    load input files
                    filter out unqualified records
                else:
                    load outputs from Sampling Phase
            for each record in each mapper:
                reformat to HBase compatible key, value
                send to the reducer of the region where the key belongs to
        Reducer
            for each region launch one reducer
            for each record:
                write into HFile of the region
        Loading table
            move HFiles into HBase data directory
            backend sorting records by Log-Structured Merge Tree (LSMT)

    Exporting Phase
        define a scan over the query range
        Mapper
            launch one mapper for each region within the query range
            for each mapper:
                join contiguous records of same pos into a TPED record
                output joined record onto HDFS
```
