# Supplementary material for: Optimized distributed systems achieve significant performance improvement on sorted merging of massive VCF files
Source: Gigascience. 2018 May 10;7(6):giy052. doi: 10.1093/gigascience/giy052 (PMC6007233; doi:10.1093/gigascience/giy052)
Supplement: Supplemental Files [file giy052_supplemental_files.zip › FigureS3.pdf]

Spark Schema

Preprocessing Stage

```
for each HDFS split of input file:
    load as a Spark RDD in Hadoop input format
    for each record in the RDD partition:
        convert to Spark PairRDD record with key and value
        if record is unqualified:
            key and value are set as empty
        else:
            key = chr_pos, value = genotype
    persist RDD in memory
```

Sorting & Merging Stage

```
repartition and sort records in the order of key into children PairRDDs
transform PairRDDs into RDDs of TPED records by partition-preserving mapping
    for each record in a PairRDD partition
        if key_current is empty:
            continue
        if key_current == key_previous:
            add record to record_list
        else:
            join all records in record_list into a TPED record
```

Output Stage

Output PairRDDs of joined records as plain text format onto HDFS
