# Supplementary material for: Optimized distributed systems achieve significant performance improvement on sorted merging of massive VCF files
Source: Gigascience. 2018 May 10;7(6):giy052. doi: 10.1093/gigascience/giy052 (PMC6007233; doi:10.1093/gigascience/giy052)
Supplement: Response_to_Reviewer_Comments_Original_Submission.pdf [file giy052_response_to_reviewer_comments_original_submission.pdf]

Author's Response To Reviewer Comments

Close

Reviewer #1:
This paper is a cross between a case study and an application note. As a case study it considers the task of improving the performance of sorted merging of variant call data using parallel computation. As an application note it provides software implementations of tools which can be downloaded and used by bioinformatics practitioners. Although the case study considers sorting variant call data (VCF files) the techniques developed can easily be extended to other coordinate oriented data sets.

The paper describes in moderate detail the implementation of three different parallelisation techniques based on the Apache distributed platforms: Hadoop (MapReduce), HBase, Spark.

It compares the performance of these implementations for strong and weak scaling (though the authors do not use this terminology) against each other, and also against non-distributed parallel versions using the widely used package VCFTools, and also a custom built multiway-merge implementation. The authors also consider the performance characteristics of different phases of their parallel implementations to see whether any of them might suffer bottlenecks when scaling to larger data sets.

The performance outcomes are fairly predictably in favour of the distributed parallel systems. However, the authors have a tendency to overstate the significance of the outcomes, saying that "Traditional single machine based methods are no longer feasible to process big data due to the prohibitive computation time and I/O bottleneck".

I do not believe that the results of this paper show that single node merging is infeasible, rather they show that distributed parallel techniques can scale to larger data sets. This is something we have known for a long time. The authors also say that "The newly distributed systems have the potential to offer a much needed boost in performance", however, distributed systems are now decades-old ideas. Also, the paper does not really attempt to investigate similar strong and weak scaling for the single node implementations.

We thank the reviewer's comments. We apologize for making the overstatements. We took the reviewer's advice and have rephrased the two sentences as following.

On line 21:
"Traditional single machine based methods are no longer feasible to process big data due to the prohibitive computation time and I/O bottleneck"
Changed to:
"Traditional single machine based methods become increasingly inefficient when processing hundreds or even thousands of VCF files due to the excessive computation time and I/O bottleneck."

On line 23:
"The newly distributed systems have the potential to offer a much needed boost in performance"
Changed to:
"The distributed systems and the more recent cloud-based systems offer an attractive solution."

Following the reviewer's advice, we investigated strong and weak scalabilities for single node

implementation. We implemented a parallel multiway-merger running on a single node to test its performance in terms of strong and weak scaling.

For scaling test, there are three ways to implement the merger on a single node:
1. Simply increasing the number of CPUs of the single node. However, the implementation is based on multiway-merge algorithm, which can only allow one writer (single process or thread) to retrieve records from the underlying priority queue data structure. As a result, when using just single multiway-merger, adding more CPUs won't help improving the performance. However, there are two non-trivial ways for scaling which are illustrated below.

2. We can have multiple multiway-mergers run at the same time where each merger handles the merging of a subset of all files (task-parallelism). Then in the next round, merged files resulted from the previous round are gathered and merged again. This process continues until all files are merged into one file, which will take approximately log(n) rounds where n is the number of input files. This method is essentially our MPI-based HPC implementation running on a single node.

3. We can have multiple multiway-mergers run at the same time where each merger handles a non-overlapping genomic region of the same length from all input files (data-parallelism). Although this strategy sounds promising, it is not quite practical unless the data distribution across all files are uniform or we know the exact data distribution in advance. As a result, it is very difficult to get a balanced workload for each merger unless we sample through all the files to get the data distribution profile. In that case, if we are using single thread/process sampling, this will be slower than the single multiway merge. If we are using multi-thread/process sampling to do this, it essentially becomes the same as our Hadoop implementation running on a single machine but without task coordination offered by YARN. To make a compromise, we can assume the data distribution is uniform and try to assign each merger a genomic region of the same length. Additionally, when reading the input files, we do not want each merger to seek the beginning of its genomic region from the very beginning of the file. Instead we use TabixReader which takes advantage of the Tabix index built on the VCF file to seek directly to the right offset. In addition to the potential workload unbalance problem, this method also has three limitations: 1. Additional time costs are incurred for building the Tabix indices for each file. 2. Tabix can only be used for VCFs, so it cannot be generalized to other data formats. 3. Each process needs to maintain a whole set of file descriptors, Tabix indices, current data blocks of all files in memory, which could cause memory leak or extremely slow garbage collection (GC) time when input data size is large (in our Java implementation, 16 parallel mergers with 93 files consume over 100GB memory. This memory burden may be slightly lower in C/C++ implementation). The following table summarizes the results from weak and strong scaling test running on a r4.8xlarge EC2 instance.

Strong scalability test:
Number of file: 93
# of cores
1
2
4
8
16
Time cost (seconds)
2,410
1,410
959
388

414

From strong scaling test, we can see that as the number of cores increases, the efficiency reduces significantly, indicating poor strong scalability. This is because the more processes, the more imbalanced the workload among processes. Additionally, the saturation of I/O to and from disk as well as higher memory management costs (GC, swap and so on) also contribute to its inefficiency. Results are displayed in Figure 7 in the manuscript.

Weak scalability test:
# of files
10
20
40
80
160
# of cores
1
2
4
8
16
Time cost (seconds)
242
299
334
417
508

From weak scaling test, we can see that although we fix the data size per process, because of the same reasons as in strong scalability test, the efficiency still decrease significantly. . Results are displayed in Figure 8 in the manuscript.

In conclusion, when input data size is relatively small, the parallel multiway-merge has significant performance advantage over cloud-based method because it avoids the data network transmission latency. However, when input data size is very large which is often the case in real applications, the problem of workload imbalance across all processes, I/O saturation and memory burden worsens significantly. As a result, this method no longer scale well.

In the manuscript, we added "Parallel Multiway-Merge and MPI-based High Performance Computing Implementations" (line 298) and "Strong and Weak Scalabilities" (line 347) sections under the Method to describe how they are implemented and how the tests of strong and weak scalabilities are conducted. We also added a "Comparing Strong and Weak Scalabilities between Apache Cluster-based Schemas and Traditional Parallel Methods" (line 395) section under the Results to show and compare the strong and weak scalabilities of Apache cluster-based schemas with parallel multiway-merge and HPC-based implementations. Finally, we compared the execution speed of both parallel multiway-merge and HPC-based implementations with the Apache cluster-based schemas in the "Comparing Execution Speed between Apache Cluster-based Schemas and Traditional Methods" (line 452) section in the Results.

I believe that the authors have also overstated the scalability of the distributed implementations,

saying they have "nice" scalability (nice is a poor choice of adjective in a scientific paper), however, the results shown in figures 5 and 6 show distinct degradation in both strong and weak scaling at higher core counts/input sizes.

We agree with the reviewer and have rephrased the statement to the following (line 379):
“As Figure 6 shows, for all three schemas, given a fixed number of cores, the execution time increases in a slower pace than that of the input data size.”

One thing missing from the paper is a comparison to a distributed parallel version not based on Apache platforms. That is to say, could we achieve similar performance results on a traditional HPC cluster with a shared filesystem to the demonstrated cloud-based solutions? This question is important to consider, because many bioinformatics organisations have access to HPC systems, and heterogenous cloud-HPC platforms are still difficult to manage. It is not clear whether the effort required to implement systems within the Apache frameworks is justified, compared to a comparatively simpler approach on a traditional HPC system.

We thank the reviewer for raising this important question. There are two HPC-based solutions for this problem: data parallelism and task parallelism. However, data parallelism is difficult to achieve because we do not know the data distribution. Randomly splitting and assigning data to HPC nodes causes workload imbalance and jeopardies the overall performance. We could do parallel sampling to acquire the distribution profile, but this will make its workflow the same as that of the MapReduce-based schema with the same operations and the same order: sampling in parallel, dividing the dataset into splits of equal sizes, and assigning the splits to processes to perform the merging. But this implementation is without data locality offered by HDFS and task coordination offered by YARN and thus has a performance no better than the MapReduce-based schema. Consequently, we only implement and test the task parallelism using openMPI as following: firstly, we scatter an approximate number of input files to each HPC process across the whole cluster. Each process fetches the assigned files from the NFS system and do the multiway-merging locally. Secondly, in the following rounds, we adopt a tree-structured merging strategy. To be specific, in the second round, processes whose id number is even (process id starts from 0) retrieve merged files from its adjacent process to the right and do the local multiway-merging. In the third round, processes whose id can be fully divided by 4 retrieve merged results of its adjacent process to the right in the second round. For example, process 4 will receive merged results from process 6. This process continues until all the files are merged into a single file. The total number of rounds is log(n).

Strong scalability test:
Number of file: 93

# of nodes (# of processes)
1 (4)
2 (8)
4 (16)
8 (32)
16 (64)
Time cost (seconds)
17745
9105
5377
3098

2093

In the strong scaling test, the efficiency drops significantly as we add more nodes and processes. This is because although adding nodes lead to a better parallelism, the workload imbalance (because it is almost impossible to assign each node exactly the same number of files) is also increased among nodes in every round. Results are displayed in Figure 7 in the manuscript.

Weak scalability test:
# of files
10
20
40
80
160
# of nodes(# of processes)
1 (4)
2 (8)
4 (16)
8 (32)
16(64)
Time cost (seconds)
1311
1799
2142
2919
3777

In the weak scaling test, we fixed the input data size per node. The efficiency drops even more because of increased number of merging rounds. Results are displayed in Figure 8 in the manuscript.

In conclusion, we showed that sorted merging by traditional HPC (task parallelism) shows inferior scalability and are much slower than the methods running on Apache cluster-based framework.

We have added the description of HPC-based implementation and its tests of strong and weak scalabilities in the "Parallel Multiway-Merge and MPI-based High Performance Computing Implementations" (line 298) section and "Strong and Weak Scalabilities" (line 347) section.

Perhaps the main contribution of this paper is not the resulting software tools, but the insights provided into parallelisation techniques on the Apache cloud-based platforms. Here, the paper exhibits more value as a case study than as an application note. The methods section makes up a considerable portion of the paper, and attempts to explain the main ideas underpinning each implementation, and also contains some useful observations about potential pitfalls in each approach. Generally speaking I find it difficult to closely follow algorithms when they are described as prose, and this paper suffers from a lack of clarity in the explanations of each implementation. Figures 2-4 do help with the presentation, though I wonder whether a pseudo-code style presentation might also be helpful?

We thank the reviewer for the suggestion. We now have included pseudo-code presentation in Figure S1-S3

Overall I think the paper will be of most interest to bioinformatics software implementors who are investigating the parallelisation of tools on cloud-based platforms. It is probably of less interest to bioinformatics practitioners, who are interesting in putting the tools into use.

We agree with the reviewer's comment.

Below are some remarks about the presentation which might improve future revisions:

We really appreciate the reviewer's careful proofreading and specific corrections!

- (page 2) "achieve maximum performance" -> "achieve high performance" (it is not clear that "maximum" performance is well defined)

Done. See line 26.

- there are numerous instance where the definite article "the" is incorrectly used. For example:
(page 3) "of _the_ powerful computing resources" (should be "of powerful computing resources")
(page 5) "applications of Apache big data platform" (should be "of _the_ Apache big data platform")
(page 21) "More specifically, MapReduce-based schema" (should be "More specifically, _the_ MapReduce-based schema")
I have not listed all examples, and recommend a thorough proof read before final submission.

We have conducted a thorough proof read and corrected similar problems.

- (page 4) "takes advantage of" -> "take advantage of" (because you are talking about two different tools)

Done. See line 55.

- (page 4) "researchers have recently started to embrace distributed systems" -> I don't think this is really true. Distributed systems have been in use in bioinformatics for quite some time now. The trend towards cloud-based and hadoop-styled computations is somewhat more recent, but still not new.

We agree with reviewer's opinion.
On line 61:
We change it to: "In bioinformatics, researchers have already started to embrace Apache distributed systems to manage and process large amount of high throughput omics data."

- (page 5) "plenty of" -> "many"

Done. See line 76.

- (page 5) maybe replace "single-threaded" with "sequential"

Done. See line 226.

- (page 6) it is not clear what "working schemas" means here. Also it might be best to describe what you mean by "schemas" in general, since that is an important concept in the paper.

We are sorry for the confusion and in the abstract part (line 24) add: "However, carefully designed

and optimized working flow patterns and execution plans (schemas) are required to take full advantage of the increased computing power while overcoming bottlenecks to achieve high performance.”

- (page 7) "boosts" -> "improves"

Done. See line 114.

- there are numerous instances where pluralisation is done incorrectly. For example:
(page 8) "bottleneck and hotspot can happen" (should be "bottlenecks and hotspots can happen"), and "unbalanced workload" (should be "unbalanced workloads", or "an unbalanced workload")
(page 10) "locations that appears" (should be "locations that appear")
(page 11) "of interests" (should be "of interest")
(page 14) "finishing writings" (should be "finishing writing")
again, a thorough proof reading of the paper before submission should address these issues
- (page 8) "hit" -> "access" (likewise on page 14)
- (page 9) "In a typical WGS" -> "In a typical WGS experiment"
- (page 9) "one of the individual's" -> "one of an individual's"

We have corrected all of these and other places we found.

- (page 10) mentions an "unqualified filter": this concept may not be understood by readers who are not familiar with the VCF format, and therefore should be explained

We agree with the reviewer’s advices and add a brief description (line 163) as: “First, each record is associated with a data quality value in the FILTER column, which records the status of this genomic position passing all filters. Usually only qualified records with a “PASS” filter value are retained.”

- (page 10) "is homozygous reference alleles" -> "is a homozygous reference allele"

Done. See line 169.

- (page 11) "Meantime" -> "Meanwhile"

Done. See line 202.

- (page 11) Hopefully the pre-defined sampling rate is a parameter to your system?

Yes. the sampling rate can be set via the option –r when running the program.

- (page 11) "equal to the reciprocal of input file number": do you mean "equal to the reciprocal of the number of input files"?

We are sorry for the loose description and have corrected it as the reviewer suggested (line 209).

- (page 12) "a partitioner shuffles": it is not clear what the point of shuffling is here. Shuffling suggests that the order is being changed, as in shuffling a deck of cards. Is that is what is happening here? If not, is there a better way to describe this.

We are sorry for the confusion. Usually a hash-based partitioner shuffles data. Here we use a custom partitioner that keeps the order. So we change shuffle to redirects (line 217).

- (page 13) You use an inconsistent way to number the explanations for each parallelisation technique. For MapReduce, you use "First", "Second". For HBase you use numberings "1) 2)". For Spark you use "Stage I, Stage II". It would be good to be consistent across the manuscript.

We thank the reviewer for the advices. And we change the numbering to make them consistent. We use stage instead of phase in Spark is because stage is a specific term used in Spark literatures for describing groups of steps separated by a data shuffling operation. Other than that, everything is consistent.

- (page 14) "This necessitates the adding of this phase". This sentence seems unusual on its own. Maybe it can be incorporated into the previous sentence, or avoided altogether?

We take the reviewer's advice and incorporate it into the previous sentence (line 246).

- (page 14) "a magnitude faster" -> "and order of magnitude faster"

Done (line 255).

- (page 14) it is not clear what "the normal loading" is.

We are sorry for the confusion.
On line 254
We change it to: "This procedure is therefore at least an order of magnitude faster than the normal loading in which data are loaded sequentially via HBase servers' I/O routines."

- (page 20) "Therefore we are not expecting to see any bottleneck when dealing with even larger scale of data": why not run some much larger tests and report the actual results rather than speculating?

We agree with the reviewer and increase the number of input files to 186. Initially we test on 93 because running cloud cluster is expensive.

- (page 20) In the comparison against a single node implementation, it would be best if you (re)-stated the number of cores used by the single node. Otherwise, saying X-core parallel implementations are Y-times faster is not very helpful. If they use N times as many cores, then we would hope to approach an N times speedup. You could also run some scaling tests on the single-node system.

We thank the reviewer for the suggestion. We added the following for re-state (line 467) the number of cores used by single node: "The single multiway merger is run on a node with the hardware configuration (4 cores and 15G memory) identical to the nodes on which the Apache cluster-based schemas are run."

We have added scaling test on single-node system as described previously, and incorporate the single node cluster of the Apache schemas in the scaling tests.

- (page 22) "We manage to show that all three schemas are _highly_ scalable on both input and data size". I do not think that the reported results warrant the "highly scalable" description for all implementations. The number of cores demonstrated and the size of the input files are not particularly large for modern genomics research. To warrant "highly scalable" would require much

larger benchmarks to be reported, and more distinction between strong and weak scaling.

We are sorry for the overstatement and changed “highly scalable” to “scalable” (line 508).

- (page 27) "grateful for" -> "grateful to"

Done. See line 593.

Reviewer #2:
This paper is about a comparison among three Apache big data platforms, MapReduce, HBase and Spark, to perform sorted merging of massive genome-wide data.

The topic is very important for Bioinformatics, the paper is clear, figures and graphs are easy to read, the scalability is well studied and I appreciate that the code is available for testing.

On the dark side, I read in the introduction that the frameworks were tested using two different tests, while basically only the analysis and integration of VCF files has been tested.

We apologize for the confusion. In the introduction, we mention two tests, one is merging multiple VCF files into one VCF with VCFTools as benchmark (results shown in Table 1), and the other is converting and merging VCF files into a TPED file used by PLINK (results shown in figure 5-10). For the first test, we only compared performance of our schemas with VCFTools without scalability tests because the underlying mechanism is same as merging to TPED file which we show detailed results on scalability. The backbone workflow and execution process of all three schemas remain the same in both applications, the only thing changes is the value content of each key-value pairs. So we expect the scalability properties of first application to be identical to the second one. We add a paragraph (line 170) in the “Data Formats and Operations” section describing the rules of merging VCF files into a single VCF file and reasons why we skip the scalability tests of this application. Also, on the project website at https://github.com/xsun28/CloudMerge/, we provide the source codes and programs for running both applications.

I honestly don't know if this is enough for a publication in such an important journal. Is a single application enough to say what platform is the best for "Sorting and Merging Massive Omics Data"?

In case the title should be changed to specify the single application described.

Otherwise, in order to be more general about omics data, probably at least another test on a completely different bioinformatic application would be necessary to really describe pros and cons of these three different frameworks. I may suggest something about metagenomics, such as:

Zhou, Wei, et al. "MetaSpark: a spark-based distributed processing tool to recruit metagenomic reads to reference genomes." Bioinformatics 33.7 (2017): 1090-1092.

The reason why we used the broad term in the title is because we think the VCF format is representative of many different types of omics data as they can be thought of as collections of genomic regions along the genome. These files are oftentimes need to be merged (and sorted to keep the order). What is more important is that many of these omics data can be represented as a combination of keys and values. This representation allows the reuse of the backbones of our

schemas, and users only need to provide their custom functions of generating the contents of their own keys and values, just as what we did in modifying our first application (VCFs to a single TPED) into the second application (VCFs to a single VCF). But we fully agree with the reviewer that omics data are more than just VCF files or collection of genomic regions. So we took the reviewer's advice and change the title to "… Sorting and Merging Massive VCF files" to narrow down and more accurately reflect the types of data that our methods can be applied to. Although this looks like a single application (a very important one), the sorted merging methods we tackled here can be applied in several other scenarios such as finding union or intersection of multiple ChIP-seq peaks.

We also added a paragraph in the Introduction about the potential of additional bioinformatics applications and cited the Zhou et al. paper (line 68) as: "The MetaSpark [16] utilized Spark's distributed data set to recruit large scale of metagenomics reads to reference genomes, achieves better scalability and sensitivity than single-machine based programs." We did not test our method on metagenomics data since our algorithm is designed to carry out sorted merging, which is not the main computational problem in metagenomics. But we do believe the same principles we demonstrated in this study can be applied to address computational problems encountered in other contexts such as some aspects of the metagenomics analyses that involve the key-value model and sorted merging operations.

Another major point is that no related works are presented. The analysis of massive genotyping datasets in VCF format has been addressed at least by another work, which should be discussed and compared in the paper:

O'Brien, Aidan R., et al. "VariantSpark: population scale clustering of genotype information." BMC genomics 16.1 (2015): 1052.

We thank the reviewer for pointing this out. We now cited this paper and a few related papers. Because the purpose of VariantSpark is to cluster variants efficiently, which is different from the purpose of our tools, hence we are unable to conduct a performance comaprison.

Additionally, we add some other papers about applying Apache distributed systems in bioinformatics and WGS:

On line 62: "For example, the Cancer Genome Atlas project made use of Hadoop framework to split genome data into chunks distributed over the cluster for parallel processing."

On line 64: Add the Seal and Hadoop-BAM software citations.

On line 74: "Although numerous Apache cluster-based applications have already been developed for processing and analyzing large scale of genomics data including ADAM [1],VariantSpark [20], SparkSeq [21], Halvade [22], SeqHBase [23] among others, we believe there are still many opportunities in biomedical data analyses to take advantage of distributed systems as data becomes larger and more complex."

We also added a review paper (line 54) on this topic in the Introduction Section.

Al last, I think that there is a bit of confusion in the terminology between Hadoop and MapReduced, which should be solved.

We thank the reviewer for raising this issue. Hadoop is Apache's implementation of MapReduce Framework on YARN and HDFS. We have clarified this in the manuscript on line 105 and 188.

Reviewer #3:
Authors designed, implemented and evaluated three strategies to perform sorted merging of genomic variant data using distributed processing engines.
They demonstrated that proposed approaches outperform typical single-node solutions (such as VCF-tools), and allows to process larger datasets because of their scalability.
The work improves our knowledge in adapting Big Data tools for genomic variant analysis and provide novel, efficient tools for bioinformatics community.

Major comment: Authors did not mention the problem of dealing with multi-allelic sites (genomic positions with more than 2 different alleles), which is especially important when working with large-scale sequencing data.

We thank the reviewer for the advices, we add to the discussion (line 538): “Our implementations automatically take care of multi-allelic positions which are frequent in large scale VCF flies by retaining the information of all alleles until the merging actually occurs.”

Minor comment: Please, synchronized formatting of subsections.

We thank the reviewer for pointing it out, and we have synchronized formatting of subsections.

Close
