# Supplementary material for: Optimized distributed systems achieve significant performance improvement on sorted merging of massive VCF files
Source: Gigascience. 2018 May 10;7(6):giy052. doi: 10.1093/gigascience/giy052 (PMC6007233; doi:10.1093/gigascience/giy052)
Supplement: Response_to_Reviewer_Comments_Revision_1.pdf [file giy052_response_to_reviewer_comments_revision_1.pdf]

Author's Response To Reviewer Comments

Close

Major modifications in this revision are summarized below:

• Corrected grammar mistakes and minor wording issues.
• Add members of CAAPA consortium to authorship.
• Add "Ethics Approval and Consent to Participate" section.
• Add "Funding" section.
• Add CAAPA Consortium members to the "Authors Contributions" section.
• Add CAAPA Consortium members in the "Acknowledgements" section
• Add a complete list of CAAPA Consortium members and their affiliations at the end
• Added our test datasets and codes to the GigaDB database.
• Added a citation of test datasets in GigaDB to the reference list, and cited it in appropriate places in the manuscript.
• Added all URLs as references in the bibliography, and cited them in the corresponding places in the manuscript.

Below we present itemized responses to all the comments, organized by editors and reviewers. The reviewers' comments are in bold. Our responses are in dark blue color.

Editor comments:

Before we hand over your manuscript to our production team:

- please go over the list of minor wording issues below, kindly provided by reviewer 1, and correct them in a revised submission.

Done

- please add a statement on ethical considerations to the manuscript. You are using encrypted VCFs, but as the original data set from reference [33] is under an authorized access scheme, if I understand correctly, I assume you needed IRB approval to access and work with the un-encrypted data for this project? Please also clarify in the manuscript whether this encrypted use of the subjects' data is covered by the consent they gave.

Done, please see "Ethics Approval and Consent to Participate" section in the manuscript.

Regarding your code and test data, one of our data curators will contact you shortly. Usually we host an archival copy of any code and test data in our repository GigaDB, which will be cited in the manuscript. Our data curators will discuss this with you.

Done

Please include a citation to any upcoming GigaDB dataset to your reference list (including the DOI link you will get from our data curators), and please cite this in the data availability section and elswehere in the manuscript, where appropriate.

Please follow this example format for the reference:

[xx] Author1 N, Author2 N, AuthorX N. Supporting data for "Title of your manuscript". GigaScience Database. 2018. http://dx.doi.orgxxxxxxxxxxxx

(If you don't have a GigaDB doi at the time of resubmission, please leave the "dummy" version and we can exchange this for you.)

Please see reference 43 in the manuscript

Finally, a very minor point: Please include all URLs (except the "availability" section") as references in the bibliography, and cite them from the text rather than inserting them directly.

Done. On line 321:
The source codes are available at our GitHub website [35] (CloudMerge; RRID: SCR_016051).

Reviewer #1:
This paper has undergone substantial improvements since the original submission and the authors are to be commended on their efforts to address all the main issues raised in the initial review. I am satisfied that all of my concerns from the initial review have been adequately addressed, and I am happy to recommend that this paper is accepted for publication.

We are grateful to the reviewer for the comment.

I have a few minor comments below that the authors might wish to consider when editing the final version: "merging a large number of Variant Call Format (VCF) files are frequently encountered" -> "merging a large number of Variant Call Format (VCF) files is frequently encountered".

Done. See line 19.

"when processing hundreds or even thousands of VCF files" -> "when processing large volumes of VCF files".

Done. See line 22.

"The distributed systems and the more recent cloud-based systems" -> "Distributed systems and more recent cloud-based systems".

Done. See line 23.

"working flow" -> "workflow".

Done. See line 24.

"Apache Foundation has" -> "The Apache Foundation"

Done. See line 56.

"took advantage" -> "take advantage"

Thanks for brings this out. It is done, see line 66. In addition, we also make additional similar changes

from past tense to current tense:
On line 64, “made” -> “make”.
On line 68, “adopted” -> “adopts”.
On line 69, “utilized” -> “utilizes”.

Are two citations really needed for the "sorted full-outer-joining problem"? If it is well known, as the authors claim, then one citation should be sufficient.

Yes, we agree with the reviewer, and delete one reference: “28. Silberschatz A, Korth HF and Sudarshan S. DatabaseSystem Concepts. 2010.”

"cumbersome" is probably the wrong word to describe the behaviour of PLINK and VCFTools on moderate numbers of input files. Cumbersome suggests that they are awkward or difficult to use, but really the problem is that their performance is unacceptable.

Yes, we agree with the reviewer.
On line 90:
“Currently, they are handled by software such as VCFTools [28] and PLINK, which become very cumbersome even in the face of a moderate number of VCF files.”
Changed to:
Currently, they are handled by software such as VCFTools [28] and PLINK, which become considerably inefficient even in the face of a moderate number of VCF files.

"literally makes it sequential on writing" -> "makes it sequential on writing" (remove "literally", it is redundant) "

Done. See line 94.

and memory limitation" -> "and memory limits"

Done. See line 96.

"ideally fit" -> "is an ideal fit"

Done. See line 131.

"megabyte in size" -> "megabytes in size" (plural). Maybe use MB instead, to be consistent with the rest of the article using GB.

Done. See line 163.

"Key-value pais" -> "key-value pairs" (capitalisation)

Done. See line 209.

It is not clear what this means "we only need to merge records from selected chromosomes of interest rather than from all of them". Can you please clarify?

We thank the reviewer 1 for bringing this up. Here we mean if we are only interested in the merged results of some specific chromosomes, say chr1-chr3, then we can just merge the records in the corresponding bins, instead of merging the records of all chromosomes. And on line 210, we have

rephrased this sentence from
“With this grouping, we only need to merge records from selected chromosomes of interest rather than from all of them.”
to
“With this grouping, if SNPs of interest located in a few selected chromosomes only, we can choose to just merge records from these selected chromosomes rather than from all chromosomes.”

delete: "which necessitates the adding of this phase"

Done. See line 261.

"finishing writings" -> "finishing writing" (not plural)

Done. See line 269.

It is likely that the HPC tests (namely the MPI version) would have performed better on a system with a high-performance file system such as GPFS or Lustre instead of NFS.

We totally agree with reviewer 1’s opinion. Both Lustre and GPFS has better I/O scalability than NFS. And we expect our HPC benchmark would perform better using these file systems. However, I/O is not only the reason why the HPC benchmark does not scale well. Rather, the increasing in the number of merging rounds when increasing the number of input files is the main reason for decreasing efficiency. So we expect the scalability will improve to some extent but not too much when running it with the GPFS or Lustre file system. Another reason we choose NFS because we test our benchmark using StarCluster, which currently doesn’t support either GPFS or Lustre.

Use GB for gigabytes instead of G.

Done.

Close
