# Supplementary material for: Optimized distributed systems achieve significant performance improvement on sorted merging of massive VCF files
Source: Gigascience. 2018 May 10;7(6):giy052. doi: 10.1093/gigascience/giy052 (PMC6007233; doi:10.1093/gigascience/giy052)
Supplement: Reviewer_1_Report_(Original_Submission) -- Bernie Pope — 11/5/2017 Reviewed [file giy052_reviewer_1_report_(original_submission).pdf]

Reviewer Report

Title: Optimized Distributed Systems Achieve Significant Performance Improvement on Sorted Merging of Massive VCF Files

Version: Original Submission **Date:** 11/5/2017

Reviewer name: Bernie Pope

Reviewer Comments to Author:

This paper is a cross between a case study and an application note. As a case study it considers the task of improving the performance of sorted merging of variant call data using parallel computation. As an application note it provides software implementations of tools which can be downloaded and used by bioinformatics practitioners. Although the case study considers sorting variant call data (VCF files) the techniques developed can easily be extended to other coordinate oriented data sets.The paper describes in moderate detail the implementation of three different parallelisation techniques based on the Apache distributed platforms: Hadoop (MapReduce), HBase, Spark.It compares the performance of these implementations for strong and weak scaling (though the authors do not use this terminology) against each other, and also against non-distributed parallel versions using the widely used package VCFTools, and also a custom built multiway-merge implementation. The authors also consider the performance characteristics of different phases of their parallel implementations to see whether any of them might suffer bottlenecks when scaling to larger data sets.The performance outcomes are fairly predictably in favour of the distributed parallel systems. However, the authors have a tendency to overstate the significance of the outcomes, saying that "Traditional single machine based methods are no longer feasible to process big data due to the prohibitive computation time and I/O bottleneck". I do not believe that the results of this paper show that single node merging is infeasible, rather they show that distributed parallel techniques can scale to larger data sets. This is something we have known for a long time. The authors also say that "The newly distributed systems have the potential to offer a much needed boost in performance", however, distributed systems are now decades-old ideas. Also, the paper does not really attempt to investigate similar strong and weak scaling for the single node implementations.I believe that the authors have also overstated the scalability of the distributed implementations, saying they have "nice" scalability (nice is a poor choice of adjective in a scientific paper), however, the results shown in figures 5 and 6 show distinct degradation in both strong and weak scaling at higher core counts/input sizes. One thing missing from the paper is a comparison to a distributed parallel version not based on Apache platforms. That is to say, could we achieve similar performance results on a traditional HPC cluster with a shared filesystem to the demonstrated cloud-based solutions? This question is important to consider, because many bioinformatics organisations have access to HPC systems, and heterogenous cloud-HPC platforms are still difficult to manage. It is not clear whether the effort required to implement systems within the Apache frameworks is justified, compared to a comparatively simpler approach on a traditional HPC system.Perhaps the main contribution of this paper is not the resulting software tools, but the insights provided into parallelisation techniques on the Apache cloud-based platforms. Here, the paper exhibits more value as a case study than as an application note. The methods section makes up a considerable portion of the paper, and attempts to explain the main ideas underpinning each implementation, and also contains some useful observations about potential pitfalls in each approach. Generally speaking I find it difficult to closely follow algorithms when they are described as prose, and this paper suffers from a lack of clarity in the explanations of each implementation. Figures 2-4 do help with the presentation, though I wonder whether a pseudo-code style presentation might also be helpful?Overall I think the paper will be of most interest to bioinformatics software implementors who are investigating the parallelisation of tools on cloud-based platforms. It is probably of less interest to bioinformatics practitioners, who are interesting in putting the tools into use.Below are some remarks about the presentation which might improve future revisions:- (page 2) "achieve maximum performance" -> "achieve high performance" (it is not clear that "maximum" performance is well defined)- there are numerous instance where the definite article "the" is incorrectly used. For example:(page 3) "of _the_

powerful computing resources" (should be "of powerful computing resources")(page 5) "applications of Apache big data platform" (should be "of _the_ Apache big data platform")(page 21) "More specifically, MapReduce-based schema" (should be "More specifically, _the_ MapReduce-based schema")I have not listed all examples, and recommend a thorough proof read before final submission.- (page 4) "takes advantage of" -> "take advantage of" (because you are talking about two different tools)- (page 4) "researchers have recently started to embrace distributed systems" -> I don't think this is really true. Distributed systems have been in use in bioinformatics for quite some time now. The trend towards cloud-based and hadoop-styled computations is somewhat more recent, but still not new.- (page 5) "plenty of" -> "many"- (page 5) maybe replace "single-threaded" with "sequential"- (page 6) it is not clear what "working schemas" means here. Also it might be best to describe what you mean by "schemas" in general, since that is an important concept in the paper.- (page 7) "boosts" -> "improves"- there are numerous instances where pluralisation is done incorrectly. For example:(page 8) "bottleneck and hotspot can happen" (should be "bottlenecks and hotspots can happen"), and "unbalanced workload" (should be "unbalanced workloads", or "an unbalanced workload")(page 10) "locations that appears" (should be "locations that appear")(page 11) "of interests" (should be "of interest")(page 14) "finishing writings" (should be "finishing writing")again, a thorough proof reading of the paper before submission should address these issues- (page 8) "hit" -> "access" (likewise on page 14)- (page 9) "In a typical WGS" -> "In a typical WGS experiment"- (page 9) "one of the individual's" -> "one of an individual's"- (page 10) mentions an "unqualified filter": this concept may not be understood by readers who are not familiar with the VCF format, and therefore should be explained- (page 10) "is homozygous reference alleles" -> "is a homozygous reference allele"- (page 11) "Meantime" -> "Meanwhile"- (page 11) Hopefully the pre-defined sampling rate is a parameter to your system?- (page 11) "equal to the reciprocal of input file number": do you mean "equal to the reciprocal of the number of input files"?- (page 12) "a partitioner shuffles": it is not clear what the point of shuffling is here. Shuffling suggests that the order is being changed, as in shuffling a deck of cards. Is that is what is happening here? If not, is there a better way to describe this.- (page 13) You use an inconsistent way to number the explanations for each parallelisation technique. For MapReduce, you use "First", "Second". For HBase you use numberings "1) 2)". For Spark you use "Stage I, Stage II". It would be good to be consistent across the manuscript.- (page 14) "This necessitates the adding of this phase". This sentence seems unusual on its own. Maybe it can be incorporated into the previous sentence, or avoided altogether?- (page 14) "a magnitude faster" -> "and order of magnitude faster"- (page 14) it is not clear what "the normal loading" is.- (page 20) "Therefore we are not expecting to see any bottleneck when dealing with even larger scale of data": why not run some much larger tests and report the actual results rather than speculating?- (page 20) In the comparison against a single node implementation, it would be best if you (re)-stated the number of cores used by the single node. Otherwise, saying X-core parallel implementations are Y-times faster is not very helpful. If they use N times as many cores, then we would hope to approach an N times speedup. You could also run some scaling tests on the single-node system.- (page 22) "We manage to show that all three schemas are _highly_ scalable on both input and data size". I do not think that the reported results warrant the "highly scalable" description for all implementations. The number of cores demonstrated and the size of the input files are not particularly large for modern genomics research. To warrant "highly scalable" would require much larger benchmarks to be reported, and more distinction between strong and weak scaling.- (page 27) "grateful for" -> "grateful to"

Level of Interest

Please indicate how interesting you found the manuscript: An article whose findings are important to those with closely related research interests

Quality of Written English

Please indicate the quality of language in the manuscript: Needs some language corrections before being published

Declaration of Competing Interests

Please complete a declaration of competing interests, considering the following questions:

- Have you in the past five years received reimbursements, fees, funding, or salary from an organisation that may in any way gain or lose financially from the publication of this manuscript, either now or in the future?
- Do you hold any stocks or shares in an organisation that may in any way gain or lose financially from the publication of this manuscript, either now or in the future?
- Do you hold or are you currently applying for any patents relating to the content of the manuscript?
- Have you received reimbursements, fees, funding, or salary from an organization that holds or has applied for patents relating to the content of the manuscript?
- Do you have any other financial competing interests?
- Do you have any non-financial competing interests in relation to this paper?

If you can answer no to all of the above, write 'I declare that I have no competing interests' below. If your reply is yes to any, please give details below.

I declare that I have no competing interests

I agree to the open peer review policy of the journal. I understand that my name will be included on my report to the authors and, if the manuscript is accepted for publication, my named report including any attachments I upload will be posted on the website along with the authors' responses. I agree for my report to be made available under an Open Access Creative Commons CC-BY license (http://creativecommons.org/licenses/by/4.0/). I understand that any comments which I do not wish to be included in my named report can be included as confidential comments to the editors, which will not be published.

I agree to the open peer review policy of the journal

To further support our reviewers, we have joined with Publons, where you can gain additional credit to further highlight your hard work (see: https://publons.com/journal/530/gigascience). On publication of this paper, your review will be automatically added to Publons, you can then choose whether or not to claim your Publons credit. I understand this statement.

Yes
