# Supplementary material for: Optimized distributed systems achieve significant performance improvement on sorted merging of massive VCF files
Source: Gigascience. 2018 May 10;7(6):giy052. doi: 10.1093/gigascience/giy052 (PMC6007233; doi:10.1093/gigascience/giy052)
Supplement: Reviewer_1_Report_(Revision_1) -- Bernie Pope — 2/26/2018 Reviewed [file giy052_reviewer_1_report_(revision_1).pdf]

Reviewer Report

Title: Optimized Distributed Systems Achieve Significant Performance Improvement on Sorted Merging of Massive VCF Files

Version: Revision 1 Date: 2/26/2018

Reviewer name: Bernie Pope

Reviewer Comments to Author:

This paper has undergone substantial improvements since the original submission and the authors are to be commended on their efforts to address all the main issues raised in the initial review.I am satisfied that all of my concerns from the initial review have been adequately addressed, and I am happy to recommend that this paper is accepted for publication.I have a few minor comments below that the authors might wish to consider when editing the final version:"merging a large number of Variant Call Format (VCF) files are frequently encountered" -> "merging a large number of Variant Call Format (VCF) files is frequently encountered"."when processing hundreds or even thousands of VCF files" -> "when processing large volumes of VCF files""The distributed systems and the more recent cloud-based systems" -> "Distributed systems and more recent cloud-based systems""working flow" -> "workflow""Apache Foundation has" -> "The Apache Foundation""took advantage" -> "take advantage"Are two citations really needed for the "sorted full-outer-joining problem"? If it is well known, as the authors claim, then one citation should be sufficient."cumbersome" is probably the wrong word to describe the behaviour of PLINK and VCFTools on moderate numbers of input files. Cumbersome suggests that they are awkward or difficult to use, but really the problem is that their performance is unacceptable."literally makes it sequential on writing" -> "makes it sequential on writing" (remove "literally", it is redundant)"and memory limitation" -> "and memory limits""ideally fit" -> "is an ideal fit""megabyte in size" -> "megabytes in size" (plural). Maybe use MB instead, to be consistent with the rest of the article using GB."Key-value pais" -> "key-value pairs" (capitalisation)It is not clear what this means "we only need to merge records from selected chromosomes of interest rather than from all of them". Can you please clarify?delete: "which necessitates the adding of this phase""finishing writings" -> "finishing writing" (not plural)It is likely that the HPC tests (namely the MPI version) would have performed better on a system with a high-performance file system such as GPFS or Lustre instead of NFS.Use GB for gigabytes instead of G.

Level of Interest

Please indicate how interesting you found the manuscript: An article whose findings are important to those with closely related research interests

Quality of Written English

Please indicate the quality of language in the manuscript: Acceptable

Declaration of Competing Interests

Please complete a declaration of competing interests, considering the following questions:

- Have you in the past five years received reimbursements, fees, funding, or salary from an organisation that may in any way gain or lose financially from the publication of this manuscript, either now or in the future?
- Do you hold any stocks or shares in an organisation that may in any way gain or lose financially from the publication of this manuscript, either now or in the future?
- Do you hold or are you currently applying for any patents relating to the content of the manuscript?
- Have you received reimbursements, fees, funding, or salary from an organization that holds or has applied for patents relating to the content of the manuscript?
- Do you have any other financial competing interests?
- Do you have any non-financial competing interests in relation to this paper?

If you can answer no to all of the above, write 'I declare that I have no competing interests' below. If your reply is yes to any, please give details below.

I declare that I have no competing interests

I agree to the open peer review policy of the journal. I understand that my name will be included on my report to the authors and, if the manuscript is accepted for publication, my named report including any attachments I upload will be posted on the website along with the authors' responses. I agree for my report to be made available under an Open Access Creative Commons CC-BY license (http://creativecommons.org/licenses/by/4.0/). I understand that any comments which I do not wish to be included in my named report can be included as confidential comments to the editors, which will not be published.

I agree to the open peer review policy of the journal

To further support our reviewers, we have joined with Publons, where you can gain additional credit to further highlight your hard work (see: https://publons.com/journal/530/gigascience). On publication of this paper, your review will be automatically added to Publons, you can then choose whether or not to claim your Publons credit. I understand this statement.

Yes
