# Supplementary material for: Optimized distributed systems achieve significant performance improvement on sorted merging of massive VCF files
Source: Gigascience. 2018 May 10;7(6):giy052. doi: 10.1093/gigascience/giy052 (PMC6007233; doi:10.1093/gigascience/giy052)
Supplement: Reviewer_2_Report_(Original_Submission) -- Ivan Merelli — 11/19/2017 Reviewed [file giy052_reviewer_2_report_(original_submission).pdf]

Reviewer Report

Title: Optimized Distributed Systems Achieve Significant Performance Improvement on Sorted Merging of Massive VCF Files

Version: Original Submission **Date:** 11/19/2017

Reviewer name: Ivan Merelli

Reviewer Comments to Author:

This paper is about a comparison among three Apache big data platforms, MapReduce, HBase and Spark, to perform sorted merging of massive genome-wide data. The topic is very important for Bioinformatics, the paper is clear, figures and graphs are easy to read, the scalability is well studied and I appreciate that the code is available for testing. On the dark side, I read in the introduction that the frameworks were tested using two different tests, while basically only the analysis and integration of VCF files has been tested. I honestly don't know if this is enough for a publication in such an important journal. Is a single application enough to say what platform is the best for "Sorting and Merging Massive Omics Data"? In case the title should be changed to specify the single application described. Otherwise, in order to be more general about omics data, probably at least another test on a completely different bioinformatic application would be necessary to really describe pros and cons of these three different frameworks. I may suggest something about metagenomics, such as:Zhou, Wei, et al. "MetaSpark: a spark-based distributed processing tool to recruit metagenomic reads to reference genomes." Bioinformatics 33.7 (2017): 1090-1092.Another major point is that no related works are presented. The analysis of massive genotyping datasets in VCF format has been addressed at least by another work, which should be discussed and compared in the paper:O'Brien, Aidan R., et al. "VariantSpark: population scale clustering of genotype information." BMC genomics 16.1 (2015): 1052.Al last, I think that there is a bit of confusion in the terminology between Hadoop and MapReduced, which should be solved.

Level of Interest

Please indicate how interesting you found the manuscript: An article of importance in its field

Quality of Written English

Please indicate the quality of language in the manuscript: Acceptable

Declaration of Competing Interests

Please complete a declaration of competing interests, considering the following questions:

- Have you in the past five years received reimbursements, fees, funding, or salary from an organisation that may in any way gain or lose financially from the publication of this manuscript, either now or in the future?
- Do you hold any stocks or shares in an organisation that may in any way gain or lose financially from the publication of this manuscript, either now or in the future?

- Do you hold or are you currently applying for any patents relating to the content of the manuscript?
- Have you received reimbursements, fees, funding, or salary from an organization that holds or has applied for patents relating to the content of the manuscript?
- Do you have any other financial competing interests?
- Do you have any non-financial competing interests in relation to this paper?

If you can answer no to all of the above, write 'I declare that I have no competing interests' below. If your reply is yes to any, please give details below.

I declare that I have no competing interests

I agree to the open peer review policy of the journal. I understand that my name will be included on my report to the authors and, if the manuscript is accepted for publication, my named report including any attachments I upload will be posted on the website along with the authors' responses. I agree for my report to be made available under an Open Access Creative Commons CC-BY license (http://creativecommons.org/licenses/by/4.0/). I understand that any comments which I do not wish to be included in my named report can be included as confidential comments to the editors, which will not be published.

I agree to the open peer review policy of the journal

To further support our reviewers, we have joined with Publons, where you can gain additional credit to further highlight your hard work (see: https://publons.com/journal/530/gigascience). On publication of this paper, your review will be automatically added to Publons, you can then choose whether or not to claim your Publons credit. I understand this statement.

Yes
