# Supplementary material for: Optimized distributed systems achieve significant performance improvement on sorted merging of massive VCF files
Source: Gigascience. 2018 May 10;7(6):giy052. doi: 10.1093/gigascience/giy052 (PMC6007233; doi:10.1093/gigascience/giy052)
Supplement: Reviewer_3_Report_(Original_Submission) -- Tomasz Gambin — 12/11/2017 Reviewed [file giy052_reviewer_3_report_(original_submission).pdf]

Reviewer Report

Title: Optimized Distributed Systems Achieve Significant Performance Improvement on Sorted Merging of Massive VCF Files

Version: Original Submission **Date:** 12/11/2017

Reviewer name: Tomasz Gambin

Reviewer Comments to Author:

Authors designed, implemented and evaluated three strategies to perform sorted merging of genomic variant data using distributed processing engines. They demonstrated that proposed approaches outperform typical single-node solutions (such as VCF-tools), and allows to process larger datasets because of their scalability. The work improves our knowledge in adapting Big Data tools for genomic variant analysis and provide novel, efficient tools for bioinformatics community. Major comment: Authors did not mention the problem of dealing with multi-allelic sites (genomic positions with more than 2 different alleles), which is especially important when working with large-scale sequencing data. Minor comment: Please, synchronized formatting of subsections.

Level of Interest

Please indicate how interesting you found the manuscript: An article of importance in its field

Quality of Written English

Please indicate the quality of language in the manuscript: Needs some language corrections before being published

Declaration of Competing Interests

Please complete a declaration of competing interests, considering the following questions:

- Have you in the past five years received reimbursements, fees, funding, or salary from an organisation that may in any way gain or lose financially from the publication of this manuscript, either now or in the future?
- Do you hold any stocks or shares in an organisation that may in any way gain or lose financially from the publication of this manuscript, either now or in the future?
- Do you hold or are you currently applying for any patents relating to the content of the manuscript?
- Have you received reimbursements, fees, funding, or salary from an organization that holds or has applied for patents relating to the content of the manuscript?
- Do you have any other financial competing interests?

- Do you have any non-financial competing interests in relation to this paper?

If you can answer no to all of the above, write 'I declare that I have no competing interests' below. If your reply is yes to any, please give details below.

I declare that I have no competing interests

I agree to the open peer review policy of the journal. I understand that my name will be included on my report to the authors and, if the manuscript is accepted for publication, my named report including any attachments I upload will be posted on the website along with the authors' responses. I agree for my report to be made available under an Open Access Creative Commons CC-BY license (http://creativecommons.org/licenses/by/4.0/). I understand that any comments which I do not wish to be included in my named report can be included as confidential comments to the editors, which will not be published.

I agree to the open peer review policy of the journal

To further support our reviewers, we have joined with Publons, where you can gain additional credit to further highlight your hard work (see: https://publons.com/journal/530/gigascience). On publication of this paper, your review will be automatically added to Publons, you can then choose whether or not to claim your Publons credit. I understand this statement.

Yes
